# Postsynaptic frequency filters shaped by the interplay of synaptic short-term plasticity and cellular time scales

**DOI:** 10.1007/s10827-025-00908-3

**Published:** 2025-10-21

**Authors:** Yugarshi Mondal, Guillermo Villanueva Benito, Rodrigo F. O. Pena, Horacio G. Rotstein

**Affiliations:** 1 Department of Mathematics and Statistics, Stony Brook University, Stony Brook, NY USA; 2 Present Address: Volen Center for Complex Systems, Brandeis University, Waltham, MA USA; 3 Department of Mathematics, Universitat Politécnica de Catalunya, Barcelona, Spain; 4 Present Address: Barcelona Institute for Global Health (ISGlobal), Barcelona, Spain; 5 Federated Department of Biological Sciences, New Jersey Institute of Technology and Rutgers University, Newark, NJ USA; 6 Present Address: Department of Biological Sciences, Florida Atlantic University, Boca Raton, FL USA; 7 Graduate Faculty, Graduate Program in Neuroscience, Rutgers University, Newark, NJ USA

**Keywords:** Preferred frequency responses, Synaptic resonance, Postsynaptic resonance, Low-pass filters, Band-pass filters, High-pass filters, Neuronal variability

## Abstract

Neuronal frequency filters can be thought of as constituent building blocks underlying the ability of neuronal systems to process information, generate rhythms and perform computations. How neuronal filters are generated by the concerted activity of a multiplicity of processes (e.g., electric circuit, history-dependent) and interacting time scales within and across levels of neuronal network organization is poorly understood. In this paper, we use mathematical modeling, numerical simulations and analytical calculations of the postsynaptic response to presynaptic spike trains to address this issue in a basic feedforward network motif in the presence of synaptic short-term plasticity (STP, depression and facilitation). The network motif consists of a presynaptic spike-train, a postsynaptic passive cell, and an excitatory (AMPA) chemical synapse. The dynamics of each network component are controlled by one or more time scales. We explain the mechanisms by which the participating time scales shape the neuronal filters at the (i) synaptic update level (the target of the synaptic variable in response to presynaptic spikes), which is shaped by STP, (ii) the synaptic level, and (iii) the postsynaptic membrane potential (PSP) level. We focus on three metrics that gives rise to three types of profiles (curves of the corresponding metrics as a function of the spike-train input frequency or firing rate): (i) peak profiles, (ii) peak-to-trough amplitude profiles, and (iii) phase profiles. The effects of STP are present at the synaptic update level and are communicated to the synaptic level where they interact with the synaptic time scales. The PSP filters result from the interaction between these variables and time scales and the biophysical properties and time scales of the postsynaptic cell. Band-pass filters (BPFs) result from a combination of low-pass filters (LPFs) and high-pass filters (HPFs) operating at the same or different levels of organization. PSP BPFs can be inherited from the synaptic level (STP-mediated BPFs) or they can be generated across levels of organization due to the interaction between (i) a synaptic LPF and the PSP summation-mediated HPF (PSP peaks), and (ii) a synaptic HPF and the PSP summation-mediated LPF (PSP amplitude). These types of BPFs persist in response to more realistic presynaptic spike trains: jittered (randomly perturbed) periodic spike trains and Poisson-distributed spike trains. The response variability is frequency-dependent and is controlled by STP in a non-monotonic frequency manner. The results and lessons learned from the investigation of this basic network motif are a necessary step for the construction of a framework to analyze the mechanisms of generation of neuronal filters in networks with more complex architectures and a variety of interacting cellular, synaptic and plasticity time scales.

**Supplementary Information:**

The online version contains supplementary material available at 10.1007/s10827-025-00908-3.

## Introduction

**Fig. 1:**
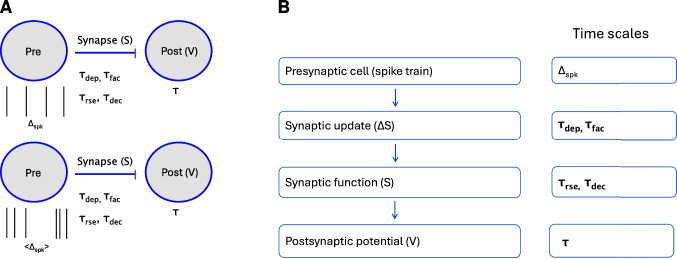
**Network motif: Feedforward excitation in the presence of short-term synaptic plasticity.** **A.** The presynaptic cell (Pre) is modeled as a spike train either periodic (period $\Delta _{spk}$, top), jittered-periodic or Poisson distributed (mean interspike interval $<\Delta _{spk}>$, bottom). The postsynaptic cell (Post, membrane potential $V$) is modeled as a passive cell (capacitive and leak currents) with a membrane time constant $\tau$. The excitatory synaptic variable ($S$) raises and decays with time constants $\tau _{rse}$ and $\tau _{dec}$, respectively. The synaptic depression and facilitation time constants are $\tau _{dep}$ and $\tau _{fac}$, respectively. **B.** We divide the study of the generation of postsynaptic membrane potential (PSP) filters in response presynaptic inputs within a range of (input) spiking frequencies (periodic or randomly-distributed) in three steps: (i) The $\Delta S$-filters generated in response to the presynaptic spike trains are shaped by the synaptic depression and facilitation time constants $\tau _{dep}$ and $\tau _{fac}$, respectively (e.g., Fig. 3, left columns), (ii) The synaptic $S$-filters generated as the result of the $\Delta S$-filters are further shaped by the synaptic rise and decay times $\tau _{rse}$ and $\tau _{dec}$, respectively (e.g., Fig. 3, middle columns), and (iii) the PSP filters generated in response to the $S$-filters are further shaped by the postsynaptic membrane potential time constant $\tau$ (e.g., Fig. 3, right columns)

**Fig. 2:**
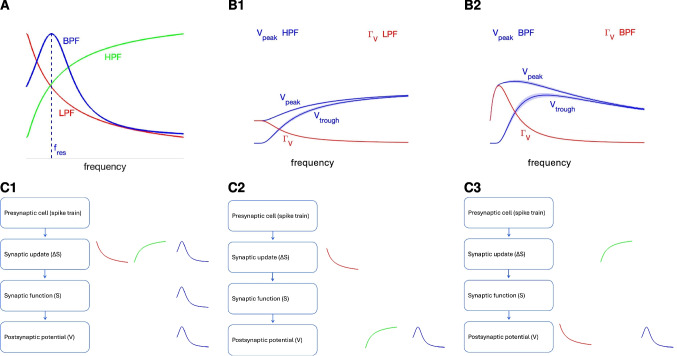
**Postsynaptic potential (PSP) Peak (**$\varvec{V_{peak}}$**) and amplitude (**$\varvec{\Gamma _V}$**) filters can be generated within and across levels of organization and communicated across levels of organization.** **A.** Band-pass filters (BPFs, blue) can be generated by the interplay of low-pass filters (LPFs, red) and high-pass filters (HPFs, green) within and across levels of neuronal organization and are shaped by the participating time constants (Fig. 1). The PSP resonant frequency $f_{res}$ is the BPF’s peak frequency. **B.** The PSP ($V_{peak}$) and PSP amplitude ($\Gamma _V = V_{peak} - V_{trough}$) profiles (curves of these metrics as a function of the spiking input frequency) can have the different (HPF and LPF, left) or the same (both BPFs, right) filtering properties. **C.** Schematic diagrams of PSP BPFs ($V_{peak}$ or $\Gamma _V$ ) generated within and across levels of organization. **C1.** A PSP BPF can be generated by the interplay of a LPF and a HPF at the synaptic update level. The $\Delta S$ BPF is inherited to the PSP level. **C2.** A PSP BPF can generated by the interplay of a synaptic update LPF and a PSP HPF. **C3.** A PSP BPF can generated by the interplay of a synaptic update HPF and a PSP LPF. **C1, C2, C3.** The synaptic update BFPs, LPFs and HPFs can experience modulations as they are communicated across levels

Neuronal filters allow neuronal systems to select certain information or enhance the communication of specific information components over others (Hutcheon & Yarom, 2000; Tsodyks et al., 2000; Izhikevich et al., 2003; Stark et al., 2022; Laudansky et al., 2014; Akam & Kullmann, 2012). Owing to this, neuronal filters play important roles in neuronal information processing, rhythm generation and brain computations (Maass, 2016; Beiran & Ostojic, 2019; Fortune & Rose, 2001; Klyachko & Stevens, 2006; Thomson, 2003; Akam & Kullmann, 2010; Stark et al., 2022; Blankenburg et al., 2015; Rosenbaum et al., 2012; Brunel et al., 2001; Buonomano & Maass, 2009; Izhikevich et al., 2003; Lisman, 1997; Sherfey et al., 2018). Band-pass frequency-filters are associated to the notion of neuronal resonance (Hutcheon & Yarom, 2000; Stark et al., 2013, 2022), which has been observed at various levels of neuronal organization, from the subthreshold membrane potential to the network levels (Stark et al., 2022) (Fig. 1). Neuronal resonance refers to the ability of a neuronal system to exhibit a maximal response (e.g., subthreshold membrane potential, postsynaptic potential, firing rate) to periodic inputs at a preferred (resonant), non-zero frequency band (Fig. 2-A). At the cellular level, in response to oscillatory (sinusoidal) inputs, frequency-filters reflect the time scales of the participating currents (Hutcheon & Yarom, 2000; Puil et al., 1986, 1987). At the synaptic level, in response to presynaptic spike inputs, frequency-filters (Moreno-Bote & Parga, 2004) reflect the synaptic rise and decay times. The observed postsynaptic responses reflect the combination of these and the time scales of the postsynaptic cell’s participating currents (Drover et al., 2007), which may give rise to additional filtering components resulting from the summation phenomenon (George et al., 2011). In synaptic pairs with more complex synaptic dynamics, frequency-filters also reflect the time scales associated with synaptic short-term plasticity (STP; depression and facilitation) and may give rise to band-pass filters (BPFs) at the synaptic and postsynaptic levels (Zucker, 1989; Zucker & Regehr, 2002; Stevens & Wang, 1995; Tsodyks et al., 2000; Izhikevich et al., 2003; Drover et al., 2007; Stark et al., 2022). How the concerted activity of this multiplicity of time scales shape neuronal filters is poorly understood.

STP refers to the changes of the efficacy of synaptic transmission (synaptic conductance strength) in response to presynaptic spike trains (as the number spikes increases) with time scales ranging in the order of hundreds of milliseconds to seconds (Zucker, 1989; Zucker & Regehr, 2002; Stevens & Wang, 1995). STP consists of the combination of two opposing processes with characteristic time scales: synaptic short-term depression (efficacy decrease; STD) and facilitation (efficacy increase; STF). STP has been investigated in both vertebrates and invertebrates. It has been shown to be involved in a number of brain functions, including information filtering (temporal and frequency-dependent) (Dittman et al., 2000; Silberberg et al., 2004; Markram et al., 1998a; Fortune & Rose, 2000, 2001, 1997a, b, 2002; Thomson, 2003; Goldman et al., 2002; Mejias & Torres, 2008; Bourjaily & Miller, 2012; Klyachko & Stevens, 2006; George et al., 2011; Lewis & Maler, 2002; Kandaswamy et al., 2010; Varela et al., 1997; Chance et al., 1998; Zador & Dobrunz, 1997; Lisman, 1997; Izhikevich et al., 2003; Buonomano, 2000; Zucker & Regehr, 2002; Pouille & Scanziani, 2004; Gabernet et al., 2005), adaptive filtering (Klyachko & Stevens, 2006) and related phenomena (e.g., burst detection) (Goldman et al., 2002; Izhikevich et al., 2003; Mondal et al., 2022; Reyes et al., 1998; Markram et al., 1998; Tsodyks et al., 1998), temporal coding and information processing (Tsodyks & Markram, 1996, 1997; Goldman et al., 2002; Mejias & Torres, 2008; Rotman et al., 2011; Tauffer & Kumar, 2021), information flow (Fuhrmann et al., 2004; Maass & Zador, 1999; Zador & Dobrunz, 1997) (given the presynaptic history-dependent nature of STP), gain control (Abbott et al., 1997; Tsodyks & Wu, 2013; Abbott & Regehr, 2004), the modulation of network responses to external inputs (Loebel & Tsodyks, 2002; Barak & Tsodyks, 2007), the prolongation of neural responses to transient inputs (Karmarkar & Buonomano, 2007; Buonomano & Maass, 2009; Mongillo et al., 2015), direction selectivity (Carver et al., 2008), vision (e.g., microsacades) (Yuan et al., 2013), sound localization and hearing (Cook et al., 2003; Hennig et al., 2008), the generation of cortical up and down states (Holcman & Tsodyks, 2006), attractor dynamics (Amari, 1977; Tsodyks & Wu, 2013), navigation (e.g., place field sensing) (Klyachko & Stevens, 2006; Kandaswamy et al., 2010), working memory (Barak et al., 2008; Mongillo et al., 2015), decision making (Deco et al., 2010) and neuronal computation (Destexhe & Marder, 2004; Abbott & Regehr, 2004; Maass & Zador, 1999; Mejias & Torres, 2009; Deng & Klyachko, 2011; Maass, 2016).

Neuronal resonance has been investigated both experimentally and theoretically at various levels of organization ranging from the cellular to the synaptic to the network levels (Puil et al., 1986, 1987; Wang, 2010; Pike et al., 2000; Zemankovics et al., 2010; Richardson et al., 2003; Rotstein & Nadim, 2014; Rotstein, 2015, 2017; Markram et al., 1998; Drover et al., 2007; Izhikevich et al., 2003; Akam & Kullmann, 2010; Kang et al., 2010; Vierling-Claassen et al., 2010; Ledoux & Brunel, 2011; Veltz & Sejnowski, 2015; Sherfey et al., 2018; Stark et al., 2013, 2022). In recent work (Stark et al., 2022), we demonstrated that BPFs can be inherited from lower (e.g., cellular) to higher (e.g., network) levels of organization or can be created independently at various levels of organization by the interplay of low-pass filters (LPFs) and high-pass filters (HPFs) belonging to the same or different levels (e.g., Fig. 2). However, much remains to be understood about the biophysical and dynamic mechanisms of generation of BPFs beyond the single cell level. It is unclear how neuronal filters are shaped by the time scales of the participating building blocks at each level of organization (Fig. 1). It is also unclear how the neuronal filtering properties are communicated across levels of organization, how they are modulated by the time scales and other biophysical properties as they transition from one level to another, and how they interact within and across levels of organization.

In this paper we systematically analyze and develop these ideas in a feedforward network motif (Fig. 1-A), which is arguably the elementary neuronal network processing unit and involves a multiplicity of interacting time scales. The building blocks consist of a presynaptic spike-train (characteristic period $\Delta _{spk}$), a postsynaptic passive cell (time constant $\tau$), an excitatory (AMPA) chemical synapse (rise and decay time constants $\tau _{rse}$ and $\tau _{dec}$, respectively), and STP (depression and facilitation constants $\tau _{dep}$ and $\tau _{fac}$, respectively). We use mathematical modeling, numerical simulations and analytical calculations on simplified models to analyze the PSP response to presynaptic spike trains across three levels of organization: (i) the so-called synaptic update level, which is the target of the synaptic variable and is affected by STP, (ii) the synaptic variable level, and (iii) the PSP level.

We first use periodic spike-trains with frequencies $f_{spk}$ (period $\Delta _{spk}$) within some range to characterize the different types of filters (BPFs, LPFs and HPFs; Fig. 2-A) and to understand the mechanisms by which they are generated. We then use Poisson-distributed spike trains with mean rates within the same range (as the periodic spike trains) to extend our investigation and findings to more realistic scenarios. To link between the purely deterministic and purely stochastic approaches, we use the so-called jitter-periodic inputs (Mondal et al., 2022). The use of randomly distributed spikes also allow us to explore how the variability of the filtering properties is controlled by STP and other biophysical properties and time scales of the participating building blocks.

We compute the stationary, frequency-dependent PSP response to presynaptic spike trains. The temporal (transient) responses and the associated temporal filters in the same feedforward network were thoroughly investigated in Mondal et al. (2022). We primarily focus on two metrics (Fig. 2-B) that give rise to two types of profiles (curves of the corresponding metrics as a function of the spike-train input frequency or firing rate): (i) peak profiles ($V_{peak}$), (ii) peak-to-trough amplitude profiles ($\Gamma _V$). For PSP filters, $\Gamma _V$ is analogous to the impedance amplitude profile for the computation of the standard subthreshold filters (in response to direct activation of sinusoidal inputs), while $V_{peak}$ is relevant for the communication of the PSP filtering effects to the spiking regime and therefore to other building blocks in larger networks. To a lesser extent, we consider a third metrics, the phase profiles, which extend the notion of impedance phase to the PSP responses to presynaptic inputs.

The effects of STP are present at the synaptic update level (Fig. 1-B, box 2) and are communicated to the synaptic level where they interact with the synaptic time scales (Fig. 1-B, box 3). The PSP filters result from the interaction between these variables and time scales and the biophysical properties and time scales of the postsynaptic cell (Fig. 1-B, box 4). Band-pass filters (BPFs) result from a combination of low-pass filters (LPFs) and high-pass filters (HPFs) (Fig. 2-A) operating at the same (Fig. 2-C1) or different (Fig. 2-C2 and -C3) levels of organization. PSP BPFs can be inherited from the synaptic update level (STP-mediated BPFs; Fig. 2-C1) or they can be generated across levels of organization due to the interaction between (i) a synaptic LPF and the PSP summation-mediated HPF filter (PSP peaks; Fig. 2-C2), and (ii) a synaptic HPF and the PSP summation-mediated LPF (PSP amplitude; Fig. 2-C3).

In spite of its simplicity, the feedforward network motif we use is dynamically rich. First, passive cells produce subthreshold (membrane potential) LPFs in response to sinusoidal inputs currents (the voltage amplitude response as a function of the input frequency is monotonically decreasing), but they may produce subthreshold HPFs in response to presynaptic periodic spike-train inputs due to the effects of summation. Second, synaptic depression and facilitation produce LPFs and HPFs, respectively in response to presynaptic spike inputs (Izhikevich et al., 2003). Third, the cellular filtering properties are primarily the result of electric circuit effects, while STP filtering properties are primarily the result of history-dependent processes. Finally, filters may be generated and interact within and across levels of organization (Stark et al., 2022; Mondal et al., 2022) as the result of the interplay of the biophysical building blocks that give rise to them. The lessons learned from this study will serve to construct a framework to analyze the mechanisms of generation of neuronal filters in networks with more complex cells and architecture.

The outline of the paper is as follows. In Section 2 we present the models and tools we use in this paper. We first describe the biophysically-plausible model for the postsynaptic response of passive cells to presynaptic spikes in the presence of STP. Then, we develop several reduced models for the synaptic dynamics, PSP dynamics and the dynamics of the membrane potential of passive cells in response to presynaptic spikes. These simplified models are amenable to analytical calculations, which allow us to understand how the interplay of the participating STP, synaptic and membrane time constants shape the PSP filters.

In Section 3.1 we analyze the generation of the synaptic update filters, which feed into the synaptic filters (and are the target of the synaptic variable $S$), in the presence of STP and the dependence of their characteristic frequencies and attributes (e.g., resonance frequency) on the single event STP time constants $\tau _{dep}$ and $\tau _{fac}$. In Section 3.2 we analyze the inherited and cross-level mechanisms of generation of synaptic ) STP-mediated BPFs ($\bar{S}$ in a reduced model for synaptic dynamics. In Section 3.3 we analyze the inherited and cross-level mechanisms of generation of PSP BPFs in a reduced models of PSP dynamics in the presence of STP that incorporates the effects of summation (SUM) and SUM-mediated HPFs.

Armed with the insight and intuition gained from the previous sections, in Sections 3.4 and 3.5 we begin our analysis of the interplay of STP- and SUM-mediated filters in the postsynaptic cell, explain the mechanisms of generation of SUM-mediated HPFs in passive cells and compare the mechanisms by which they are generated with the mechanisms of generation of LPFs in response to direct sinusoidal inputs. In Section 3.6 we focus on the inherited and cross-level mechanisms of generation of PSP peak and amplitude BPFs (PSP resonance). These tools are used in the following section to understand the mechanisms underlying the generation of the different types of BPFs. In Section 3.7 we show that the interplay of STD-mediated LPFs and PSP SUM-mediated HPFs produce $V_{peak}$ BPFs and $\Gamma _V$ LPFs. In Section 3.8 we show that the Interplay of STF-mediated HPFs and PSP SUM-mediated filters produce $V_{peak}$ LPFs and $\Gamma _V$ BPFs. In Section 3.9 we show that interplay of STD-mediated LPFs, STF-mediated HPFs and PSP SUM-mediated filters produces $V_{peak}$ and $\Gamma _V$ BPFs. In all cases, the participating filters are modulated by the time constants not directly involved in their generation.

The filters investigated in the previous sections are deterministic (in response to periodic presynaptic spike trains). In Sections 3.10 and 3.11 we extend our investigation to include randomly-distributed presynaptic spike trains. In Section 3.10 we show that the STP-mediated PSP $V_{peak}$ and $\Gamma _V$ BPFs persist with modulations in response to randomly-distributed spike trains both jittered-periodic and Poisson-distributed. In Section 3.11 we demonstrate that STP controls the variability of PSP $V_{peak}$ and $\Gamma _V$ BPFs in response to jittered-periodic and Poisson distributed spike trains in a frequency-dependent manner. We discuss our results and their implications in Section 4.

The development of the approximate solutions to simplified models is presented in the Appendix A. Tables of acronyms (Table 1), metrics (Table 2), model variables (Table 3, top) and model parameters (Table 3, bottom) are provided in the Appendix B.

## Methods

### Models

#### Postsynaptic cell

The current-balance equation for the post-synaptic cell is given by

1 $$\begin{aligned} C\, \frac{dV}{dt} = -G_L\, (V-E_L) + I_{app}-I_{syn} \end{aligned}$$

where $t$ is time, $V$ represents the voltage, $C$ is the specific capacitance, $G_L$ is the leak conductance, $I_{app}$ is the tonic (DC) current, and $I_{syn}$ is an excitatory synaptic current of the form

2 $$\begin{aligned} I_{syn} = G_{syn}\, S\, (V - E_{syn}). \end{aligned}$$

In Eq. (2), $G_{syn}$ is the maximal synaptic conductance, $E_{syn}$ is the reversal potential and $S$ is the synaptic variable.

We used the following units: ms for time, mV for membrane potential (voltage), $\mu$F/cm^2^ for capacitance, mS/cm^2^ for conductance, $\mu$A/cm^2^ for current and Hz for frequency.

#### Synaptic dynamics

The synaptic variables $S$ obey a kinetic equation of the form

3 $$\begin{aligned} \frac{dS}{dt} = N(V_{pre})\, \frac{ (\Delta S - S)}{\tau _{rse}} - \frac{S}{\tau _{dec}}, \end{aligned}$$

where $V_{pre}$ is the membrane potential of the presynaptic spike, $N(V)$ denotes the sigmoid function

4 $$\begin{aligned} N(V) = \frac{1 + \tanh (V/4)}{2} \end{aligned}$$

$\tau _{rse}$ and $\tau _{dec}$ are the rise and decay time constants respectively, and $\Delta S$ is a target value for $S$. For AMPA excitation (E-cells, $G_{syn} = G_{ex}$), we used $E_{syn} = E_{ex} = 0$, $\tau _{rse} = 0.1$ and $\tau _{dec} = 3.0$ (Borgers et al., 2012). In the absence of synaptic short-term dynamics (depression and facilitation), $\Delta S = 1$. Otherwise, $\Delta S$, interpreted as the magnitude $\Delta S$ of the synaptic release per presynaptic spike, is determined as described below (Sections 2.1.4 and 2.1.6).

We refer the reader to Dayan and Abbott (2001), Ermentrout and Terman (2010), and Miller (2018) for additional details on biophysical (conductance-based) models.

#### Presynaptic spike-trains

We model the spiking activity of the presynaptic cell as a spike train with presynaptic spike times $t_1, t_2, \ldots , t_N$. We consider three types of input spike-trains. Periodic inputs are characterized by the interspike interval (ISI) of length $\Delta _{spk}$ or, alternatively, by the spiking frequency (Hz)

5 $$\begin{aligned} f_{spk} = \frac{1000}{\Delta _{spk}}. \end{aligned}$$

Jittered-periodic presynaptic spike trains consist of perturbations of periodic presynaptic spiking patterns of the form

6 $$\begin{aligned} \Delta _{spk,n} = \Delta _{spk} + \delta _{spk,n} \end{aligned}$$

where $\Delta _{spk}$ is constant ($n$-independent) and $\delta _p = \{ \delta _{spk,n} \}_{n=1}^{N_{spk}}$ is a sequence of real numbers. We take $\delta _p$ to be normally distributed with zero mean and variance $\sigma ^2$. Poisson distributed (homogeneous) presynaptic spike trains are characterized by the mean spiking rate (and the associated exponential distribution of ISIs).

#### The DA (Dayan-Abbott) model for short-term dynamics: synaptic depression and facilitation

This phenomenological model is presented in Ermentrout and Terman (2010) and attributed to Dayan and Abbott (and collaborators). The magnitude $\Delta S$ of the synaptic release per presynaptic spike is assumed to be the product of the depression ($x$) and facilitation ($z$) variables

7 $$\begin{aligned} \Delta S = x^-\, z^+ \end{aligned}$$

where

8 $$\begin{aligned} \frac{dx}{dt} = \frac{x_{\infty }-x}{\tau _{dep}} - a_d\, x\, \delta (t - t_{spk}), \end{aligned}$$

and

9 $$\begin{aligned} \frac{dz}{dt} = \frac{z_{\infty }-z}{\tau _{fac}} + a_f\, (1 - z)\, \delta (t - t_{spk}). \end{aligned}$$

Each time a presynaptic spike arrives ($t = t_{spk}$), the depressing variable $x$ is decreased by an amount $a_d\, x$ (the release probability is reduced) and the facilitating variable $z$ is increased by an amount $a_f\, (1 - z )$ (the release probability is augmented). During the presynaptic ISIs both $x$ and $z$ decay exponentially to their saturation values $x_{\infty }$ and $z_{\infty }$ respectively. The rate at which this occurs is controlled by the parameters $\tau _{dep}$ and $\tau _{fac}$. Following others we use $x_{\infty } = 1$ and $z_{\infty } = 0$. The superscripts “±" in the variables $x$ and $z$ indicate that the update is carried out by taking the values of these variables prior (^-^) or after (^+^) the arrival of the presynaptic spike.

**Fig. 3:**
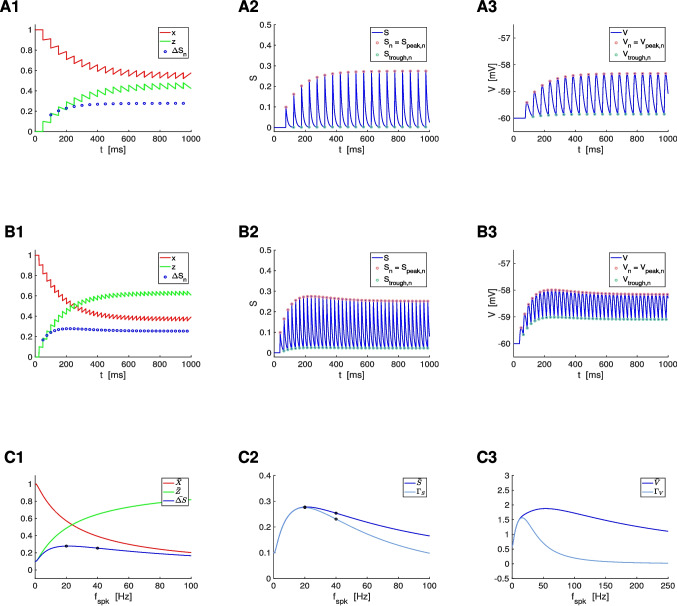
**Representative temporal patterns for the synaptic update **$\Delta S$**, the synaptic variable **$S$**, and the postsynaptic membrane potential **$V$ **in the presence of short-term dynamics (STD).** We used the model for the postsynaptic cell described by Eqs. (1)-(4) with STD described by the DA model (7)-(9), and periodic presynaptic spike trains with frequency $f_{spk}$ (see schematic Fig. 1, left). **Left column.** Short-term dynamics. The peak sequence $\Delta S_n$ ($n = 1, \ldots , N_{spk}$) is the synaptic update to the synaptic variable $S$ upon the arrival of each presynaptic spike, and results from the combined effect of the depression (x) and facilitation (z) variables. The stationary value of the $\Delta S_n$ sequences is referred to as $\bar{\Delta S}$. **Middle column.** Synaptic dynamics. The amplitude $\Gamma _S =\bar{S}_{peak,n}-\bar{S}_{trough,n}$, where $\bar{S} = \bar{S}_{peak}$ and $\bar{S}_{trough}$ are the stationary values of the sequences $S_{peak,n}$ and $S_{trough,n}$, respectively. **Right column.** Membrane potential dynamics. The amplitude $\Gamma _V = \bar{V}_{peak,n}-\bar{V}_{trough,n}$, where $\bar{V} = \bar{V}_{peak}$ and $\bar{V}_{trough}$ are the stationary values of the sequences $V_{peak,n}$ and $V_{trough,n}$, respectively. **A.** $f_{spk} = 20 Hz$. **B.** $f_{spk} = 40 Hz$. **C.** Frequency profiles of the stationary peaks $\bar{\Delta S}$ (left, blue), $\bar{S}$ (middle, blue) and $\bar{V}$ (right, blue) for the peak sequences $\Delta S_n$, $S_n$ and $V_n$, respectively, and stationary peak-to-trough amplitude profiles $\Gamma _S$ (middle, light blue) and $\Gamma _V$ (right, light blue) for $S$ and $V$, respectively. The black dots correspond to the presynaptic input frequencies in $A$ and $B$. We used the following additional parameter values: $a_d = 0.1$, $a_f = 0.1$, $x_{\infty } = 1$, $z_{\infty } = 0$, $\tau _{dep} = 400$, $\tau _{fac} = 400$, $\tau _{rse} = 0.1$, $\tau _{dec} = 10$, $C = 1$, $E_L = -60$, $G_L = 0.1$, $I_{app} = 0$, $G_{syn} = 0.025$, $E_{syn} = 0$

Figures 3-A1 and -B1 illustrates the $x$-, $z$-traces (curves of $x$ and $z$ as a function of time) in response to a periodic presynaptic input train for representative parameter values. The updated of the variable $S$ at the arrival of presynaptic spikes is given by $\Delta S = x^- z^+$. The sequence of peaks for $\Delta S$ is defined by $\Delta S_n = X_n Z_n$ where $X_n$ and $Z_n$ are the sequence of peaks for the variables $x$ and $z$, respectively (see also Mondal et al., 2022).

#### Response of the DA model to presynaptic inputs

##### Peak dynamics and temporal filters

By solving the differential equations (8)-(9) during the presynaptic ISIs and appropriately updating the solutions at $t = t_n$ (occurrence of each presynaptic spike), one arrives at the following recurrent formula for the peak sequences in terms of the model parameters

10 $$\begin{aligned} X_{n+1} = x_{\infty } + [\, (1 - a_d) X_n - x_{\infty }\, ]\, e^{-\Delta _{spk,n}/\tau _{dep}} \end{aligned}$$

and

11 $$\begin{aligned} Z_{n+1} = a_f + (1 - a_f)\, [\, z_{\infty } + (Z_n - z_{\infty }) e^{-\Delta _{spk,n}/\tau _{fac}}\ ] \end{aligned}$$

where $\{\Delta _{spk,n}\}_{n=1}^{Nspk}$ represents the lengths of the presynaptic ISIs.

The temporal filtering properties of the DA model in response to periodic and Poisson-distributed presynaptic inputs was studied in Mondal et al. (2022).

##### Steady-state frequency-dependent filters

For periodic inputs, $\Delta _{spk,n} = \Delta _{spk}$, independent of $n$, Eqs. (10)-(11) are linear 1D difference equations. Therefore both the sequences $X$ and $Z$ obey linear discrete dynamics (e.g., see Rotstein & Tabak, 2019), decaying to their steady state values

12 $$\begin{aligned} \bar{X} = \frac{(\, 1 - e^{-\Delta _{spk}/\tau _{dep}}\, )\, x_{\infty }}{1 - (1-a_d)\, e^{-\Delta _{spk}/\tau _{dep}}} \end{aligned}$$

and

13 $$\begin{aligned} \bar{Z} = \frac{(\, 1 - e^{-\Delta _{spk}/\tau _{fac}}\, )\, (1- a_f)\, z_{\infty } + a_f}{1 - (1-a_f)\, e^{-\Delta _{spk}/\tau _{fac}}}. \end{aligned}$$

For the reminder of this paper we use $x_{\infty } = 1$ and $z_{\infty } = 0$.

#### The MT (Markram-Tsodkys) model for short-term dynamics: synaptic depression and facilitation

This phenomenological model was introduced in Markram et al. (1998) as a simplification of earlier models (Tsodyks & Markram, 1997; Zucker, 1989; Magleby & Zengel, 1982). It is slightly more complex and widely used than the DA model described above (Hennig, 2013; Izhikevich et al., 2003). As for the DA model, the magnitude $\Delta S$ of the synaptic release per presynaptic spike is assumed to be the product of the depressing and facilitating variables

14 $$\begin{aligned} \Delta S = R^-\, u^+ \end{aligned}$$

where, in its more general formulation,

15 $$\begin{aligned} \frac{dR}{dt} = \frac{1-R}{\tau _{dep}} - R^- u^+\, \delta (t - t_{spk}), \end{aligned}$$

and

16 $$\begin{aligned} \frac{du}{dt} = \frac{\hat{U}-u}{\tau _{fac}} + U\, (1-u^-)\, \delta (t - t_{spk}). \end{aligned}$$

Each time a presynaptic spike arrives ($t = t_{spk}$), the depressing variable $R$ is decreased by $R^- u^+$ and the facilitating variable $u$ is increased by $U\, (1-u^-)$. As before, the superscripts “±" in the variables $R$ and $u$ indicate that the update is carried out by taking the values of these variables prior (^-^) or after (^+^) the arrival of the presynaptic spike. In contrast to the DA model, the update of the depression variable $R$ is affected by the value of the facilitation variable $u^+$. Simplified versions of this model include making $\hat{U} = 0$ (Markram et al., 1998; Markram & Tsodyks, 1996; Tsodyks et al., 1998, 2000; Markram et al., 1998a, b) and $\hat{U} = U$ (Izhikevich et al., 2003).

#### Response of the MT model to presynaptic inputs

##### Peak dynamics and temporal filters

By solving the differential equations (15)-(16) during the presynaptic ISIs and appropriately updating the solutions at $t = t_n$ (occurrence of each presynaptic spike), one arrives at the following recurrent formula for the peak sequences in terms of the model parameters

17 $$\begin{aligned} R_{n+1} = R_n (1-u_{n+1}) e^{-\Delta _{spk}/\tau _{dep}} + 1 - e^{-\Delta _{spk,n}/\tau _{dep}} \end{aligned}$$

and

18 $$\begin{array}{c}u_{n+1}=\widehat U+U-\widehat UU+u_n(1-U)e^{-\Delta_{spk,n}/\tau_{fac}}\\-\widehat U(1-U)e^{-\Delta_{spk,n}/\tau_{fac}}.\end{array}$$

The temporal filtering properties of the DA model in response to periodic presynaptic inputs was studied in Mondal et al. (2022).

##### Steady-state frequency-dependent filters

As before, for periodic presynaptic inputs $\Delta _{spk,n} = \Delta _{spk}$, independent of $n$, these equations represent a system of two 1D difference equations. The steady-state values are given by

19 $$\begin{aligned} \bar{R} = \frac{1 - e^{-\Delta _{spk}/\tau _{dep}}}{1 - (1-\bar{u})\, e^{-\Delta _{spk}/\tau _{dep}}} \end{aligned}$$

and

20 $$\begin{aligned} \bar{u} = \frac{\hat{U} + U - \hat{U} U - \hat{U}\, (1-U) e^{-\Delta _{spk}/\tau _{fac}}}{{1 - (1-U)\, e^{-\Delta _{spk}/\tau _{fac}}}}. \end{aligned}$$

For the reminder of this paper we will use $\hat{U} = 0$ and $U = 0.1$.

### Reduced models

#### A reduced model for synaptic dynamics in response to presynaptic spikes: The to-$\bar{\Delta S}$ synaptic update model

Here and in the next section we present two sets of reduced models under certain simplifying assumptions. These simplified models are amenable to analytical calculations, which allow us to understand how the interplay of the participating STP time constants ($\tau _{dep}$ and $\tau _{fac}$) and synaptic time constants ($\tau _{dec}$ and $\tau _{rse}$) shape the synaptic and postsynaptic responses to presynaptic inputs.

For the parameter values consistent with AMPA excitation, the synaptic rise time $\tau _{rse}$ is very fast as compared to the synaptic decay time $\tau _{dec}$ and other times scales present in the model. Therefore, as a first level of approximation one can reduce the dynamics of the synaptic variable $S$ in Eq. (3) by

21 $$\begin{aligned} \frac{dS}{dt} = -\frac{S}{\tau _{dec}} \, [+]\, \Delta S_n \, \delta (t - t_{spk,n}) \end{aligned}$$

where the sign $[+]$ indicates that each presynaptic spike (e.g., at time $t_n$) instantaneously raises $S$ “to" $\Delta S_n$, which depend on the combined dynamics of STD and STF. We refer to this model as the “to-$\Delta S$" model in contrast to the scenario where each presynaptic spike instantaneously raises $S$ “by" some value $\Delta S_n$, which is discussed in the next section.

For generality and to make the model more realistic, it is instructive to explore how the results obtained for the above simplified model are affected by the presence of non-zero synaptic rise times, while keeping the model simplified. To this end, we substitute the factor $N(V_{pre})$ in Eq. (3) by a square pulse for the duration of the spike and separate the rise and decay process. The extended to-$\Delta S$ model reads

22 $$\begin{array}{c}\frac{dS}{dt}=\widehat H(t_{spk,n},t_{spk,n}+T_{sw})\,\frac{\Delta S_n-S}{\tau_{rse}}\\-\widehat H(t_{spk,n}+T_{sw},t_{spk,n+1})\,\frac S{\tau_{dec}},\end{array}$$

where $T_{sw}$ is the spike width, $t_{spk,n+1} = t_{spk,n}+\Delta _{spk,n}$ and $\hat{H}(t_1,t_2)$ is a square pulse defined as the product of two Heaviside functions $H(t)$, $\hat{H}(t_1,t_2) = H(t-t_1)\, H(t_2-t)$. From the arrival of each spike and for the duration of this spike, $S$ evolves according the first term in Eq. (22), while for the remaining of the presynaptic period, $S$ evolves according to the second term in Eq. (22).

#### A reduced model for PSP dynamics in response to presynaptic spikes: The by-$\bar{{\Delta S}}$ synaptic update model and the summation phenomenon

The reduced formulation we present here combines the synaptic and postsynaptic potential (PSP) responses to presynaptic input into a single equation. In contrast to the reduced models presented in Section 2.2.1, each presynaptic spike raises the variable $S$ “by" some value $\Delta S_n$, which depends on the combined dynamics of STD and STF. This allow for the effect of summation to unfold.

In the models presented in here, the variable $S$ is interpreted as the PSP response. Previous work by other authors have considered the PSP response frequency profiles to presynaptic inputs in the presence of STP to be proportional to the $\bar{\Delta S}$ profiles (e.g., Markram et al., 1998; Markram et al., 1998b; Markram et al., 1998a, but see Drover et al., 2007), without separating the dynamics of the synaptic and postsynaptic process. While we show that this is not always the case (as for STP-mediated temporal filters (Mondal et al., 2022)), the use of the simplified models we present here are amenable to analytical calculations and allow to gain insight and intuition into the interplay of the STP and membrane time constants and the interaction between the STP-mediated $\bar{\Delta S}$ and summation filters in shaping the PSP filtering properties.

We first assume instantaneous rate. The “by-$\bar{\Delta S}$" model reads

23 $$\begin{aligned} \frac{dS}{dt} = -\frac{S}{\tau _{dec}} + \Delta S_n \, \delta (t - t_{spk,n}). \end{aligned}$$

The “$+$" sign indicates that each presynaptic spike instantaneously raises $S$ “by" $\Delta S_n$.

The extended by-$\Delta S$ model, which includes the dynamics of the rise process and rise constant $\tau _{rse}$ reads

24 $$\begin{array}{c}\frac{dS}{dt}=\widehat H(t_{spk,n},t_{spk,n}+T_{sw})\,\frac{{\widehat{\Delta S}}_n-S}{\tau_{rse}}\\-\widehat H(t_{spk,n}+T_{sw},t_{spk,n+1})\,\frac S{\tau_{dec}},\end{array}$$

where $\hat{\Delta S}_n$ is the sum of $\Delta S_n$ and the value of $S$ preceding the arrival of each presynaptic spike. The other components are as for the to-$\Delta S$ model (22).

#### A reduced model for the membrane potential response of passive cells to presynaptic spikes

In order to analyze the PSP peak and amplitude responses of passive cells to presynaptic spikes we derive an analytical approximation of the solution to the model (1)-(4). The process consists of creating a reduced, hybrid model by substituting the conductance-based synaptic current in $I_{syn}$ (2) by a presynaptic ISI-dependent current-based input where the synaptic input coefficient is updated every cycle to account for the changes in the driving force $V - E_{syn}$ across cycles.

The postsynaptic passive cell (1) is linear, but the conductance-based synaptic input in $I_{syn}$ (2) is multiplicative. Previous work showed that the subthreshold rhythmic properties of cells in response to conductance- and current-based synaptic inputs, where the driving force is substituted by a constant, may qualitatively differ (Fernandez & White, 2008; Pena & Rotstein, 2022). Therefore, a simply substitution of the product of the synaptic input and the driving force by the product of the synaptic input and a constant (a current-based synaptic input) will not produce good enough approximations to the responses of conductance-based synaptic inputs (e.g., Figs. S3 and S4). In the hybrid approach we develop here, we approximate $V- E_{syn}$ by a constant, which we update at the end of each presynaptic ISI to account for the changing value of $V - E_{syn}$ across cycles. This hybrid model is amenable to analytical calculations and therefore is an analytical tool to analyze how the participating time constants shape the PSP peak and amplitude filters.

We first approximate Eq. (1) as follows

25 $$\begin{aligned} \tau \, \frac{dV}{dt} = - V + \alpha _n\, S_a(t) \end{aligned}$$

for $$n = 1, \ldots , N$$ where

26 $$\begin{aligned} \tau = \frac{C}{G_L}, \hspace{1cm} \alpha = \frac{G_{syn}\, (E_{syn}-V_{eq})}{G_L} \hspace{.5cm} \text{ and } \hspace{.5cm} \alpha _n = \alpha \, (1-\sigma _n). \end{aligned}$$

The variable $V$ in Eq. (25) represents $V - V_{eq}$ in Eq. (1) and $V_{eq} = E_L + I_{app}/G_L$. The right hand second term in Eq. (25) is a current input approximation to the conductance input in the synaptic current Eq. (1). To account for this, we introduce a correction factor $1 - \sigma _n$ where $\sigma _n$ is updated at the beginning of each ISI proportionally to the distance between the PSP response and $E_{syn}$ and $\sigma _1 = 0$. The function $S_a(t)$ is an approximation to the variable $S$ whose dynamics are described by Eq. (3) with $S(0) = 0$, under the assumption of instantaneous rise to a value $\Delta S_n$ ($n=1, \ldots , N$) at the arrival of each presynaptic spike. The assumption $S(0) = 0$ implies that $S(t) = 0$ for $0 \le t \le t_1$.

For the duration of each presynaptic spike ($t_n< t < t_n + T_{sw}$), we approximate $S(t)$ by the synaptic update value $\Delta S_n$ (constant). For the remainder of the presynaptic interspike interval ($t_n +T_{sw} \le t < t_{n+1}$), $S(t)$ decreases according to the second term in Eq. (3). The approximate description of the synaptic term is given by

27 $$\begin{aligned} S_a(t) = \left\{ \begin{array}{llll} \sigma _s\, \Delta S_n & & & t_n< t< t_n + T_{sw} \\ \\ \sigma _s\, \Delta S_n\, e^{-(t-t_n-T_{sw})/\tau _{dec}} & & & t_n +T_{sw} \le t < t_{n+1} \\ \end{array} \right. \end{aligned}$$

for $n = 1, \ldots , N$. In our computations we take $T_{sw} = 1$ and $\sigma _s=\tau _{dec}$/$(\tau _{dec}+\tau _{rse})$ $(1-exp(-(\tau _{dec}+\tau _{rse})$/$(\tau _{dec} \tau _{rse})))$, which is the solution of Eq. (3) computed at $t = T_{sw} = 1$. For $\tau _{rse} = 0.1$ and $\tau _{dec} = 10$, $\sigma _s = 0.9901$, while for $\tau _{rse} = 0.1$ and $\tau _{dec} = 3$, $\sigma _s = 0.9677$. The approximation error is *τ*_*dec*_/(*τ*_*dec*_ + *τ*_*rse*_)Δ*Sτ*_*rse*_*τ*_*dec*_/(*τ*_*rse*_ + *τ*_*dec*_) -(1 + *τ*_*rse*_*τ*_*dec*_/(*τ*_*rse*_ + *τ*_*dec*_)) *exp*(-(*τ*_*dec*_ + *τ*_*rse*_)/(*τ*_*dec*_*τ*_*rse*_)). For $\tau _{dec} = 10$, the error is equal to 0.0098 ($0.098\, \Delta S$) and for $\tau _{dec} = 3$, the error is equal to 0.0094 ($0.094\, \Delta S$). However, we note that we consider the hybrid model to be a simplified model that captures the dynamics of the model (1)-(4) rather than a model that produces an accurate approximation to the solution of the model (1)-(4)

#### Postsynaptic potential (PSP) peak sequences for the reduced model

We present here the main ideas and results. The detailed calculations are provided in the Appendix A.

For each input frequency $f_{spk}$, we compute the PSP peak sequence $V_{peak,n}$ ($n=1, 2, \ldots$) until $|\, V_{peak,n} - V_{peak,n-1}\, | < \delta _{tol} = 0.0001$. This tolerance $\delta _{tol}$ is a conservative number that allows for the transient responses (temporal filters, see Mondal et al., 2022) to wear off. We approximate the stationary value of $\bar{V}_{peak}$ by $V_{peak,n}$ (the last value in the resulting vector), $V_{trough}$ by $V_{trough,n}$, $t_{V,peak}$ by the time at which $V_{peak,n}$ occurs, and we use the corresponding value of $t_{spk}$, $t_{spk,n}$ (immediately preceding $V_{peak,n}$) for the computation of the PSP phase response in Eq. (42).

The PSP sequences are given by

##### PSP peak sequences for $\tau _{dec} \ne \tau$

28 $$\begin{array}{c}V_{peak,n}=\frac{\alpha_n\tau_{dec}\triangle S_n}{\tau_{dec}-\tau}e^{T_{sw}/\tau_{dec}}\\e^{-(t_{peak,n}-t_n)/\tau_{dec}}\\+\left[\beta_n-\frac{\alpha_n\tau_{dec}\triangle S_n}{\tau_{dec}-\tau}\right]\\e^{T_{sw}/\tau}e^{-\left(t_{peak,n}-t_n\right)/\tau}\end{array}$$

where

29 $$\begin{aligned} t_{peak,n}&= t_n + \frac{\tau _{dec}\, \tau }{\tau _{dec}-\tau }\, \ln \left( -\frac{b_n\, \tau _{dec}}{a_n\, \tau } \right) , \nonumber \\ \hspace{0.75cm} a_n&= \frac{\alpha _n\, \tau _{dec}\, \Delta S_n}{\tau _{dec}-\tau }\, e^{T_{sw}/\tau _{dec}} \nonumber \\ \hspace{0.75cm} b_n&= \left[ \, \beta _n - \frac{\alpha _n\, \tau _{dec}\, \Delta S_n}{\tau _{dec}-\tau }\, \right] \, e^{T_{sw}/\tau }, \end{aligned}$$

30 $$\begin{array}{c}\alpha_n=\alpha\,(1-\sigma_n),\\and\;\sigma_{n+1}=\frac{\eta\,V_{peak,n}}{(E_{syn}-E_L)}with\;\sigma_1=0,\end{array}$$

31 $$\begin{aligned} \beta _n = \alpha _n\, \Delta S_n + (V_{o,n} - \alpha _n\, \Delta S_n)\, e^{-T_{sw}/\tau }, \end{aligned}$$

and

32 $$\begin{array}{c}V_{0,n+1}=\frac{\alpha_n\tau_{dec}\triangle S_n}{\tau_{dec}-\tau}e^{T_{sw}/\tau_{dec}}e^{-\triangle_{spk,n}/\tau_{dec}}+\left[\beta_n-\frac{\alpha_n\tau_{dec}\triangle S_n}{\tau_{dec}-\tau}\right]\\\begin{array}{cc}e^{T_{sw}/\tau}e^{-\triangle_{spk,n}/\tau}&\begin{array}{c}\begin{array}{cc}\mathrm{with}&V_{0,1}=0\end{array}\end{array}\end{array}\end{array}$$

Equation (28) is obtained from Eq. 81 in the Appendix A. Note that $V_{trough,n} = V_{0,n+1}$.

##### PSP peak sequences for $\tau _{dec} = \tau$

33 $$\begin{array}{c}V_{peak,n}=\frac{\alpha_n\triangle S_n}\tau e^{\left(t_n+t_{sw}\right)/\tau_{dec}}t_{peak,n}e^{-t_{peak,n}/\tau}\\+\left[\beta_n-\frac{\alpha_n\triangle S_n}\tau\left(t_n+T_{sw}\right)\right]\\e^{\left(t_n+T_{sw}\right)/\tau_{dec}}e^{-t_{peak,n}/\tau}\end{array}$$

where

34 $$\begin{aligned} t_{peak,n}&= t_n + \tau - \frac{b_n}{a_n} \hspace{0.75cm} a_n = \frac{\alpha _n\, \Delta S_n}{\tau }\, e^{T_{sw}/\tau _{dec}} \hspace{0.70cm} \text{ and } \nonumber \\ \hspace{0.75cm} b_n&= \left[ \, \beta _n - \frac{\alpha _n\, \Delta S_n}{\tau }\, T_{sw}\, \right] \, e^{T_{sw}/\tau _{dec}}. \end{aligned}$$

35 $$\begin{aligned} V_{0,n+1}&= \frac{\alpha _n\, \Delta S_n}{\tau }\, e^{T_{sw}/\tau _{dec}}\, t_{n+1}\, e^{-\Delta _{spk,n}/\tau } \nonumber \\&\quad + \left[ \, \beta _n - \frac{\alpha _n\, \Delta S_n}{\tau }\, (t_n+T_{sw})\, \right] \, e^{T_{sw}/\tau _{dec}}\, e^{-\Delta _{spk,n}/\tau }. \end{aligned}$$

and $\alpha _n$, $\sigma _n$ and $\beta _n$ are given by Eqs. (30) and (31). Equation (88) is obtained from Eq. (81) in the Appendix A. In both cases we use $\eta = 1$. Note that $V_{trough,n} = V_{0,n+1}$.

Figures S1 and S2 compare the numerical solutions to the model (1)-(4) and the analytical approximation using Eqs. (25)-(27) together with Eqs. (78) and (81) in the Appendix A for representative parameter values. The analytical approximation tracks the numerical solution with relatively high accuracy. Figures S3 and S4 show the error between the numerical and analytical approximations to the stationary peaks ($V_{peak}$), troughs ($V_{trough}$) and peak times ($t_{peak}$) of the membrane potential responses of passive cells to presynaptic spikes for representative parameter values. For $V_{peak}$ and $V_{trough}$ (left and middle columns), the error is significantly higher for $\eta = 0$ (green curves) than for values of $\eta = 1$ (blue curves) or around this value (red and light blue curves). Setting $\eta = 0$ is equivalent to the current-based synaptic input approximation, while setting $\eta = 1$ (or around this value), corrects for the driving force, which varies as $V$ varies. In contrast, the error for $t_{peak}$ (right columns) is largely independent of $\eta$.

### Membrane potential response to presynaptic inputs and to direct activation of sinusoidal inputs

The filtering properties of passive cells (and of neurons in general) depend on whether they are directly activated by sinusoidal inputs ($I_{in}$) or they are indirectly activated by periodic presynaptic inputs ($I_{syn}$) within the same frequency range. We distinguish between the cell’s membrane potential (peak-to-trough) amplitude and peak response profiles (curves of these quantities as a function of the input frequency $f$; Fig. 2-B). For cells with linear subthreshold dynamics receiving direct sinusoidal current activation, these two metrics coincide. (For certain types of cells with nonlinear subthreshold dynamics, they are good approximations of each other (Rotstein, 2015, 2017; Turnquist & Rotstein, 2018; Pena et al., 2019).) However, for presynaptic activation of postsynaptic cells, the PSP (peak-to-trough) amplitude and peak profiles do not generally coincide (Pena & Rotstein, 2022) (Fig. 2-B).

#### Impedance and amplitude profiles: response to direct activation of sinusoidal input currents

The impedance of a neuronal system receiving an input current $I_{in}(t)$ is defined as the ratio of the output (voltage) $V_{out}(t)$ and input (current) Fourier transforms

36 $$\begin{aligned} \varvec{Z}(f) = \frac{\mathcal{F}[V_{out}(t)](f)}{\mathcal{F}[I_{in}(t)](f)} \end{aligned}$$

where $f$ is frequency. The impedance $\varvec{Z}(f)$ is a complex quantity with amplitude $Z(f)$ and phase $\Phi _Z$. For nonlinear systems, we use the Fast Fourier Transform algorithm (FFT) to compute these quantities.

#### Impedance and phase profiles for the passive cell

The steady-state voltage response of a linear system receiving sinusoidal input currents of the form

37 $$\begin{aligned} I_{in}(t) = A_{in}\, sin(\omega \, t) \ \ \ \ \ \ \ \ \ \ \text{ with } \ \ \ \ \ \ \ \ \ \ \omega = \frac{2 \pi f}{1000}, \end{aligned}$$

where $f$ has units of Hz, is given by

38 $$\begin{aligned} V_{out}(t) = A_{in}\, Z(f)\, \sin {(\omega \, t - \Phi _Z(f))} \end{aligned}$$

where $Z(f)$ is the impedance amplitude and $\Phi _Z(f)$ is the phase offset (time difference between the peaks of the input current and the output voltage normalized by $2\, \pi$).

For a passive cell of the form (1), standard calculations show that

39 $$\begin{aligned} Z(\omega ) = \frac{\tau }{C}\, \frac{1}{\sqrt{1+\tau ^2\, \omega ^2}} \end{aligned}$$

and

40 $$\begin{aligned} \Phi _Z(\omega ) = \tan ^{-1} (\tau \, \omega ). \end{aligned}$$

The passive cell is a low-pass filter since the $Z(f)$ is monotonically decreasing. The response of the passive cell is delayed for all input frequencies and this delay increases with the input frequency since $\Phi _Z(\omega )$ is monotonically increasing.

#### PSP Peak, amplitude and phase profiles: response to presynaptic inputs

We characterize the PSP response to presynaptic inputs by using three metrics: the peak profiles, the peak-to-trough amplitude profiles and the phase profiles.

The PSP peak profiles $V_{peak}(f_{spk})$ are defined as the curves of the steady-state peak values of $V$ as a function of the presynaptic spike input frequency $f_{spk}$ (Fig. 2-B, blue). The PSP peak-to-trough amplitude profiles $\Gamma _V(f_{spk})$ are defined as

41 $$\begin{aligned} \Gamma _V(f_{spk}) = V_{peak}(f_{spk}) - V_{trough}(f_{spk}) \end{aligned}$$

where $V_{trough}$ are the PSP trough profiles (curves of the steady-state trough values of $V$ as a function of $f_{spk}$) (Fig. 2-B, red).

The PSP phase profiles $\Phi _V$ are defined as

42 $$\begin{aligned} \Phi _V = \frac{t_{peak,V}-t_{spk}}{\Delta _{spk}/\pi }, \end{aligned}$$

expressed in radians, where $t_{peak,V}$ is the $V_{peak}$ time and $t_{spk}$ is the presynaptic spike time immediately preceding the occurrence of this peak (Fig. 9-A2, blue).

The $\Gamma _V$ and $\Phi _V$ profiles are analogous metrics to the $Z$ and $\Phi _Z$ profiles. The $V_{peak}$ profiles capture the postsynaptic cell’s ability to preferentially produce spikes within certain presynaptic frequency ranges, and is therefore relevant for the frequency-dependent communication of information to the postsynaptic spiking regime.

### Numerical simulations

The numerical solutions were computed using the modified Euler method (Runge-Kutta, order 2) (Burden & Faires, 1980) with a time step $\Delta t = 0.01$ ms (or smaller values of $\Delta t$ when necessary) in MATLAB (The Mathworks, Natick, MA). The codes are available at https://github.com/BioDatanamics-Lab/Frequency_Filters_STP_21_06.

## Results

The question we ask in this paper is how the postsynaptic cell’s membrane potential frequency-filters (or -profiles) (curves of the appropriate metrics for each level of organization as a function of the input frequency $f_{spk}$; e.g., Fig. 3-C), depend on the properties of the participating building blocks: the presynaptic spike trains, the synaptic rise and decay dynamics, synaptic short-term plasticity (STP) and the intrinsic properties of the postsynaptic cells (Fig. 1-A).

Specifically, we conduct a systematic study of the steady-state postsynaptic membrane potential (PSP) response to periodic presynaptic inputs over a range of frequencies $f_{spk} = 1000 / \Delta _{spk}$ (Fig. 1-A, top) that capture the PSP filtering properties. We then extend our study to include jittered periodic inputs over a range of mean frequency $f_{spk} = 1000 / \Delta _{spk}$ and Poisson-distributed presynaptic inputs over a range of mean rates $r_{spk} = 1000 / <\Delta _{spk}>$ (Fig. 1-A, bottom).

We divide our study in three steps (Fig. 1-B): (i) the response profiles of the synaptic update $\Delta S$ to the presynaptic spike trains, (ii) the response profiles of the synaptic variable $S$ to $\Delta S$, and (iii) the response profiles of the postsynaptic membrane potential $V$ to $S$. Synaptic short-term plasticity (STP) operates at the $\Delta S$ level. The interaction between depression and facilitation (time constants $\tau _{dep}$ and $\tau _{fac}$, respectively) creates the synaptic update sequences $\Delta S_n = X_n Z_n$ (Fig. 3, left column, blue dots) where $X_n$ and $Z_n$ are the sequence of peaks for the depression and facilitation variables $x$ and $z$, respectively. These sequences are the target for the synaptic variables $S$ during the rise phase after the arrival of each presynaptic spike. In the absence of STP, $\Delta S_n$ is constant (typically set up to one). The interplay of $\Delta S_n$ and the synaptic dynamics (rise and decay time constants $\tau _{rse}$ and $\tau _{dec}$, respectively) creates the response synaptic ($S$) patterns (Fig. 3, middle column). The synaptic variable $S$ is the input to the current-balance equation (1) where the synaptic patterns interact with the postsynaptic biophysical membrane time constant $\tau$ to generate the postsynaptic ($V$) response patterns (Fig. 3, right column).

Here we focus on the frequency filtering properties of the steady-state responses for $\Delta S_n$, $S$ and $V$. We characterize them by using the $\bar{\Delta S}$, $\bar{S}$ and $\bar{V}$ peak profiles (Fig. 3-C, blue), defined as the curves of the stationary peaks for the corresponding quantities as a function of the input frequency $f_{spk}$, and the stationary peak-to-trough amplitude profiles (Fig. 3-C, light blue), consisting of the peak-to-trough amplitude curves as a function of $f_{spk}$, for the latter two quantities.

The temporal filtering properties (transient responses to spike-spike trains) of these feedforward networks were systematically investigated in Mondal et al. (2022).

### $\bar{\Delta } S$ band-pass filters: interplay of low-pass (depression) and high-pass (facilitation) filters

**Fig. 4:**
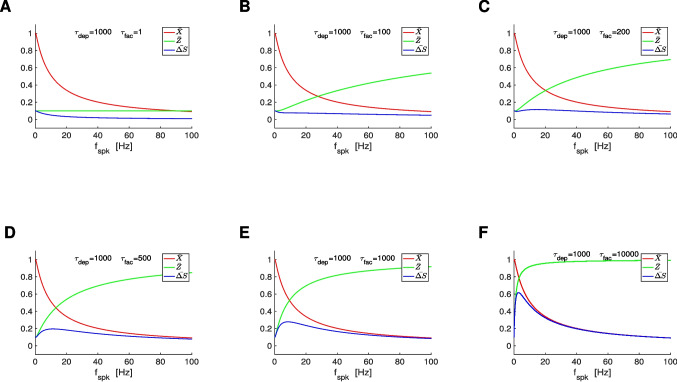
$\bar{\Delta S}$ **filters in response to periodic presynaptic spike inputs (frequency **$f_{spk}$**) for the DA model: representative examples.** We used Eqs. (12) and (13). **A.** $\tau _{fac} = 1$. **B.** $\tau _{fac} = 100$. **C.** $\tau _{fac} = 200$. **D.** $\tau _{fac} = 500$. **E.** $\tau _{fac} = 1000$. **F.** $\tau _{fac} = 10000$. We used the following additional parameter values: $a_d = 0.1$, $a_f = 0.1$, $x_{\infty } = 1$, $z_{\infty } = 0$ and $\tau _{dep} = 1000$

From Eqs. (12), (13) and (5), $\bar{X}$ is monotonically decreasing (low-pass filter; LPF), transitioning from $\bar{X} = 1$ ($f_{spk}=0$) to $\bar{X} = 0$ ( $f_{spk} \rightarrow \infty$), and $\bar{Z}$ is monotonically increasing (high-pass filter; HPF), transitioning from $\bar{Z} = a_f$ ($f_{spk}=0$) to $\bar{Z} = 1$ ($f_{spk} \rightarrow \infty$). This is illustrated in Fig. 4 (red and green) for representative parameter values. The interplay of depression and facilitation produces $\bar{\Delta S} = \bar{X} \bar{Z}$ LPFs, BPFs or more complex patterns depending on the relative values of $\tau _{dep}$ and $\tau _{fac}$ (Fig. 4, blue), for fixed realistic values of the remaining parameters.

#### BPFs: A trade-off between depression- and facilitation-dominated regimes

To simplify the mechanistic analysis, we define

43 $$\begin{aligned} \hat{\Delta }_{spk} = \frac{\Delta _{spk}}{\tau _{dep}} \hspace{1.5cm} \text{ and } \hspace{1.5cm} \eta _{stp} = \frac{\tau _{dep}}{\tau _{fac}}. \end{aligned}$$

Substitution into Eqs. (12) and (13) (with $x_{\infty } = 1$ and $z_{\infty } = 0$) yields

44 $$\begin{array}{ccc}\widehat X=\frac{1-e^{-{\widehat\Delta}_{spk}}\,}{1-(1-a_d)\,e^{-{\widehat\Delta}_{spk}}}{}&\mathrm{and}&\widehat Z=\frac{a_f}{1-(1-a_f)\,e^{-{\widehat\Delta}_{spk}\,\eta_{stp}}}.\end{array}$$

This rescaling allows us to investigate the mechanisms of generation of $\bar{\Delta S}$ band-pass filters (BPFs) as a function of a single parameter ($\eta _{stp}$) describing the relative magnitudes of the single event time constants $\tau _{dep}$ and $\tau _{fac}$. Specifically, $\hat{X}$ decreases with increasing values of $\hat{\Delta }_{spk}$ in a $\eta _{stp}$-independent manner, while the rate of increase of $\hat{Z}$ with $\hat{\Delta }_{spk}$ depends on the ratio $\eta _{stp}$ of $\tau _{dep}$ and $\tau _{fac}$. The shapes of the $\bar{\Delta S}$ filters for all values of $\tau _{dep}$ and $\tau _{fac}$ unfold from the shapes of the corresponding $\hat{\Delta S}$ filters by reversing the rescaling.

For large enough values of $\eta _{stp}$ (depression-dominated regime), the increase of $\hat{Z}$ with increasing values of $f_{spk}$ is much slower than the decrease of $\hat{X}$ (in the limiting case $\eta \rightarrow \infty$, $\hat{Z} \sim a_f$, a constant). Therefore, $\hat{\Delta S}$ is a LPF (e.g., Fig. 4-A and -B, blue). For small enough values of $\eta _{stp}$ (facilitation dominated regime), $\hat{Z}$ increases very fast with increasing values of $f_{spk}$ as compared to $\hat{X}$ (in the limiting case $\eta _{spk} \rightarrow 0$, $\hat{Z}$ increases instantaneously and is approximately a constant, $\hat{Z} \sim 1$). Therefore, $\hat{\Delta S}$ is also a LPF.

The transition between these two LPFs as $\eta _{stp}$ changes occurs via the development BPFs (e.g., Fig. 4-D to -F, blue). Within some range of values of $\eta _{stp}$ in between the LPFs and BPFs, the $\bar{\Delta S}$ patterns develop a local minimum preceding the local maximum (e.g., Fig. 4-C, blue).

#### From single-event time constants to frequency filters: Control of the filters’ shape by ${\tau _{dep}}$ and ${\tau _{fac}}$

The shapes of the $\bar{X}$ LPFs and $\bar{Z}$ HPFs, and therefore the $\bar{\Delta S}$ BPFs, are controlled by the time constants $\tau _{dep}$ and $\tau _{fac}$ operating at the “single-event” level, which govern the depression and facilitation dynamics, respectively, in response to each presynaptic spike. This is done by the communication of the single event time constants to the global time constants characterizing the temporal filters in response to repeated presynaptic inputs (Mondal et al., 2022) in a presynaptic frequency-dependent manner. The $\bar{X}$, $\bar{Z}$ and $\bar{\Delta S}$ frequency filters are the steady-states of said temporal filters across presynaptic input frequencies.

**Fig. 5:**
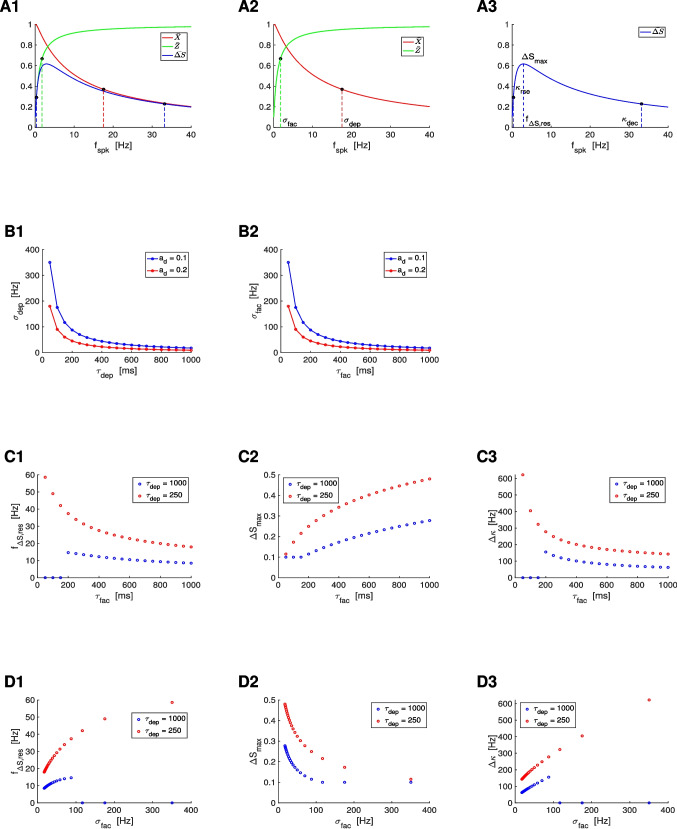
$\bar{\Delta S}$ **filters in response to periodic presynaptic spike inputs (frequency **$f_{spk}$**) for the DA model: frequency attributes.** **A.** The black dots on the $\bar{X}$, $\bar{Z}$ and $\bar{\Delta S}$ filters indicate the characteristic frequencies (projections on the $f_{spk}$ axis) defined as the change in the corresponding quantities by 63 % of the gap between their final and initial values ($\sigma _{dep}$ and $\sigma _{fac}$) and between their maximum and minimum values ($\kappa _{rse}$ and $\kappa _{dec}$). The $\bar{\Delta S}$ resonant frequency $f_{\Delta S,res}$ is the peak frequency and $\Delta S_{max}$ is the peak value. **B.** Dependence of the $\bar{X}$ and $\bar{Z}$ attributes (characteristic frequencies $\sigma _{dep}$ and $\sigma _{fac}$) with the depression and facilitation time constants $\tau _{dep}$ and $\tau _{fac}$, respectively. **C.** Dependence of the $\bar{\Delta S}$ attributes with $\tau _{fac}$ for representative values of $\tau _{dep}$. **D.** Dependence of the $\bar{\Delta S}$ attributes with $\sigma _{fac}$ for representative values of $\tau _{dep}$. For $\tau _{dep} = 1000$, $\sigma _{dep} \sim 17.6$, and for $\tau _{dep} = 250$, $\sigma _{dep} \sim 70.1$

We characterize the properties of the $\bar{X}$ and $\bar{Z}$ filters in terms of the characteristic frequencies $\sigma _{dep}$ and $\sigma _{fac}$, respectively (Fig. 5-A2). These attributes are defined as the frequencies for which the filters reached 63% of the gap between their values at $f_{spk} = 0$ and $f_{spk} \rightarrow \infty$ (black dots in Figs. 5-A1 and -A2). For the characterization of the $\bar{\Delta S}$ BPFs we use four attributes (Fig. 5-A3): the characteristic frequencies $\kappa _{rse}$ and $\kappa _{dec}$, the $\bar{\Delta S}$ resonant frequency $f_{\bar{\Delta S},res}$ and the peak frequency $\bar{\Delta S}_{max}$. The characteristic frequencies were computed as the frequency difference between the peak and the frequency value at which $\bar{\Delta S}$ reached 63% of the gap between the peak and the value at $f_{spk} = 0$ ($\kappa _{rse}$) and $f_{spk} \rightarrow \infty$ ($\kappa _{dec}$). The difference $\Delta \kappa = \kappa _{dec} - \kappa _{rse}$ is a measure of the spread of the BPFs.

Figure 5-B shows the dependence of the characteristic frequencies for the $\bar{X}$ and $\bar{Z}$ filters $\tau _{dep}$ and $\tau _{fac}$ (for representative values of $a_d$ and $a_f$). Specifically, $\sigma _{dep}$ and $\sigma _{fac}$ are decreasing functions of $\tau _{dep}$ and $\tau _{fac}$, respectively, and decreasing functions of $a_d$ and $a_f$, respectively. In other words, the larger the time constants, the more pronounced the decrease and increase of the corresponding filters with $f_{spk}$.

Figure 5-C shows the dependence of the attributes for the $\bar{\Delta S}$ BPFs with $\tau _{dep}$ and $\tau _{fac}$. We fixed the value of $\tau _{dep} = 1000$ (Fig. 5-C, blue) so the range of resonant frequencies $f_{\bar{\Delta S},res}$ is relatively low. Using this information one can obtain the dependences for other values of $\tau _{dep}$ by reversing the rescaling (43). For comparison, we also present the results for $\tau _{dep} = 250$ (Fig. 5-C, red). Specifically, the resonant frequency ($f_{\Delta S,res}$) decreases with increasing values of $\tau _{fac}$ and $\tau _{dep}$, the $\bar{\Delta S}$ peak increases with increasing values of $\tau _{fac}$ and decreases with increasing values of $\tau _{dep}$ and the peak becomes sharper ($\Delta \kappa$ decreases) as $\tau _{fac}$ or $\tau _{dep}$ increase. Figure 5-D shows the same results as a function of the characteristic frequency $\sigma _{fac}$.

### Interplay of ${\bar{\Delta S}}$ and ${\bar{S}}$ filters: inherited and cross-level mechanisms of generation of ${\bar{S}}$ BPFs

We characterize the steady state response profiles of $S$ to periodic presynaptic inputs by considering two attributes: the steady-state value $\bar{S}$ ($=\bar{S}_{peak}$) of the peak sequence $S_n$ ($= S_{peak,n}$) (Fig. 3, middle column, coral dots) and the peak-to-trough steady-state amplitude

45 $$\begin{aligned} \Gamma _S =\bar{S}_{peak}-\bar{S}_{trough} \end{aligned}$$

where $\bar{S}_{trough}$ is the steady-state value of the trough sequence $S_{trough,n}$ (Fig. 3, middle column, acquamarine dots). Figure 3 (middle column) illustrates that both $\bar{S}$ and $\Gamma _S$ vary with the input frequency $f_{spk}$. The temporal filtering properties of $S$ using these two attributes were investigated in Mondal et al. (2022).

#### The to-${\bar{\Delta S}}$ synaptic update model with instantaneous $S$ rise

Here we use the approximate to-$\bar{\Delta S}$ model (21) for the dynamics of the synaptic variable $S$ under the assumption of instantaneous rise time. By construction, the steady state value $\bar{S}$ is given by

46 $$\begin{aligned} \bar{S} = \bar{\Delta S} \end{aligned}$$

for all input frequencies $f_{spk}$ ($\bar{\Delta S}$ is the steady-state profile of the sequence $\Delta S_n$).

##### The ${\bar{S}}$ filtering properties are inherited from the synaptic update level

The peak envelope profiles $\bar{S}$ ($\bar{S}_{peak}$) are identical to the $\bar{\Delta S}$ profiles for all input frequencies $f_{spk}$. In the absence of STP, $\bar{S}$ is a constant ($=1$), while in the presence of STP, $\bar{S}$ inherits the filtering properties from the synaptic update level discussed above.

In order to calculate $\Gamma _S$, one needs to solve the differential equation (21) during the presynaptic ISIs ($\Delta S_{spk,n} = \Delta S_{spk}$) and update the solution at the occurrence of each presynaptic spike at $t = t_{spk,n}$ ($n = 1, \ldots , N_{spk}$), thus arriving to the following discrete linear difference equation for the trough sequences

47 $$\begin{aligned} S_{n+1} = e^{-\Delta _{spk}/\tau _{dec}}\, \Delta S_n. \end{aligned}$$

Therefore, the peak-to-trough envelope amplitude profile $\Gamma _S$ is given by

48 $$\begin{aligned} \Gamma _S = \bar{\Delta S}\, (\, 1 - e^{-\Delta _{spk}/\tau _{dec}}\, ). \end{aligned}$$

This expression is the product of two frequency-dependent processes: the $\bar{\Delta S}$ profile and

49 $$\begin{aligned} Q_A = 1 - e^{-\Delta _{spk}/\tau _{dec}}, \end{aligned}$$

which is a LPF. The shape of the $\bar{\Delta S}$ profile depends on the presence and properties of STP. In the absence of STP, the $\bar{\Delta S}$ profile is independent of $f_{spk}$ and $\Gamma _S$ is a LPF.

**Fig. 6:**
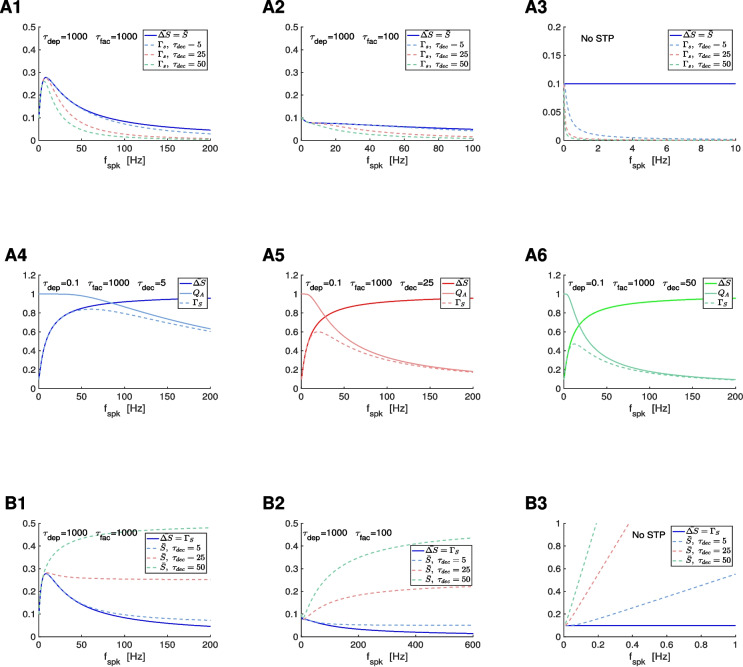
$S \;$ **and **$\Gamma _S$ **filters in response to periodic presynaptic spike inputs (frequency **$f_{spk}$**) for the to- and by-**$\Delta S\;$ **update models with instantaneous rise: representative examples.** We used Eqs. (12) and (13) (DA model) for $\bar{\Delta S}$. **A.** To-$\Delta S$ model (synaptic update to $\Delta S$). We used Eq. (48) for $\Gamma _S$ and Eq. (49) or $Q_A$. **A1.** $\bar{\Delta S}$ and $\Gamma _S$ are band-pass filters. **A2.** $\bar{\Delta S}$ and $\Gamma _S$ are low-pass filters. **A3.** No STP. $\bar{\Delta S}$ is constant and $\Gamma _S$ are low-pass filters. **A4 to A6.** $\Gamma _S$ band-pass filters generated from $\bar{\Delta S}$ high-pass filters and $Q_A$ low-pass filters. **B.** Synaptic update by $\Delta S$. We used Eq. (58) for $\bar{S}$. **B1.** $\bar{\Delta S}$ is a band-pass filter, while $\bar{S}$ transitions form band- to high-pass filters as $\tau _{dec}$ increases. **B2.** $\bar{\Delta S}$ is primarily a low-pass filter, while $\bar{S}$ transitions form low- to high-pass filters as $\tau _{dec}$ increases. **B3.** No STP. $\bar{\Delta S}$ is constant, while $\bar{S}$ are high-pass filters. We used the following additional parameter values: $a_d = 0.1$, $a_f = 0.1$, $x_{\infty } = 1$ and $z_{\infty } = 0$

As $f_{spk}$ increases, the $\Gamma _S$ profiles transition from $\Gamma _S = \bar{\Delta S}$ ($f_{spk} \rightarrow 0$) to $\Gamma _S = 0$ ($f_{spk} \rightarrow \infty$) (Fig. 6). For fixed values of $f_{spk}$, the $\Gamma _S$ profiles transition from $\Gamma _S = \bar{\Delta S}$ ($\tau _{dec} \rightarrow 0$) to $\Gamma _S = 0$ ($\tau _{dec} \rightarrow \infty$) as $\tau _{dec}$ increases. In other words, for small enough values of $\tau _{dec}$, the $\Gamma _S$ profiles reproduce the $\bar{\Delta S}$ profiles (Fig. 6-A1 to A3), but for larger values of $\tau _{dec}$, the $\Gamma _S$ and $\bar{\Delta S}$ profiles are different. These differences increase as $f_{spk}$ and $\tau _{dec}$ increase (Fig. 6). For generality, in Fig. 6 we included values of $\tau _{dec}$ beyond the biophysically plausible regime for AMPA excitation.

##### Inherited mechanisms of generation of $\varvec{\Gamma _S}$ BPFs

When $\bar{\Delta S}$ is constant (frequency-independent, no STP) or is an LPF, $\Gamma _S$ is a LPF (Fig. 6-A2 and A3). These LPFs become more pronounced as $\tau _{dec}$ increases. When $\bar{\Delta S}$ is a BPF, the $\Gamma _S$ BPFs evoked by $\bar{\Delta S}$ become sharper as $\tau _{dec}$ increases (Fig. 6-A1). These $\Gamma _S$ filters are inherited from the $\bar{\Delta S}$ ones and modulated by $\tau _{dec}$.

##### Cross-level mechanisms of generation of $\varvec{\Gamma _S}$ BPFs

In contrast, when $\bar{\Delta S}$ is a HPF, $\Gamma _S$ BPFs emerge as the product of a HPF and a LPF (Fig. 6-A4). As $\tau _{dec}$ increases, the $Q_A$ LPF is more pronounced as a function of $f_{spk}$ and therefore the $\Gamma _S$ BPF is sharper and peaks at a smaller value (compare Fig. 6-A4, -A5 and -A6).

#### The to-${\bar{\Delta S}}$ synaptic update model with non instantaneous $S$ rise

Here we focus on the effects of the synaptic rise time on the generation and modulation of synaptic filters, particularly synaptic BPFs. We use the approximate model (22) for the dynamics of the synaptic variable $S$ when the assumption of instantaneous rise is relaxed. The solution to the first and second terms in (22) are given by

50 $$\begin{aligned} S = \Delta S + [\, S(t_{spk,n}) - \Delta S \,]\, e^{-(t-t_{spk,n})/\tau _{rse}} \end{aligned}$$

and

51 $$\begin{aligned} S = [\, \Delta S + (\, S(t_{spk,n})-\Delta S\, )\, e^{-(T_{sw}/\tau _{rse}})\, ]\, e^{-(t-t_{spk,n}-T_{sw})/\tau _{dec}}, \end{aligned}$$

respectively. Using this, one can compute the difference equation governing the evolution of the sequence of peaks

52 $$\begin{aligned} S_{n+1} = \Delta S\, \left( \, 1 - e^{-T_{sw}/\tau _{rse}}\, \right) + S_n\, e^{-(\Delta _{spk,n}-T_{sw})/\tau _{dec}}\, e^{-T_{sw}/\tau _{rse}}. \end{aligned}$$

By assuming a constant $\Delta _{spk,n} = \Delta _{spk}$, one obtains

53 $$\begin{aligned} \bar{S} = \bar{\Delta S}\, \frac{1 - e^{-T_{sw}/\tau _{rse}}}{1-e^{-T_{sw}/\tau _{rse}}\, e^{-(\Delta _{spk}-T_{sw})/\tau _{dec}}\, } \end{aligned}$$

and

54 $$\begin{aligned} \Gamma _S = \bar{S}\, \left[ 1 -e^{-(\Delta _{spk}-T_{sw})/\tau _{dec}} \right] . \end{aligned}$$

Both expressions are the product of frequency-dependent filters and reduce to Eqs. (46) and (48) for $\tau _{rse} \rightarrow 0$ and $T_{sw} \rightarrow 0$ with $\tau _{rse} / T_{sw} \ll 1$.

We first focus on the $\bar{S}$ profiles. For $\tau _{rse} \rightarrow 0$, the second factor in Eq. (53)

55 $$\begin{aligned} Q_C = \frac{1 - e^{-T_{sw}/\tau _{rse}}}{1-e^{-T_{sw}/\tau _{rse}}\, e^{-(\Delta _{spk}-T_{sw})/\tau _{dec}}\, } \end{aligned}$$

approaches $Q_C = 1$ for all $f_{spk}$ and therefore the $\bar{S}$ profiles are approximately equal to the $\bar{\Delta S}$ profiles. For $\tau _{rse}> 0$, $Q_C$ is a HPF and therefore the $\bar{S}$ profiles depart from the $\bar{\Delta S}$ profiles

Specifically, for $\tau _{rse}> 0$, $Q_C$ changes from $Q_{C,0} = 1 - e^{-T_{sw}/\tau _{rse}} < 1$ (for $f_{spk} = 0$) to$$\begin{array}{c}Q_{C,\infty}=(1-e^{-T_{sw}/\tau_{rse}})/\end{array}$$ $(1-e^{-T_{sw}(1/\tau_{rse}-1/\tau_{dec})})>1$ (as $f_{spk} \rightarrow \infty$). $Q_C = 1$ for $f_{spk} = 1000/T_{sw}$, independently of $\tau _{dec}$ and $\tau _{rse}$. As $\tau _{rse}$ increases (all other parameters fixed), within some bounds, $Q_{C,0}$ decreases and $Q_{C,\infty }$ increases, causing an increase in the HPF amplitude of $Q_C$. As $\tau _{dec}$ increases (all other parameters fixed), also within some bounds, $Q_C$ increases for $0< f_{spk} < 1000/T_{sw}$ and decreases for $f_{spk}> 1000/T_{sw}$.

##### Attenuation of the $\varvec{\bar{S}}$ filters (BPFs, LPFs and HPFs) inherited from the synaptic update level

**Fig. 7:**
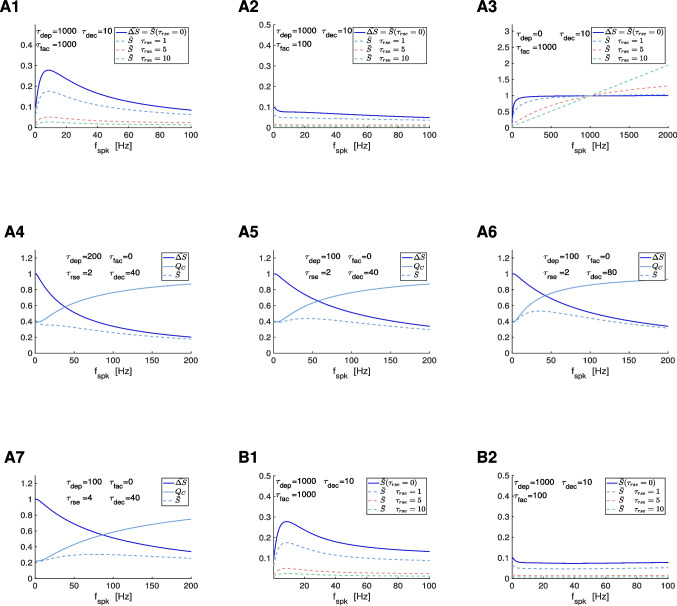
$\bar{S}$ **and **$\Gamma _S$ **filters in response to periodic presynaptic spike inputs (frequency **$f_{spk}$**) for the to- and by-**$\Delta S$ **update models with non-instantaneous rise: representative examples.** We used Eqs. (12) and (13) (DA model) for $\bar{\Delta S}$ **A.** To-$\Delta S$ model (synaptic update to $\Delta S$). The $S$ and $\Gamma _S$ filters were computed using Eqs. (53) and (54), respectively, with $Q_C$ and $Q_D$ given by Eqs. (55) and (56), respectively. **A1 to A3.** Effects of $\tau _{rse}$. **A1.** $\bar{S}$ BPFs attenuated by increasing values of $\tau _{rse}$. **A2.** $\bar{S}$ LPFs attenuated by increasing values of $\tau _{rse}$. **A3.** $\bar{S}$ HPFs filters attenuated (amplified) by increasing values of $\tau _{rse}$ for lower (higher) values of $f_{spk}$. **A4.** $\bar{S}$ low-pass filters created by the interplay of a $\bar{\Delta S}$ low-pass filter and a $Q_C$ high-pass filter. **A5, A6, A7.** $\bar{S}$ band-pass filters created by the interplay of $\bar{\Delta S}$ low-pass filters and $Q_C$ high-pass filters. **B.** By-$\Delta S$ model (synaptic update by $\Delta S$). The $S$ and $\Gamma _S$ filters were computed using Eqs. (62) and (63), respectively, with $Q_E$ given by Eq. (64). We used the following additional parameter values: $a_d = 0.1$, $a_f = 0.1$, $x_{\infty } = 1$, $z_{\infty } = 0$ and $T_{sw} = 1$

Therefore, for $f_{spk} < 1000/T_{sw}$, increasing values of $\tau _{rse}$ cause an attenuation of the $\bar{S}$ profiles (Fig. 7-A1 to -A3), and this attenuation is less pronounced the larger $\tau _{dec}$ (not shown). For $f_{spk}> 1000/T_{sw}$, increasing values of $\tau _{rse}$ cause an amplification of the $\bar{S}$ profiles (Fig. 7-A3), which is less pronounced the larger $\tau _{dec}$ (not shown). However, for $T_{sw} = 1$, the latter range is well beyond the frequencies we are interested in this paper.

The bounds mentioned above are set by the requirement that the denominator of $Q_C$ is positive, which in turn requires that $\Delta _{spk}> T_{sw} (\tau _{rse}-\tau _{dec})/\tau _{rse}$. This is satisfied for all values of $\Delta _{spk}$ if $\tau _{rse} < \tau _{dec}$. (For larger values of $\tau _{rse}$, this imposes a bound on $\Delta _{spk}$ for which $Q_C> 0$.) The realistic values of $\tau _{rse}$ and $\tau _{dec}$ we use here satisfy this condition. Moreover, for these values of $\tau _{rse}$ and $\tau _{dec}$, $Q_C$ is a HPF, converging asymptotically to $Q_{C,\infty }$.

##### Cross-level mechanisms of generation of $\varvec{\bar{S}}$ BPFs and attenuation of $\varvec{\bar{S}}$ filters

Because $Q_C$ has HPF properties, the question arises whether a $\bar{S}$ BPF can be created by the interplay of a $\bar{\Delta S}$ LPF and $Q_C$ for nonzero values of $\tau _{rse}$. (For instantaneous rise times, $\bar{S}$ BPFs can only be inherited since $\bar{S} = \bar{\Delta S}$; see Section 3.2.1). Figures 7-A4 to -A7 illustrates that this is indeed possible $\tau _{rse}> 0$. The generation of a $\bar{S}$ band-pass filter requires that the $Q_C(0)$ is low enough, which is achieved by increasing $\tau _{rse}$ above some threshold value (Fig. 7-A5). This BPF can be amplified by making the increase of $Q_C$ sharper for low values of $f_{spk}$, which can be achieved by increasing $\tau _{dec}$ (Fig. 7-A6). The BPF in Fig. 7-A5 is attenuated by further increasing $\tau _{rse}$ since this causes the intersection between the constituents low- and high-pass filters to move down (Fig. 7-A7). An increase in the values of $\tau _{dep}$ (Fig. 7-A7) causes the $\bar{\Delta S}$ LPF to decrease sharper as compared to Fig. 7-A5, decreasing the intersection between the $\bar{\Delta S}$ LPF and the $Q_C$ HPF. The resulting attenuation produces a $\bar{S}$ LPF (Fig. 7-A7).

##### Cross-level mechanisms of generation of $\varvec{\Gamma _S}$ BFPs and attenuation of $\varvec{\Gamma _S}$ filters

The second factor in Eq. (54),

56 $$\begin{aligned} Q_D = 1 -e^{-(\Delta _{spk}-T_{sw})/\tau _{dec}}, \end{aligned}$$

is a LPF, provided $\Delta _{spk}$ is large enough as compared to $T_{sw}$, and is independent of $\tau _{rse}$. Therefore, the effect of $\tau _{rse}$ on the $\Gamma _S$ filters is inherited from the effect of $\tau _{rse}$ on $\bar{S}$ filters. The $\Gamma _S$ filters are further attenuated by the $Q_D$ filter. The $\Gamma _S$ BPFs, in addition, become wider and the $\Gamma _S$ resonant frequency is displaced (Fig. S14-A1, -A3). The attenuation is more pronounced for the larger frequencies as $\tau _{dec}$ increases, therefore the BPFs become sharper and the $\Gamma _S$ resonant frequency is displaced as $\tau _{dec}$ increases (not shown).

### Interplay of ${\bar{\Delta S}}$ and summation PSP filters

The synaptic variable $S$ determined by Eq. (3) is the input to the current balance equation (1) for the postsynaptic voltage response $V$. In the absence of STP ($\Delta S = 1$), summation effects give rise to postsynaptic (PSP) HPFs whose properties depend on the membrane potential properties, particularly the membrane time constant ($\tau$). In the presence of STP, the PSP filters reflect the interaction of the $\bar{\Delta S}$ filters and the summation filters. For small enough values of $\tau$, the PSP filters are well approximated by the $\bar{\Delta S}$ filters. Previous work by other authors has considered PSP and $\bar{\Delta S}$ filters to be proportional (e.g., Markram et al., 1998; Markram et al., 1998b; Markram et al., 1998a, but see Drover et al., 2007). However, for larger values of $\tau$, the PSP filters are expected to depart from the weak modulation of the $\bar{\Delta S}$ filters.

Here we us the “by-$\Delta S$” model (23) described in Section 2.2.2 as an intermediate step for the investigation of the mechanisms that govern the interaction between the $\bar{\Delta S}$ and PSP summation filters. We investigate the response of conductance-based models to periodic presynaptic inputs in the presence of STP in the next Section. The simplified model we study here has the advantage of being amenable to analytical calculations that provide a better insight into the mechanistic aspects of the interaction between the $\bar{\Delta S}$ and PSP summation filters as compared to conductance-based models, and therefore pave the way for the investigation of said models.

#### The by-${\bar{\Delta S}}$ update model with instantaneous $S$ rise

As a first step, we focus on instantaneous rise for $S$. In contrast to the to-$\bar{\Delta S}$ model, instantaneous rise is not a natural assumption when $S$ is interpreted as the PSP because of the effects of the membrane time constant.

By solving the differential equation (23) for a constant value of $\Delta S_{spk,n} = \Delta S_{spk}$ during the presynaptic ISIs and updating the solution at each occurrence of the presynaptic spikes at $t = t_n$, $n = 1, \ldots$, one arrives to the following discrete linear differential equation for the peak sequences in terms of the model parameters

57 $$\begin{aligned} S_{n+1} = e^{-\Delta _{spk}/\tau _{dec}}\, S_n+ \Delta S_n. \end{aligned}$$

The $\bar{S}$ profile is given by the steady state values of (57)

58 $$\begin{aligned} \bar{S} = \frac{\bar{\Delta } S}{1 - e^{-\Delta _{spk}/\tau _{dec}}}. \end{aligned}$$

By construction, the $\Gamma _S$ profiles are

59 $$\begin{aligned} \Gamma _S = \bar{\Delta S}. \end{aligned}$$

In other words, the $\Gamma _S$ filtering properties are inherited from the $\bar{\Delta S}$ profiles.

##### The $\varvec{\bar{S}}$ and $\varvec{\bar{\Delta S}}$ profiles depart from each other for relatively large values of $\varvec{f_{spk}}$

Equation (58) is the product of two frequency-dependent processes. The factor

60 $$\begin{aligned} Q_B = \frac{1}{1 - e^{-\Delta _{spk}/\tau _{dec}}} \end{aligned}$$

is a HPF. It transitions from $Q_B = 1$ (for $f_{spk}=0$) to $Q_B \rightarrow \infty$ (for $f_{spk} \rightarrow \infty$), and it increases faster the larger $\tau _{dec}$. As already discussed, the shape of the $\bar{\Delta S}$ profile depends on the presence and properties of STP.

From Eq. (58), for small enough values of $f_{spk}$, $\bar{S} \sim \bar{\Delta S}$. In the limit $f_{spk} \rightarrow 0$, $\bar{S} = \bar{\Delta S}$. As $f_{spk}$ increases, $Q_B$ increases and therefore the difference between $\bar{S}$ and $\bar{\Delta S}$ also increases.

##### $\varvec{\bar{S}}$ BPFs and LPFs transition to $\varvec{\bar{S}}$ HPFs as $\varvec{\tau _{dec}}$ increases in the presence of STP

In the absence of STP, $\bar{\Delta S}$ is constant and therefore $\bar{S}$ increases unboundedly as $f_{spk} \rightarrow \infty$ (Fig. 6-B3). Similarly unbounded $\bar{S}$ profiles are also obtained for $\bar{\Delta S}$ HPFs. Under certain circumstances, the presence of STP puts a bound on the increase of $\bar{S}$, particularly for large values of $\tau _{dec}$ and the resulting filters remain bounded. Specifically, $\bar{S}$ BPFs (Fig. 6-B1) and LPFs (Fig. 6-B2) may remain so for low enough values of $\tau _{dec}$ and transition to (bounded) HPFs for larger values of $\tau _{dec}$. However, these HPFs may reach saturation values that are too high to be realistic. This together with the presence of unbounded $\bar{S}$ profiles (e.g., Fig. 6-B3) suggests that more complex biophysical models we investigate below include mechanism that cause the summation effects to be realistically saturated. Note that in some cases (e.g., Fig. 6-B2), the transition to a HPF involves the generation of a trough in the $\bar{S}$ profiles for large enough values of $\tau _{dec}$.

#### The by-${\bar{\Delta S}}$ update model with non-instantaneous ${S}$ rise

Here we extend our results to include the effects of noninstantaneous rise time. We use the approximate model (24) for the dynamics of the variable S when the assumption of instantaneous rise is relaxed.

The solution to the first and second terms in Eq. (24) are given by Eqs. (50) and (51), respectively, with $\Delta S$ substituted by $\hat{\Delta S}$. Using this, one can compute the difference equation governing the evolution of the sequence of peaks

61 $$\begin{aligned} S_{n+1} = \Delta S\, \left( \, 1 - e^{-T_{sw}/\tau _{rse}}\, \right) + S_n\, e^{-(\Delta _{spk,n}-T_{sw})/\tau _{dec}}. \end{aligned}$$

By assuming a constant $\Delta _{spk,n} = \Delta _{spk}$, one obtains the $\bar{S}$ and $\Gamma _S$ profiles

62 $$\begin{aligned} \bar{S} = \bar{\Delta S}\, \frac{1 - e^{-T_{sw}/\tau _{rse}}}{1-e^{-(\Delta _{spk}-T_{sw})/\tau _{dec}}} \end{aligned}$$

and

63 $$\begin{aligned} \Gamma _S = \bar{S}\, \left( 1 -e^{-(\Delta _{spk,n}-T_{sw})/\tau _{dec}} \right) = \bar{\Delta S}\, \left( \, 1 - e^{-T_{sw}/\tau _{rse}}\, \right) . \end{aligned}$$

The $\bar{S}$ profile is the product of frequency-dependent processes: $\bar{\Delta S}$ and

64 $$\begin{aligned} Q_E = \frac{1 - e^{-T_{sw}/\tau _{rse}}}{1-e^{-(\Delta _{spk}-T_{sw})/\tau _{dec}}}. \end{aligned}$$

$Q_E$ reduces to $Q_B$ in Eq. (49) for $\tau _{rse} \rightarrow 0$ and $T_{sw} \rightarrow 0$ with $\tau _{rse} / T_{sw} \ll 1$. This case was discussed in Section 3.3.1 and serves as a reference here.

##### $\varvec{\bar{S}}$ BFPs and LPFs are attenuated by increasing values of $\varvec{\tau _{rse}}$

The presence of $T_{sw}$ in $Q_E$ (for $\tau _{rse}> 0$) causes a decrease in the initial values of $Q_E(f_{spk}=0)$ and shrinks the range of values of $f_{spk}$ for which the denominator of $Q_E$ is positive to finite values: $f_{spk} < 1000/T_{sw}$. As $f_{spk} \rightarrow 1000/T_{sw}$, $Q_E$ increases unboundedly. This in turn causes $\bar{S}$ to increase unboundedly. However, this behavior is not alway monotonic. For low enough frequencies, but large enough to be within the range of realistic values we consider in this paper, the LPFs and BPFs (Fig. 7-B1 and -B2) inherited from the $\bar{\Delta S}$ profiles (at the synaptic update level) and modulated by $\tau _{dec}$ are attenuated by increasing values of $\tau _{rse}$ (as discussed above). Away from this range of frequencies, the $\bar{S}$ profiles for $\tau _{rse}> 0$ increase above the $\bar{S}$ profile of $\tau _{rse} \rightarrow 0$ as they grow unboundedly. Increasing values of $\tau _{dec}$ amplify the grow of the $\bar{S}$ profiles consistent with the results of $\tau _{rse} \rightarrow 0$.

##### $\varvec{\Gamma _S}$ profiles are uniformly attenuated by increasing values of $\varvec{\tau _{rse}}$

The second factor in Eq. (63) is independent of $f_{spk}$ and therefore increasing values of $\tau _{rse}$ attenuate the $\Gamma _S$ filters without affecting their types (Fig. S15).

### Digression: A heuristic explanation of summation and summation-mediated filters

Here and in the next sections we focus on the stationary membrane potential fluctuations of passive postsynaptic cells in response to periodic presynaptic inputs in the presence of STP (Fig. 1-A) for relatively fast synaptic rise and decay times (see Methods), consistent with AMPA excitation.

The passive postsynaptic cell is described by

65 $$\begin{aligned} C\, \frac{dV}{dt} = -G_L\, V + I_{in}(t)-I_{syn}(t), \end{aligned}$$

where the variable $V$ in Eq. (65) represents $V - V_{eq}$ in Eq. (1) with $V_{eq} = E_L + I_{app}/G_L$ and $I_{syn}$ is described by Eqs. (2)-(4) appropriately adapted (to account for the interpretation of $V$ as membrane potential fluctuations around the equilibrium). In this section, we use the analytical approximations described in Section 2.2.4 (see also Appendix A).

#### PSP response of passive cells to presynaptic spikes for individual input frequencies

**Fig. 8:**
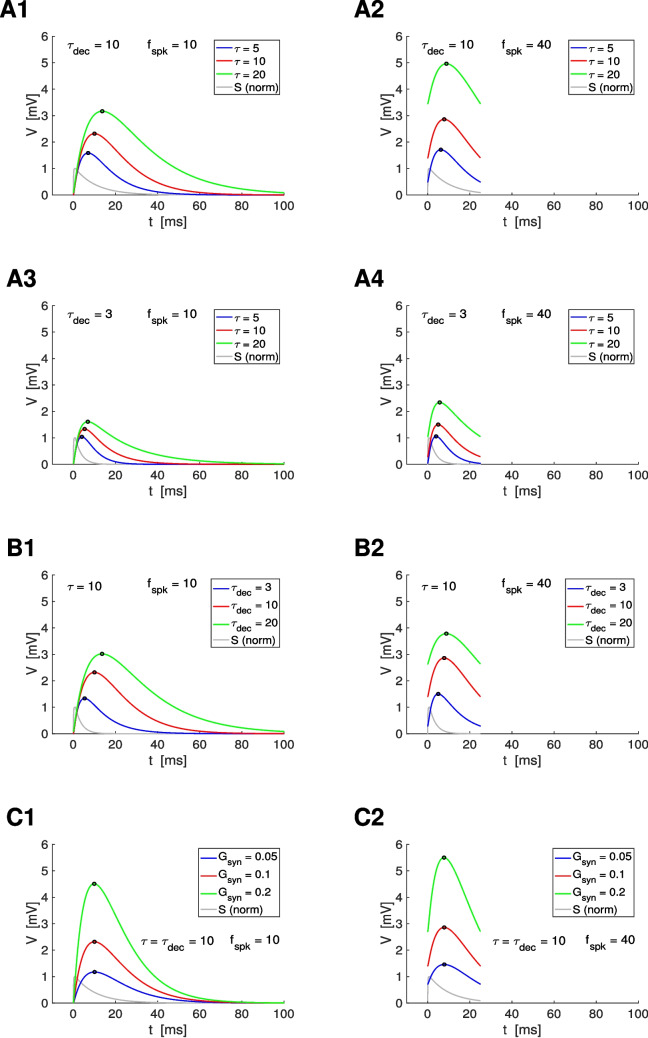
**Properties of the membrane potential response of passive cells to periodic presynaptic spikes for representative parameter values.** For the numerical approximations we used the model for a passive cell receiving presynaptic spike-train input (1)-(4). For STP we used the DA model (7)-(9). The graphs correspond to the steady-state solutions (translated to $t = 0$). The summation effect is observed in panels B. The synaptic function $S$ was normalized by its maximum in the presynaptic interspike interval. **A.** $G_{syn} = 0.1$ **Top row:** $\tau _{dec} = 10$. **Bottom row:** $\tau _{dec} = 3$. **Left column.** $f_{spk} = 10$. **Right column.** $f_{spk} = 40$. $G_{syn} = 0.1$. **B.** $G_{syn} = 0.1$ and $\tau = 10$ **B1.** $f_{spk} = 10$. **B2.** $f_{spk} = 40$. **C.** $\tau = 10$, $\tau _{dec} = 10$ **C1.** $f_{spk} = 10$. **C2.** $f_{spk} = 40$. We used the following additional parameter values: $a_d = 0.1$, $a_f = 0.1$, $x_{\infty } = 1$, $z_{\infty } = 0$, $\tau _{rse} = 0.1$, $C = 1$, $E_L = -60$, $I_{app} = 0$, $E_{syn} = -60$, $\tau _{dep} = \tau _{fac} = 0.1$

Here we analyze the properties of the steady-state PSP response of passive cells to presynaptic spikes within one ISI, from the arrival of a presynaptic spike ($t = t_n$) to the arrival of the next presynaptic spike ($t = t_{n+1} = \Delta _{spk,n}$). We then compare among the results for representative presynaptic ISIs (spiking input frequencies) to understand how the complex interaction among the participating time scales ($\tau _{dep}$, $\tau _{fac}$, $\tau _{dec}$, $\tau$, $\Delta _{spk}$) and maximal synaptic conductance $G_{syn}$ shape the PSP response profiles. Our results are presented in Fig. 8 for $f_{spk} = 10$ (left panels) and $f_{spk} = 40$ (right panels). We use the knowledge we gain from this analysis in the following sections.

By construction (see Section 2.2.4 and Appendix A.2), the analytical approximation to $V(t)$ consisting of $V_{I}(t)$ (78) for the duration of the presynaptic spike ($t_n< t < t_n + T_{sw}$) followed by $V_{II}(t)$ (81 or 88) for the reminder of the presynaptic ISI ($t_n + T_{sw}< t < t_n+\Delta _{spk,n}$), depends on the model parameters both explicitly and implicitly through the initial condition $V_{0,n}$ for each presynaptic ISI and the update parameter $\alpha _n$. The implicit dependence is inherited from the previous presynaptic ISI. We assume here the PSP response is in the steady-state regime and therefore we focus our analysis on the explicit dependence of $V(t)$ on the model parameters.

From Eq. (78) for $V_I(t)$ ($t_n< t < t_n + T_{sw}$), the larger $\tau$, the larger $\beta _n = V_I(t_n+T_{sw}) = V_II(t_n+T_{sw})$ (79). This dependence is affected by the presynaptic ISI $\Delta _{spk,n}$ through $V_{0,n} = V_{II}(t_{n-1})$ (from the previous presynaptic ISI). For the remainder of the presynaptic ISI ($t_n + T_{sw}< t < t_{n+1}$), from Eqs. (81) and (88), respectively,

66 $$\begin{aligned} V_{II}(t)&= \frac{\alpha _n\, \, \Delta S_n}{1-\eta }\, e^{-(t-t_n-T_{sw})\, \eta /\tau } \nonumber \\&\quad + \left[ \, \beta _n - \frac{\alpha _n\, \, \Delta S_n}{1-\eta }\, \right] \, e^{-(t-t_n-T_{sw})/\tau } \hspace{.5cm} \text{ if } \hspace{0.2cm} \tau _{dec} \ne \tau \end{aligned}$$

with

67 $$\begin{aligned} \eta = \frac{\tau }{\tau _{dec}}, \end{aligned}$$

and

68 $$\begin{aligned} V_{II}(t)&= \left[ \, \frac{\alpha _n\, \Delta S_n}{\tau }\, (t - t_n -T_{sw})\,+ \beta _n \right] \, e^{-(t-t_n-T_{sw})/\tau }\nonumber \\&\hspace{1cm} \text{ if } \hspace{0.3cm} \tau _{dec} = \tau . \end{aligned}$$

The PSP response $V(t)$ to presynaptic inputs is shaped by a complex balance among $\tau _{dec}$, $\tau$ and $\Delta _{spk,n}$ (Fig. 8), and is modulated by $G_{syn}$ (constant) and the STP time constants ($\tau _{dep}$ and $\tau _{fac}$) through $\Delta S_n$ (in a frequency-dependent manner), which determines the target for the peak of the synaptic function $S$. In Fig. 8, $\Delta S_n$ is independent of $f_{spk}$. The properties of the PSP response for frequency-dependent $\Delta S_n$ profiles are investigated in the next Sections.

If $\tau _{dec} \ll \tau$, $\eta \gg 1$ in Eq. (66) and $V_{II}(t)$ is dominated by the second term. In the limiting cases $\eta \rightarrow \infty$ ($\tau _{dec} \rightarrow 0$ or $\tau \rightarrow \infty$), $V_{II}(t)$ begins to decrease at $t_n + T_{sw}$. As $\eta$ decreases, $V_{II}(t)$ continues to increase passed $t_n + T_{sw}$ in response to $S_a(t)> 0$. The larger $\tau _{dec}$ for fixed values of $\tau$ , the larger $V_{II}(t)$ over the presynaptic ISI (Fig. 8-B) and the larger the peak time $t_{peak,n}$. Similarly, the larger $\tau$ for fixed values of $\tau _{dec}$, the larger $V_{II}(t)$ over the presynaptic ISI (Fig. 8-A) and the larger the peak time $t_{peak,n}$. If $\tau _{dec} \gg \tau$, $\eta \ll 1$ in Eq. (66) and $V_{II}(t)$ is dominated by the first term. In the limit $\eta \rightarrow 0$ ($\tau _{dec} \rightarrow \infty$ or $\tau \rightarrow 0$), $V_{II}(t)$ only increases over the presynaptic ISI. If $\tau _{dec} = \tau _{fac}$, $V_{II}(t)$ has the form of an alpha function. The larger $\tau$, the larger $V_{II}(t)$ over the presynaptic ISI. Increasing values of $G_{syn}$ also cause an increase in $V_{II}(t)$ over the presynaptic ISI (Fig. 8-C) with at most a mild effect on the peak times. This suggests that while increasing values of $\tau _{dec}$ and $G_{syn}$ increase the total input current to the postsynaptic cell, their effect on the properties of the PSP filters may differ.

Comparison between the left ($f_{spk} = 10$) and right ($f_{spk} = 40$) columns in Fig. 8 illustrates the effects of summation. One of them is the increase in the PSP peak response as $f_{spk}$ increases and the other one is the decrease in the peak-to-trough amplitude as $f_{spk}$ increases.

#### PSP Summation-mediated filters

By approximating $S$ by $\Delta S_n$ for the duration of the presynaptic spike ($t_n \le t \le t_n+T_{sw}$), the passive membrane equation receiving presynaptic inputs equation is approximated by

69 $$\begin{aligned} \tau \, \frac{dV}{dt} = - V + \gamma _{syn}\, \Delta S_n\, \tau \, (V - E_{syn}) \end{aligned}$$

where the variable $V$ has the same interpretation as in Eq. (25), $V(0) = 0$ and

70 $$\begin{aligned} \gamma _{syn} = \frac{G_{syn}}{C}. \end{aligned}$$

The solution to Eq. (69) is given by

71 $$\begin{aligned} V(t) = V_{\infty } + [\, V(t_n) - V_{\infty }\, ]\, e^{-(1/\tau +\gamma _{syn}\, \Delta S_n)\, (t-t_n)} \end{aligned}$$

where

72 $$\begin{aligned} V_{\infty } = \frac{\gamma _{syn}\, \Delta S_n\, \tau }{1+ \gamma _{syn}\, \Delta S_n\, \tau }\, E_{syn}. \end{aligned}$$

$V(t)$ increases and approaches its steady-state value $V_{\infty }$, which increases from $V_{\infty } = 0$ ($\tau = 0$) to $V_{\infty } = E_{syn}$ ($\tau \rightarrow \infty$). $V(t)$ reaches its peak value

73 $$\begin{aligned} \tilde{V}_{peak,n}&= V (t_n+T_{sw}) = V_{\infty }\, [\, 1 - e^{-(1/\tau +\gamma _{syn}\, \Delta S_n)\, T_{sw}}\, ] \nonumber \\&\quad + V(t_n)\, e^{-(1/\tau +\gamma _{syn}\, \Delta S_n)\, T_{sw}} < V_{\infty } \end{aligned}$$

during the presynaptic ISI. For the remaining of the presynaptic ISI ($t_n + T_{sw}< t < t_{n+1}$), $V(t)$ decays exponentially to some value $\tilde{V}_{trough,n} = V(t_{n+1})> 0$, which depends on the presynaptic ISI $\Delta _{spk,n}$.

The peak value $\tilde{V}_{peak,n}$ increases from $\tilde{V}_{peak,n} = 0$ ($\tau = 0$) to $\tilde{V}_{peak,n} = E_{syn} + [\, V(t_n) - E_{syn}\, ]\ e^{-\gamma _{syn}\, \Delta S_n\, T_{sw}}$ ($\tau \rightarrow \infty$), and is an increasing function of $V(t_n)$ whose value is inherited from the previous presynaptic ISI.

For periodic presynaptic inputs and synaptic update values $\Delta S_n$ independent of $n$, the $V_{peak}$ and $\Gamma _V$ summation are originated in the temporal domain (as $n$ increases) and are frequency-dependent. For each value of $f_{spk}$, $V(t_2) = V_{trough,1}> V(t_1) = 0$ and therefore, $V_{peak,2}> V_{peak,1}$. This causes $V_{trough,2} = V_{trough,1}$. Following this process for increasing values of $n$, leads to two monotonically non-decreasing sequences converging to $V_{peak}$ and $V_{trough}$. As $f_{spk}$ increases, $V_{trough,n}$ also increase for fixed values of $n$, since there is less time for $V$ to decay, and therefore $V_{peak,n}$ also increase.

**Fig. 9:**
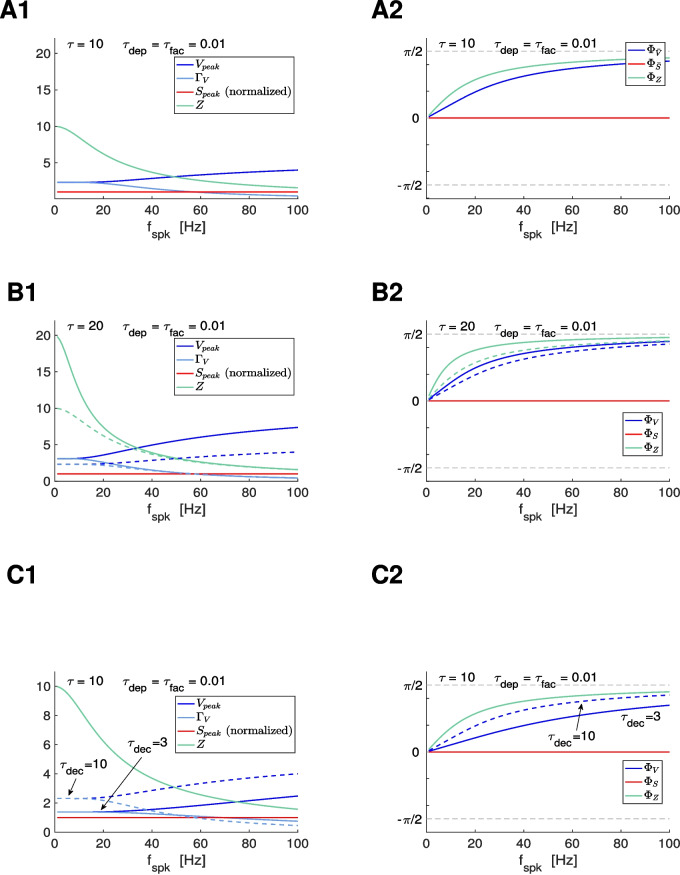
**Postsynaptic filters in response to periodic presynaptic spike inputs for the passive (postsynaptic) cell in the absence of STP.** We used Eq. (65) for the PSP $V$ with $I_{syn}$ described by Eqs. (2)-(4) appropriately adapted to account for the translation of $V$ to the equilibrium point. We used Eqs. (12) and (13) (DA model) with very values of $\tau _{dep}$ and $\tau _{fac}$ in the no STP regime. The impedance amplitude ($Z$) and phase ($\Phi _Z$) were computed using Eqs. (39) and (40). The analytical approximations for the PSP peak sequence response of passive cells to presynaptic inputs are described in Section 2.2.4 (see also Appendix A). The approximation of $V_{peak,n}$, $V_{trough,n}$ and $t_{V,peak}$ were computed as described in Section 2.2.4. The PSP amplitude $\Gamma _V$ was computed by using Eq. (41) and the PSP phase $\Phi _V$ was computed using Eq. (42). The synaptic ($S$) peak ($S_{peak}$) and phase ($\Phi _S$) profiles were computed similarly to these for $V$. **A.** $\tau = 10$ ($G_L = 0.1$), $\tau _{dec} = 10$. **B.** $\tau = 20$ ($G_L = 0.05$), $\tau _{dec} = 10$. The dashed curves correspond to panels B ($\tau =10$) and are presented for comparison purposes. **C.** $\tau = 10$ ($G_L = 0.1$), $\tau _{dec} = 3$. The dashed curves correspond to panels B ($\tau _{dec} =10$) and are presented for comparison purposes. We used the following additional parameter values: $C = 1$, $E_L = -60$, $I_{app} = 0$, $G_{syn} = 0.1$, $E_{syn} = 0$, $a_d = 0.1$, $a_f = 0.1$, $x_{\infty } = 1$, $z_{\infty } = 0$ and $T_{sw} = 1$

Therefore, $V_{peak}$ and $V_{trough}$ are increasing functions of $f_{spk}$. If the $V_{peak}$ profile increases slower than $V_{trough}$ profile, then the $\Gamma _V$ profile is a LPF (Fig. 9, column 1). For $\tau \rightarrow 0$, the $V_{peak}$ profile is proportional to $S_{peak}$ and $V_{peak} \rightarrow 0$. As $\tau$ increases, both the $V_{peak}$ and $\Gamma _V$ profiles are amplified.

### The response of passive cells to direct (sinusoidal) and indirect (periodic presynaptic) inputs shows different filtering properties

Here and in the next sections, using the insights and intuition gained in Sections 3.3 and 3.4, we first discuss the PSP summation HPFs in passive cells (controlled by $\tau$ and modulated by $\tau _{dec}$) in the absence of STP, and their link to the passive cells’ LPFs (captured by $Z$, also controlled by $\tau$). We then discuss the PSP filtering properties of passive cells in response to periodic presynaptic inputs in the presence of either depression (LPF) or facilitation (HPF).

The PSP filtering properties, captured by the $V_{peak}$, $\Gamma _V$ and $\Phi _V$ profiles, depend on the filtering properties of the participating building blocks and are controlled by the time constants operating at each level (Fig. 1): $\tau _{dep}$ and $\tau _{fac}$ (STP), $\tau _{dec}$ (synaptic) and $\tau$ (postsynaptic). From our results in Section 3.2 (see also Section 2.2.4), the steady-state $S$ peak profiles $S_{peak}$ (or $\bar{S}$) depend at most mildly on $\tau _{rse}$ for the type of fast synaptic rise times we consider here.

#### PSP ${V_{peak}}$ (LPFs; ${Z}$), ${\Gamma _V}$ (LPFs; ${Z}$) and ${\Phi _Z}$ (delay) profiles in response to direct activation of oscillatory inputs (revisited)

Passive cells are LPFs in response to direct activation of sinusoidal input currents and always exhibit a delayed response (see Section 2.3.2). The $Z$ (amplitude) profile (39) is a decreasing function of the input frequency $f$ (Fig. 9-A1, green) and the $\Phi _Z$ profile (40) is a positive increasing function of $f$, converging to $\pi / 2$ (Fig. 9-A2, green). These profiles are affected by the membrane time constant $\tau = C / G_L$. Increasing values of $\tau$ cause (i) an increase in $Z_{max} = Z(0)$ (compare Figs. 9-A1 and B1, green), (ii) a sharper decrease of the $Z$ profile (compare Figs. 9-A1 and B1, green), and (iii) a sharper increase of the $\Phi _Z$ profile (compare Figs. 9-A2 and B2, green).

#### PSP ${V_{peak}}$ (HPFs), ${\Gamma _V}$ (LPFs) and ${\Phi _V}$ (delay) profiles in response to periodic presynaptic inputs

In response to periodic presynaptic inputs (with no STP), passive cells are $\Gamma _V$ LPFs (Fig. 9, left column, light blue), but $V_{peak}$ HPFs (Fig. 9, left column, blue), while always exhibit a delayed response (Fig. 9, right column, blue) similarly to $\Phi _Z$. The $V_{peak}$ HPFs result from the summation phenomenon and are different in nature from the HPFs resulting from synaptic facilitation.

These profiles are modulated by both $\tau$ (membrane time constant) and $\tau _{dec}$ (synaptic decay time). Increasing values of $\tau$ cause (i) an amplification of the $V_{peak}$ profiles (compare Figs. 9-A1 and B1, blue), (ii) a sharper increase of the $V_{peak}$ profile with $f_{spk}$ (compare Figs. 9-A1 and B1, blue), (iii) an amplification of the $\Gamma _V$ profiles, which is more pronounced for the lower values of $f_{spk}$ (compare Figs. 9-A1 and B1, light blue), (iv) a sharper decrease in the $\Gamma _V$ profile with increasing values of $f_{spk}$ (compare Figs. 9-A1 and B1, green), and (v) a sharper increase in the $\Phi _V$ profile with increasing values of $f_{spk}$ (compare Figs. 9-A2 and B2, blue). Increasing values of $\tau _{dec}$ cause (i) an amplification of both the $V_{peak}$ and $\Gamma _V$ profiles (Fig. 9-C1, blue and light blue), (ii) a sharper decrease of the $\Gamma _V$ profiles with increasing values of $f_{spk}$ (Fig. 9-C1, light blue), and (iii) a sharper increase of the $\Phi _V$ profiles with increasing values of $f_{spk}$ (Fig. 9-C2, blue).

### PSP ${V_{peak}}$ and ${\Gamma _V}$ profiles: Inherited and cross-level mechanisms of generation of BPFs (PSP resonance)

The $V_{peak}$, $\Gamma _V$ and $\Phi _V$ profiles are shaped by the feedforward interaction of time scales and other constitutive properties across the participating building blocks (Fig. 1-B). Similarly to the BPFs generated by the simplified model used in Section 3.2, PSP BPFs can be inherited from one level (e.g, synaptic update) to another (PSP) or can be generated as the result of the interplay of LPFs and HPFs across levels.

For small enough values of $\tau _{dec}$ and $\tau$, the $V_{peak}$ profile is approximately proportional to the $S_{peak}$ profile, the $V_{trough}$ profile is almost zero, and therefore the $\Gamma _V$ profile is approximately equal to the $V_{peak}$ profile. In the limit of $\tau \rightarrow 0$ and $\tau _{dec} \rightarrow 0$ these relationships are strictly valid. In this sense, the PSP $V_{peak}$ and $\Gamma _V$ profiles (and the filtering properties) are inherited from the synaptic level $S_{peak}$ profile.

If $\tau _{dec}$ or $\tau$ increase slightly, the $V_{peak}$ and $\Gamma _V$ profiles are modulated versions of the $S_{peak}$ profiles. Specifically, if $\tau _{dec}$ increases ($\tau \ll 1$), then the $V_{peak}$ profile remains almost proportional to $S_{peak}$ profile, but the $V_{trough}$ profile increases with $f_{spk}$ and therefore the $\Gamma _V$ profile is lower than the $V_{peak}$ profile. If, on the other hand, $\tau$ increases ($\tau _{dec} \ll 1$), the $V_{peak}$ profile is no longer proportional to the $S_{peak}$ profile and the $V_{trough}$ profile is an increasing function of $f_{spk}$, and therefore the $\Gamma _V$ and $V_{peak}$ profiles are different.

If $\tau _{dec}$ or $\tau$ increase further, then the $V_{peak}$ and $\Gamma _V$ profiles main remain modulated versions of the $S_{peak}$ profiles or have a qualitatively different shape from the $S_{peak}$ profiles. Similarly to the generation of the simplified STP-mediated $\bar{S}$ (PSP) BPFs discussed in Section 3.2, under certain balance conditions, synaptic and postsynaptic filters with opposite monotonic dependencies (with $f_{spk}$) are expected to produce BPFs, while synaptic and postsynaptic filters with the same monotonic dependencies (with $f_{spk}$) are expected to reinforce each other.

**Fig. 10:**
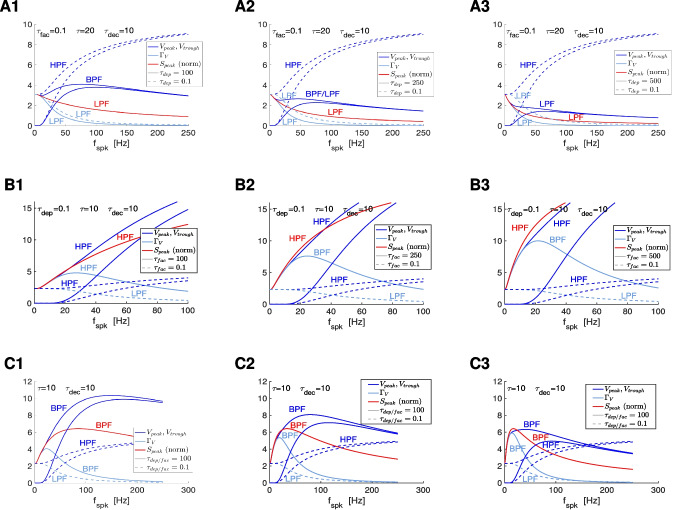
**Postsynaptic BPFs filters in response to periodic presynaptic spike inputs emerging from the interplay of short-term depression (LPF), short-term facilitation (HPF) and postsynaptic summation (HPF).** Each panel shows the superimposed $V_{peak}$, $V_{trough}$ and $\Gamma _V$ profiles in response to presynaptic inputs in the presence (solid) and absence (dashed) of STP. The $S_{peak}$ profiles are normalized to coincide with the $V_{peak}$ profiles for the lowest value of $f_{spk }$ ($f_{spk} = 0.1$ in the simulations). The summation HPFs are generated in response to presynaptic inputs in the absence of STP ($\tau _{dep} = 0.1$, $\tau _{fac} = 0.1$ or $\tau _{dep} = \tau _{fac} = 0.1$ in the simulations). The $S_{peak}$ profiles in the absence of STP are horizontal lines (not shown). **A.** $V_{peak}$ BFPs (solid blue) generated by the interplay of $S_{peak}$ LPFs (red) and $V_{peak}$ summation HPFs (dashed blue) in the presence of synaptic depression only. The interplay of the $\Gamma _V$ summation LPF (dashed light blue) and the $S_{peak}$ LPF (red) produces a $\Gamma _V$ LPF (solid light blue). **B.** $\Gamma _V$ BFPs (light blue) generated by the interplay of $S_{peak}$ HPFs (red) and $\Gamma _V$ summation LPFs (dashed blue) in the presence of synaptic facilitation only. The interplay of of the $V_{peak}$ summation HPF (dashed blue) and the $S_{peak}$ HPF (red) produces a $V_{peak}$ HPF (solid blue). **C.** $V_{peak}$ BPFs (solid blue) and $\Gamma _V$ BPFs (solid light blue) generated by the interplay of the inherited $S_{peak}$ BFPs (red) and modulated by the $V_{peak}$ summation HPFs (dashed blue) and the $\Gamma _V$ summation LPFs (dashed light blue). We used Eq. (65) for the PSP $V$ with $I_{syn}$ described by Eqs. (2)-(4) appropriately adapted to account for the translation of $V$ to the equilibrium point, and STP described by Eqs. (12) and (13) (DA model). The impedance amplitude ($Z$) and phase ($\Phi _Z$) were computed using Eqs. (39) and (40). The analytical approximations for the PSP peak sequence response of passive cells to presynaptic inputs are described in Section 2.2.4 (see also Appendix A). The approximation of $V_{peak,n}$ and $V_{trough,n}$ were computed as described in Section 2.2.4. The PSP amplitude $\Gamma _V$ was computed by using Eq. (41) and the PSP phase $\Phi _V$ was computed using Eq. (42). The synaptic ($S$) peak ($S_{peak}$) and phase ($\Phi _S$) profiles were computed similarly to these for $V$. We used the following additional parameter values: $C = 1$, $E_L = -60$, $I_{app} = 0$, $G_{syn} = 0.1$, $E_{syn} = 0$, $a_d = 0.1$, $a_f = 0.1$, $x_{\infty } = 1$, $z_{\infty } = 0$ and $T_{sw} = 1$

The interplay of synaptic depression (LPF) and $V_{peak}$ summation (HPF) is able to generate $V_{peak}$ BPFs (Fig. 10-A1, solid blue), but not $\Gamma _V$ BPFs (Fig. 10-A, solid light blue) since both the $S_{peak}$ and the $\Gamma _V$ profiles are LPFs. In contrast, the interplay of synaptic facilitation (HPF) and $\Gamma _V$ summation (LPF) is able to generate $\Gamma _V$ BPFs (Fig. 10-B, solid light blue), but not $V_{peak}$ BPFs since both the $S_{peak}$ and $V_{peak}$ summation profiles are HPFs (Fig. 10-B, solid blue). In the presence of both synaptic depression (LPF) and facilitation (HPF), the $S_{peak}$ profiles are BPFs (Fig. 10-C, solid red). These are communicated to the PSP level where they are modulated by the postsynaptic membrane potential properties. In certain parameter regimes, these modulations produce PSP $V_{peak}$ and $\Gamma _V$ BPFs (Fig. 10-C, solid blue).

We analyze the various possible scenarios and mechanisms in the next sections.

### Interplay of STD-mediated LPFs and PSP SUM-mediated HPFs: $V_{peak}$ BPFs and $\Gamma _V$ LPFs

**Fig. 11:**
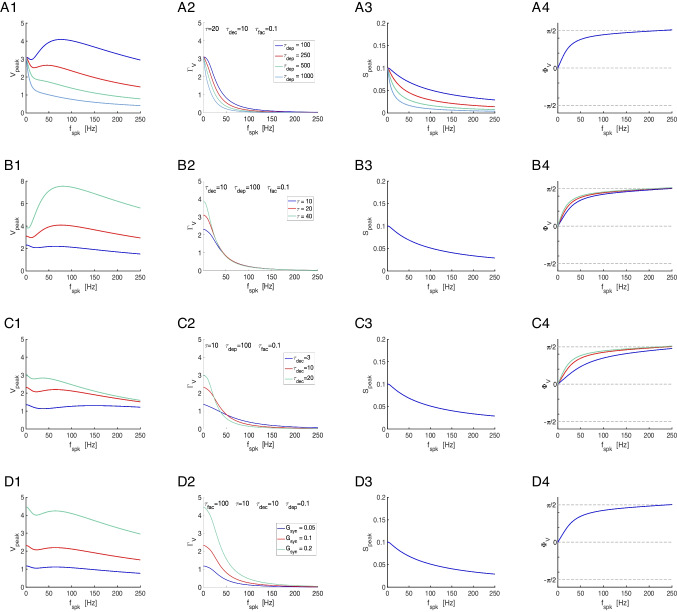
**Postsynaptic filters in response to periodic presynaptic spike inputs emerging from the interplay of short-term depression and postsynaptic summation. A.** Superimposed filters for various values of the short-term depression time constant $\tau _{dep}$ and representative parameter values: $\tau = 10$, $\tau _{dec} = 10$ and $\tau _{fac} = 0.1$. Figure S5 extends these results for additional values of $\tau$. The $\Phi _V$ profiles are independent of $\tau _{dep}$. **B.** Superimposed filters for various values of the postsynaptic membrane time constant $\tau$ and representative parameter values: $\tau _{dec} = 10$, $\tau _{dep} = 100$ and $\tau _{fac} = 0.1$. Figure S6 extends these results for additional values of $\tau _{dep}$. The $S_{peak}$ profiles are independent of $\tau _{dep}$. **C.** Superimposed filters for various values of the synaptic decay time $\tau _{dec}$ and representative parameter values: $\tau = 10$, $\tau _{dep} = 100$ and $\tau _{fac} = 0.1$. The $S_{peak}$ profiles are independent of $\tau _{dep}$. Figure S7 extends these results for additional values of $\tau _{dep}$. **D.** Superimposed filters for representative values of the synaptic decay time constant $G_{syn}$ and representative parameter values: $\tau _{fac} = 100$, $\tau = 10$, $\tau _{dec} = 10$ and $\tau _{dep} = 0.1$. Figure S10 extends these results for additional values of $\tau _{fac}$. The $S_{peak}$ and $\Phi _V$ profiles are independent of $G_{syn}$. **Left column.** $V$ peak profiles. **Middle-left column.** $V$ peak-to-trough amplitude profiles. **Middle-right column.** $S$ peak profiles. **Right column.** $V$ phase profiles. We used Eq. (65) for the PSP $V$ with $I_{syn}$ described by Eqs. (2)-(4) appropriately adapted to account for the translation of $V$ to the equilibrium point, and STP described by Eqs. (12) and (13) (DA model). The impedance amplitude ($Z$) and phase ($\Phi _Z$) were computed using Eqs. (39) and (40). The analytical approximations for the PSP peak sequence response of passive cells to presynaptic inputs are described in Section 2.2.4 (see also Appendix A). The approximation of $V_{peak,n}$, $V_{trough,n}$ and $t_{V,peak}$ were computed as described in Section 2.2.4. The PSP amplitude $\Gamma _V$ was computed by using Eq. (41) and the PSP phase $\Phi _V$ was computed using Eq. (42). The synaptic ($S$) peak ($S_{peak}$) and phase ($\Phi _S$) profiles were computed similarly to these for $V$. We used the following additional parameter values: $C = 1$, $E_L = -60$, $I_{app} = 0$, $G_{syn} = 0.1$, $E_{syn} = 0$, $a_d = 0.1$, $a_f = 0.1$, $x_{\infty } = 1$, $z_{\infty } = 0$ and $T_{sw} = 1$

The presence of synaptic STD generates $S_{peak}$ LPFs that become sharper and more attenuated as $\tau _{dep}$ increases (Fig. 11-A3) and are independent of $\tau$, $G_{syn}$ and $\tau _{dec}$ (Fig. 11-B3 to -D3)

#### Emergence of $V_{peak}$ resonance (BPFs): interplay of STD-mediated LPF and a PSP summation-mediated HPF

The interaction between these STD-mediated LPFs and the PSP summation-mediated HPF (Fig. 9-A1, blue) produces $V_{peak}$ BPFs for values of $\tau _{dep}$ within some range (Fig. 11-A1, blue and red). We refer to this preferred frequency PSP peak response to periodic presynaptic inputs as (PSP) $V_{peak}$ resonance. This maximal amplification of the $V_{peak}$ response is often preceded by a relatively small trough. $V_{peak}$ resonance reflects balances between the two participating processes. As $\tau _{dep}$ increases (sharper decrease of the $S_{peak}$ profile), the $V_{peak}$ profiles are dominated by depression and therefore they are attenuated as they transition to LPFs for larger values of $\tau _{dep}$ (Fig. 11-A1, blue to light blue). This is accompanied by a decrease in the $V_{peak}$ resonant frequency. As $\tau$ increases, the $V_{peak}$ profiles are dominated by summation and therefore they are amplified (Fig. 11-A2). This is accompanied by an increase in the $V_{peak}$ resonant frequency. Changes in $\tau$ and $G_{syn}$ do not affect the $S_{peak}$ profiles, but they affect the $V$ response to presynaptic spikes in a frequency-dependent manner (Fig. 8). Consistently with that, increasing values of $\tau$ and $G_{syn}$ amplify the $V_{peak}$ response with lesser effects on their shapes than changes in $\tau _{dep}$ and $\tau$ (Fig. 11-C1 and -D1). This is more prominent for $G_{syn}$, which has almost a multiplicative effect on the $V_{peak}$ profiles, than for $\tau$, consistently with the different ways in which they control the synaptic currents.

#### Modulation of $\Gamma _V$ LPFs: interplay of STD and PSP amplitude LPFs

In the absence of STP, the $\Gamma _V$ LPF is controlled by the membrane time constant $\tau$ (Fig. 9-B1) and the synaptic decay time constant $\tau _{dec}$ (Fig. 9-C1). Here we focus on the effects of synaptic depression ($\tau _{dep}$) and the interplay between the three time constants in the modulation of $\Gamma _V$. Increasing values of $\tau _{dep}$ sharpen $\Gamma _V$ without affecting $\Gamma _V(0)$ (Fig. 11-A2). The magnitude of the modulation depend on the other parameter values (Fig. S5, column 2). Increasing values of $\tau$ and $\tau _{dec}$ sharpen $\Gamma _V$ and increase $\Gamma _V(0)$ (Fig. 11-B2 and -C2). This is at most mildly affected by $\tau _{dep}$ (Fig. S7, column 2) and $\tau _{dec}$ (Fig. S8, column 2). Increasing values of $G_{syn}$ have a multiplicative effect on $\Gamma _V$ (Fig. 11-D2).

#### Modulation of the $\Phi _V$

In the absence of STP, the $\Phi _V$ profiles are controlled by $\tau$ and $\tau _{dec}$ ( Fig. 9). Changes in $\tau _{dep}$ do not affect $\Phi _V$ (Fig. 11-A4 and S5). Changes in $\tau$ and $\tau _{dec}$ affect $\Phi _V$ in the same direction as in the absence of STP (Figs. 11-B4, -A4, S6 and S7 ). Consistently with the previous findings, changes in $G_{syn}$ do not affect $\Phi _V$.

Figures S5-S7 extend our results for additional combinations of parameter values. Figures S5-S7 (left column), in particular, illustrate how the balances between the depression LPFs and peak summation HPFs shape the $V_{peak}$ profiles for additional parameter regimes.

### Interplay of STF-mediated HPFs and PSP SUM-mediated filters: $V_{peak}$ LPFs and $\Gamma _V$ BPFs

**Fig. 12:**
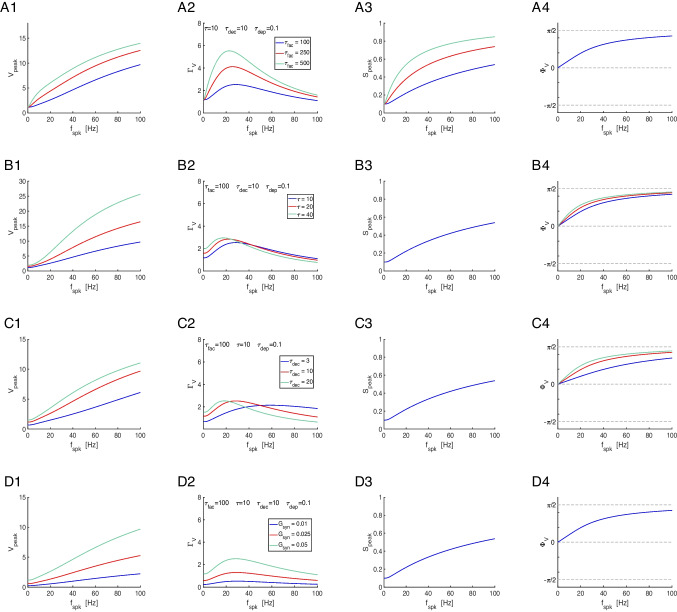
**Postsynaptic filters in response to periodic presynaptic spike inputs emerging from the interplay of short-term facilitation and postsynaptic summation.** **A.** Superimposed filters for various values of the short-term depression time constant $\tau _{fac}$ and representative parameter values: $\tau = 10$, $\tau _{dec} = 10$ and $\tau _{dep} = 0.1$. Figure S8 extends these results for additional values of $\tau$. The $\Phi _V$ profiles are independent of $\tau _{fac}$. **B.** Superimposed filters for various values of the membrane time constant $\tau$ and representative parameter values: $\tau _{fac} = 100$, $\tau _{dec} = 10$ and $\tau _{dep} = 0.1$. Figure S9 extends these results for additional values of $\tau _{fac}$. The $S_{peak}$ profiles are independent of $\tau$. **C.** Superimposed filters for representative values of the synaptic decay time constant $\tau _{dec}$ and representative parameter values: $\tau _{fac} = 100$, $\tau = 10$ and $\tau _{dep} = 0.1$. Figure S10 extends these results for additional values of $\tau _{fac}$. The $S_{peak}$ profiles are independent of $\tau _{dec}$. **D.** Superimposed filters for representative values of the synaptic decay time constant $G_{syn}$ and representative parameter values: $\tau _{fac} = 100$, $\tau = 10$, $\tau _{dec} = 10$ and $\tau _{dep} = 0.1$. Figure S10 extends these results for additional values of $\tau _{fac}$. The $S_{peak}$ and $\Phi _V$ profiles are independent of $G_{syn}$. **Left column.** $V$ peak profiles. **Middle-left column.** $V$ peak-to-trough amplitude profiles. **Middle-right column.** $S$ peak profiles. **Right column.** $V$ phase profiles. We used Eq. (65) for the PSP $V$ with $I_{syn}$ described by Eqs. (2)-(4) appropriately adapted to account for the translation of $V$ to the equilibrium point, and STP described by Eqs. (12) and (13) (DA model). The impedance amplitude ($Z$) and phase ($\Phi _Z$) were computed using Eqs. (39) and (40). The analytical approximations for the PSP peak sequence response of passive cells to presynaptic inputs are described in Section 2.2.4 (see also Appendix A). The approximation of $V_{peak,n}$, $V_{trough,n}$ and $t_{V,peak}$ were computed as described in Section 2.2.4. The PSP amplitude $\Gamma _V$ was computed by using Eq. (41) and the PSP phase $\Phi _V$ was computed using Eq. (42). The synaptic ($S$) peak ($S_{peak}$) and phase ($\Phi _S$) profiles were computed similarly to these for $V$. We used the following additional parameter values: $C = 1$, $E_L = -60$, $I_{app} = 0$, $G_{syn} = 0.05$, $E_{syn} = 0$, $a_d = 0.1$, $a_f = 0.1$, $x_{\infty } = 1$, $z_{\infty } = 0$ and $T_{sw} = 1$

The presence of STF generates $S_{peak}$ HPFs that become sharper and more amplified as $\tau _{fac}$ increases (Fig. 12-A3) and are independent of $\tau$ and $G_{syn}$ (Fig. 12-B3 to -D3).

#### Modulation of $V_{peak}$ HPFs: interplay of STF- and PSP SUM-mediated HPFs

In the absence of STP, the $V_{peak}$ HPF is controlled by the membrane time constant $\tau$ (Fig. 9-B1) and the synaptic decay time constant $\tau _{dec}$ (Fig. 9-C1). Here we focus on the effects of synaptic facilitation ($\tau _{fac}$) and the interplay between the three time constants in the modulation of $V_{peak}$. Increasing values of $\tau _{fac}$ cause an amplification of the $S_{peak}$ profiles (Fig. 12-A3) and therefore an amplification of the $V_{peak}$ profiles (Fig. 12-A1). Increasing values of $\tau _{dec}$ and $G_{syn}$ increase $I_{syn}$ in a frequency-dependent manner, and therefore they also amplify the $V_{peak}$ profiles (Fig. 12-C1 and -D1). Finally, consistently with our findings in Fig. 8-A, increasing values of $\tau$ also amplify the $V_{peak}$ profiles.

#### Emergence of $\Gamma _V$ resonance (BPFs): interplay of a STF-mediated HPF and a PSP SUM-mediated amplitude LPF

The presence of synaptic STF generates $S_{peak}$ HPFs that become sharper and more amplified as $\tau _{fac}$ increases (Fig. 12-A3). The interaction between these filters and the PSP amplitude LPFs (Fig. 9-A1, light-blue) produces BPFs (Fig. 11-A2). We refer to this preferred frequency PSP amplitude response to periodic presynaptic inputs as (PSP) $\Gamma _V$ resonance. Similarly to other resonances, $\Gamma _V$ resonance reflects balances between the two participating process. As $\tau _{fac}$ increases, the $\Gamma _V$ profiles are more dominated by facilitation and therefore the $\Gamma _V$ profiles are amplified as the $\Gamma _V$ resonant frequency decreases (Fig. 12-A2).

As $\tau$ increases, the STP-independent $\Gamma _V$ LPFs are amplified and sharpened (Fig. 9-B1). Therefore, increasing values of $\tau$ amplify the $\Gamma _V$ BPFs and shift the resonant frequency to lower values (Fig. 11-B2). Similarly, increasing values of $\tau _{dec}$, which sharpen the STP-independent $\Gamma _V$ LPFs (Fig. 9-C1), shift the facilitation-induced $\Gamma _V$ resonant frequency to lower values and amplifies the $\Gamma _V$ BPFs within certain range of values of $\tau$ (Fig. 12-C2). Increasing values of $G_{syn}$ in contrast amplify the $\Gamma _V$ profile with a much lesser effect on the $\Gamma _V$ resonant frequency (Fig. 12-D2)

#### Modulation of the $\Phi _V$

In the absence of STP, the $\Phi _V$ profile is controlled by $\tau$ and $\tau _{dec}$ (Fig. 9). Similarly to our findings in the previous section, changes in $\tau _{fac}$ do not affect $\Phi _V$ (Fig. 12-A4 and S8). Changes in $\tau$ and $\tau _{dec}$ affect $\Phi _V$ in the same direction as in the absence of STP (Figs. 12-B4, -A4, S9 and S10 ). Consistently with the previous findings, changes in $G_{syn}$ do not affect $\Phi _V$ (Fig. 12-D4).

Figures S8-S10 extend our results for additional parameter combinations. Figures S8-S10 (middle-left column), in particular, illustrate how the balances between the facilitation HPFs and amplitude LPFs shape the $\Gamma _V$ profiles for additional parameter regimes.

### Interplay of STD-mediated LPFs, STF-mediated HPFs and PSP SUM-mediated filters HPFs: $V_{peak}$ and $\Gamma _V$ BPFs

**Fig. 13:**
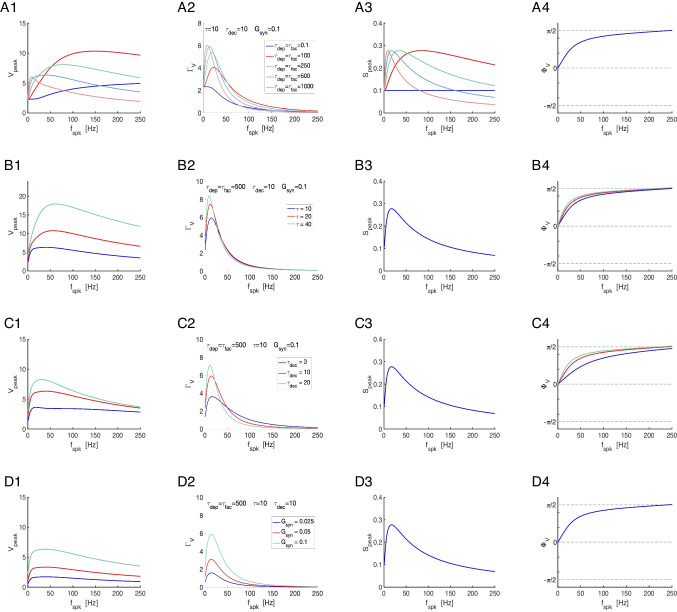
Postsynaptic filters in response to periodic presynaptic spike inputs emerging from the interplay of short-term depression, facilitation and postsynaptic summation. **A.** Superimposed filters for representative values of the depression and facilitation time constants $\tau _{dep}$ and $\tau _{fac}$, respectively, and representative parameter values: $\tau = 10$, $\tau _{dec} = 10$ and $G_{syn} = 0.1$. Figure S11 extends these results for additional values of $\tau$. The $\Phi _V$ profiles are independent of $\tau _{dep}$ and $\tau _{fac}$. **B.** Superimposed filters for representative values of the depression membrane time constant $\tau$ and representative parameter values: $\tau _{dep} = 500$, $\tau _{fac} = 500$, $\tau _{dec} = 10$ and $G_{syn} = 0.1$. Figure S12 extends these results for additional values of $\tau$. The $S_{peak}$ profiles are independent of $\tau$. **C.** Superimposed filters for representative values of the depression synaptic decay time $\tau _{dec}$ and representative parameter values: $\tau _{dep} = 500$, $\tau _{fac} = 500$, $\tau = 10$ and $G_{syn} = 0.1$. Figure S13 extends these results for additional values of $\tau$. The $S_{peak}$ profiles are independent of $\tau$. **D.** Superimposed filters for representative values of the synaptic decay time constant $G_{syn}$ and representative parameter values: $\tau _{dep}=\tau _{fac} = 500$, $\tau = 10$, $\tau _{dec} = 10$. Figure S10 extends these results fo **Left column.** $V$ peak profiles. **Middle-left column.** $V$ peak-to-trough amplitude profiles. **Middle-right column.** $S$ peak profiles. **Right column.** $V$ phase profiles. We used Eq. (65) for the PSP $V$ with $I_{syn}$ described by Eqs. (2)-(4) appropriately adapted to account for the translation of $V$ to the equilibrium point, and STP described by Eqs. (12) and (13) (DA model). The impedance amplitude ($Z$) and phase ($\Phi _Z$) were computed using Eqs. (39) and (40). The analytical approximations for the PSP peak sequence response of passive cells to presynaptic inputs are described in Section 2.2.4 (see also Appendix A). The approximation of $V_{peak,n}$, $V_{trough,n}$ and $t_{V,peak}$ were computed as described in Section 2.2.4. The PSP amplitude $\Gamma _V$ was computed by using Eq. (41) and the PSP phase $\Phi _V$ was computed using Eq. (42). The synaptic ($S$) peak ($S_{peak}$) and phase ($\Phi _S$) profiles were computed similarly to these for $V$. We used the following additional parameter values: $C = 1$, $E_L = -60$, $I_{app} = 0$, $E_{syn} = 0$, $a_d = 0.1$, $a_f = 0.1$, $x_{\infty } = 1$, $z_{\infty } = 0$ and $T_{sw} = 1$

The presence of synaptic STD and STF generates $S_{peak}$ BPFs (synaptic resonance) that become sharper as $\tau _{dep}$ and $\tau _{fac}$ increase (Fig. 13-A3). This is accompanied by a decrease in the synaptic resonance frequency. The $S_{peak}$ BPFs are independent of $\tau$, $\tau _{dec}$ and $G_{syn}$ (Fig. 13-B3 to -D3). As discussed above, for small enough values of $\tau _{dec}$ and $\tau$, the $S_{peak}$ profiles are inherited to the postsynaptic level and therefore the PSP $V_{peak}$ and $\Gamma _V$ profiles are almost identical, and are almost proportional to the $S_{peak}$ profiles (not shown). For larger values of $\tau _{dec}$ and $\tau$, the PSP $V_{peak}$ and $\Gamma _V$ profiles are modulated by the PSP membrane properties and the SUM-mediated HPF.

#### Modulation of $V_{peak}$ BPFs

Earlier studies modeled the PSP response of cells to periodic presynaptic spike inputs ($V_{peak}$ profiles) in the presence of STP to be proportional to the $S_{peak}$ profiles (Markram et al., 1998, 1998b, a). For larger, more realistic values of $\tau$ and $\tau _{dec}$ the $V_{peak}$ profiles are wider than the $S_{peak}$ profiles (compare Figs. 13-A1 and -A3) and the $V_{peak}$ resonant frequency is larger than the $S_{peak}$ resonant frequency. The $V_{peak}$ profiles are amplified by decreasing values of $\tau _{dep}$ and $\tau _{fac}$. The amplification is stronger as $\tau$ increases (Fig. S11, column 1). The $V_{peak}$ amplification is accompanied by an increase in the $V_{peak}$ resonant frequency as $\tau _{dep}$ and $\tau _{fac}$ decrease. The $V_{peak}$ profiles are also amplified by increasing values of $\tau$, $\tau _{dec}$ and $G_{syn}$ (Figs. 13, column 1). Increasing values of $\tau$ and $\tau _{dec}$ cause an increase in the $V_{peak}$ resonant frequency (Figs. 13-B1 and -C1, see also Figs. S12 and S13, column 1), but the $V_{peak}$ resonant frequency is at most slightly affected by increasing values of $G_{syn}$ (Figs. 13-D1).

#### Modulation of $\Gamma _V$ BPFs

The $\Gamma _V$ profiles are amplified and sharpened by increasing values of $\tau _{dep}$ and $\tau _{fac}$ (Fig. 13-A2). This is more pronounced as $\tau$ increases (Fig. S11, column 2). This is accompanied by a decrease in the $\Gamma _V$ resonant frequency. The $\Gamma _V$ profiles are also amplified by increasing values of $\tau$, $\tau _{dec}$ and $G_{syn}$. In the former two cases they are also sharpened.

#### Modulation of the $\Phi _V$

In the absence of STP, the $\Phi _V$ profile is controlled by $\tau$ and $\tau _{dec}$ (Fig. 9). Consistent with our findings in the previous two sections, changes in $\tau _{dep}$ and $\tau _{fac}$ do not affect $\Phi _V$ (Fig. 13-A4 and S11). Changes in $\tau$ and $\tau _{dec}$ affect $\Phi _V$ in the same direction as in the absence of STP (Figs. 13-B4, -A4, S11 and S12 ). Consistent with the previous findings, changes in $G_{syn}$ do not affect $\Phi _V$ (Figs. 13-D4)

Figures S11-S13 extend our results for additional parameter combinations.

### Persistence and modulation of STP-mediated PSP $V_{peak}$ and $\Gamma _V$ BPFs in response to randomly-distributed spike trains

In the previous sections we used periodic presynaptic inputs over a range of spiking frequencies to describe a number of mechanisms of generation of STP-mediated PSP $V_{peak}$ and $\Gamma _V$ BPFs both inherited from the $S$ level of organization ($S_{peak}$ BPFs) and generated by filters of opposing types across levels of organization.

The question arises whether PSP $V_{peak}$ and $\Gamma _V$ filters emerge in more general scenarios, in response to more realistic presynaptic spike trains having some frequency content, what are the properties of these filters, how they are affected by the input variability, and how they are related to the classical (deterministic) filters in response to periodic inputs. We address these questions by using two types of presynaptic spike inputs: jittered-periodic spike trains (Mondal et al., 2022) and Poisson-distributed spike trains (Dayan & Abbott, 2001; Gerstner et al., 2014).

#### PSP $V_{peak}$ and $\Gamma _V$ BPFs persist in response to randomly perturbed periodic spike trains

Following Mondal et al. (2022), we consider perturbations of periodic presynaptic spiking patterns with ISIs of the form (6) for $n = 1, \ldots , N_{spk}$, where $\Delta _{spk}$ is constant ($n$-independent) and $\delta _p = \{ \delta _{spk,n} \}_{n=1}^{N_{spk}}$ is a random variable with zero mean and variance equal to $\delta \, \Delta _{spk}$ for a non-negative real number $\delta$.

**Fig. 14:**
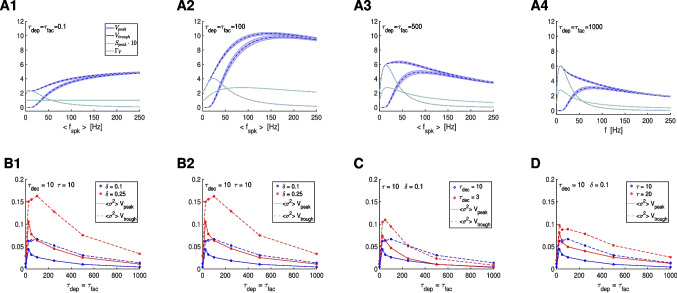
**Postsynaptic filters in response to jittered (randomly perturbed) periodic presynaptic inputs in the presence of STP: frequency- and STP-dependent variability.** For each value of the mean presynaptic input frequency $< f_{spk}>$, the ISI sequence $\{ \Delta _{spk,n} \}$ ($n = 1, \ldots , N_{spk}$) has the form $\Delta _{spk,n} = \Delta _{spk} + \delta _{spk,n}$ where $\Delta _{spk}$ is the ISI corresponding to $f_{spk}$ ($f_{spk} = 1000 / \Delta _{spk}$) and the sequence $\{ \delta _{spk,n} \}$ are drawn from a normal distribution with zero mean and variance equal to $\delta \, \Delta _{spk}$. **A.** Superimposed $V_{peak}$, $V_{trough}$, $S_{peak}$ and $\Gamma _V$ profiles for representative parameter values. We used $\tau _{dec} = 10$ and $\tau = 10$ in all panels. Solid curves correspond to the mean values for each attribute ( $V_{peak}$, $V_{trough}$, $S_{peak}$ and $\Gamma _V$). The shadow regions ( $V_{peak}$, $V_{trough}$ and $S_{peak}$ correspond to one standard deviation from the mean. The dashed gray curves, almost coinciding with the solid curves, represent the corresponding deterministic profiles (response to periodic spike train inputs with frequency $f_{spk}$). **A1.** $\tau _{dep} = \tau _{fac} = 0.1$. **A2.** $\tau _{dep} = \tau _{fac} = 100$. **A3.** $\tau _{dep} = \tau _{fac} = 500$. **A4.** $\tau _{dep} = \tau _{fac} = 1000$. **B.** Averaged variances for the $V_{peak}$ (solid) and $V_{trough}$ (dashed) profiles as a function of $\tau _{dep} = \tau _{fac}$ for representative parameter values. The average variance for each profile was computed by averaging the corresponding response variances across all values of $< f_{spk}>$. **B.** Increasing the input variability ($\delta$) increases the response variability. Panels B1 and B2 show two realizations for the same parameter values. We used $\tau = 10$ and $\tau _{dec} = 10$. **C.** Decreasing the synaptic decay time $\tau _{dec}$ causes an increase in the response variability. We used $\tau = 10$ and $\delta = 0.1$. **D.** Increasing the membrane potential time constant causes an increase in the response variability. We used $\tau _{dec} = 10$ and $\delta = 0.1$. We used the following additional parameter values: $C = 1$, $E_L = -60$, $I_{app} = 0$, $E_{syn} = 0$, $a_d = 0.1$, $a_f = 0.1$, $x_{\infty } = 1$, $z_{\infty } = 0$ and $T_{sw} = 1$

Figure 14-A illustrates that PSP $V_{peak}$ and $\Gamma _V$ filters discussed above persist in response to the jittered-periodic presynaptic spike trains. The solid curves correspond to the mean value for each attribute ($V_{peak}$, $V_{trough}$, $S_{peak}$ and $\Gamma _V$) and the dashed-gray curves correspond to the classical, unperturbed filters. Figure 14-A illustrates that these two quantities almost coincide. This also occurs for the $X_{peak}$, $Z_{peak}$ and $\Delta S_{peak}$ filters ( Fig. S16). The variability of the filters is larger for $V_{peak}$ and $V_{trough}$ than for the other filters, and is frequency-dependent (compare the shadow regions for each filter across frequencies) and STP-dependent (compare the shadow regions for each input frequency across values of $\tau _{dep}$ and $\tau _{fac}$).

#### PSP $V_{peak}$ and $\Gamma _V$ BPFs persist in response to Poisson-distributed spike trains and are modulated by these inputs

We use Poisson-distributed spike trains with stationary mean firing rates $r_{spk}$ ($<f_{spk}>$) within the same range as the spiking input frequencies used above. For comparison with the previously discussed cases, we identify the mean firing rate with the spiking frequency $f_{spk}$ from which it originates.

**Fig. 15:**
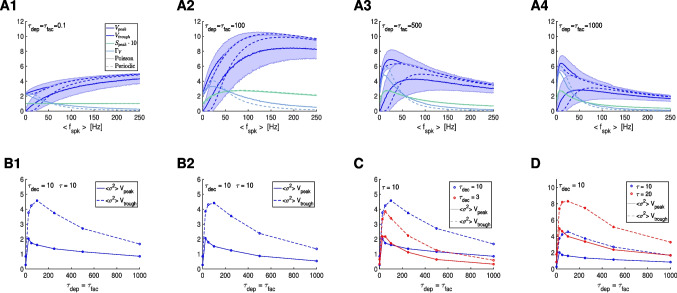
**Postsynaptic filters in response to Poisson-distributed presynaptic inputs in the presence of STP: frequency- and STP-dependent variability.** The mean rate of the Poisson distributed spike trains corresponds to $< f_{spk}>$. **A.** Superimposed $V_{peak}$, $V_{trough}$, $S_{peak}$ and $\Gamma _V$ profiles for representative parameter values. We used $\tau _{dec} = 10$ and $\tau = 10$ in all panels. Solid curves correspond to the mean values for each attribute ( $V_{peak}$, $V_{trough}$, $S_{peak}$ and $\Gamma _V$). The shadow regions ( $V_{peak}$, $V_{trough}$ and $S_{peak}$ correspond to one standard deviation from the mean. The dashed curves represent the corresponding deterministic profiles (response to periodic spike train inputs with frequency $f_{spk}$). **A1.** $\tau _{dep} = \tau _{fac} = 0.1$. **A2.** $\tau _{dep} = \tau _{fac} = 100$. **A3.** $\tau _{dep} = \tau _{fac} = 500$. **A4.** $\tau _{dep} = \tau _{fac} = 1000$. **B.** Averaged variances for the $V_{peak}$ (solid) and $V_{trough}$ (dashed) profiles as a function of $\tau _{dep} = \tau _{fac}$ for representative parameter values. The average variance for each profile was computed by averaging the corresponding response variances across all values of $< f_{spk}>$. **B.** Two realizations for the same parameter values. We used $\tau = 10$ and $\tau _{dec} = 10$. **C.** Decreasing the synaptic decay time $\tau _{dec}$ causes a decrease in the response variability for large values of $<f_{spk}>$ and an increase in the response variability for (very) small values of $f_{spk}$. We used $\tau = 10$ and $\delta = 0.1$. **D.** Increasing the membrane potential time constant causes an increase in the response variability. We used $\tau _{dec} = 10$ and $\delta = 0.1$. We used the following additional parameter values: $C = 1$, $E_L = -60$, $I_{app} = 0$, $E_{syn} = 0$, $a_d = 0.1$, $a_f = 0.1$, $x_{\infty } = 1$, $z_{\infty } = 0$ and $T_{sw} = 1$

Figure 15-A illustrates that PSP $V_{peak}$ and $\Gamma _V$ filters persists in response to Poisson-distributed presynaptic inputs, but the perturbations from the classical filters (in response to periodic inputs) are more prominent than in the case discussed above (the solid and dashed curves do not coincide and they are significantly further apart). As expected, the response variability to Poisson inputs is larger than for jittered-periodic inputs (compare the shadow regions in Figs. 14-A and 15-A and Figs. S16-A and S17-A). Similarly to the filters discussed above, the variability is larger for the $V_{peak}$ and $V_{trough}$ filters than for the other filters, is frequency-dependent (compare the shadow regions for each filter across frequencies), and STP-dependent (compare the shadow regions for each input frequency across values of $\tau _{dep}$ and $\tau _{fac}$.

### STP controls the variability of PSP $V_{peak}$ and $\Gamma _V$ BPFs in response to jittered-periodic and Poisson distributed spike trains

A salient feature of the frequency filters presented in Figs. 14-A and 15-A is the dependence of the response variability (shadow regions), not only with the mean input frequency (or rate), but also with the time constants controlling the short-term depression and facilitation processes (for the same input frequencies).

We quantified these dependencies in Figs. 14- and 15-B, -C and D for values of $\tau _{dep} = \tau _{fac}$ within a representative range for the $V_{peak}$ and $V_{trough}$ profiles. For each value of $\tau _{dep} = \tau _{fac}$ we computed the average variance for each profile (across input frequencies). We obtained similar results by using the maximal variance for each profile.

In all cases, the variability first increases as $\tau _{dep} = \tau _{fac}$ increase for relative small values of these parameters, and then it decreases as $\tau _{dep} = \tau _{fac}$ continues to increase. For each set of time constants ($\tau _{dec}$ and $\tau$), there is a value of $\tau _{dep} = \tau _{fac}$ for which the variability is maximal. As expected, for the jittered periodic inputs the profiles’ variability increases as the input variability increases (Figs. 14- B). The profiles’ variability also increases as $\tau _{dec}$ decreases (Figs. 14- C) and $\tau$ increases (Figs. 14- D). For the Poisson-distributed inputs, in contrast, the profiles’ variability decreases with decreasing values of $\tau _{dec}$ (Fig. 15-C), while it increases for increasing values of $\tau$ (Fig. 15-D).

Together, these results and the results of the previous section show that STP plays important roles not only in determining the patterns exhibit by networks, but also their robustness and accuracy of the information transmission.

## Discussion

Neuronal filters play important roles in neuronal communication, information selection and processing, neuronal oscillations, neuronal resonance and brain computations (Hutcheon & Yarom, 2000; Tsodyks et al., 2000; Izhikevich et al., 2003; Stark et al., 2022; Laudansky et al., 2014; Akam & Kullmann, 2012; Maass, 2016; Beiran & Ostojic, 2019; Fortune & Rose, 2001; Klyachko & Stevens, 2006; Thomson, 2003; Akam & Kullmann, 2010; Blankenburg et al., 2015; Rosenbaum et al., 2012; Brunel et al., 2001; Buonomano & Maass, 2009; Lisman, 1997; Sherfey et al., 2018). Neuronal filters emerge as the result of a variety of mechanisms that involve a multiplicity of time scales associated to the neuronal electric circuit and biophysical properties and history-dependent processes. In spite of it ubiquitousness, the mechanisms of generation of neuronal filters are poorly understood beyond the single cell level. In previous work (Stark et al., 2022), we showed that band-pass filters (BPFs) can be inherited across levels of neuronal organization or can be created independently at various levels of organization by the interplay of low-pass filters (LPFs) and high-pass filters (HPFs) belonging to the same or different levels (e.g., schematic diagrams in Fig. 2). However, the mechanism by which these BPFs, LPFs and HPFs are generated, interact and are modulated as they transition across levels of organization are not clear.

We set out to address these issues in an elementary network motif (Fig. 1). We investigated the biophysical and dynamic mechanisms of generation of postsynaptic (PSP) filters in response to presynaptic spike inputs in the presence of synaptic short-term plasticity (STP). We were particularly interested in understanding how PSP filters are shaped by the time scales associated with the participating building blocks: the synaptic rise and decay dynamics ($\tau _{rse}$ and $\tau _{dec}$), the synaptic STP time constants ($\tau _{dep}$ and $\tau _{fac}$), and the intrinsic time constant ($\tau$) of the postsynaptic cells (Fig. 1). To this end, we conducted a systematic study of the steady-state PSP responses to (i) periodic presynaptic inputs over a range of frequencies ($f_{spk} = 1000 / \Delta _{spk}$; Fig. 1-A, top), (ii) jittered-periodic inputs over a range of mean frequencies ($f_{spk} = 1000 / \Delta _{spk}$), and (iii) Poisson-distributed presynaptic inputs over a range of mean rates ($r_{spk} = 1000 / <\Delta _{spk}>$; Fig. 1-A, bottom).

The use of periodic presynaptic spike trains allowed us to systematically understand the basic mechanistic aspects governing the generation of frequency filters in the elementary network motif: (i) how frequency-filters at the different levels of organization are shaped by the time scales of the participating building blocks, (ii) how the filtering properties are communicated across levels of neuronal organization, and (iii) how filters interact across levels of neuronal organization. The use of Poisson-distributed presynaptic spikes trains allowed us to extend our investigation and findings to more realistic scenarios and to explore how the variability of the filtering properties is controlled by the biophysical properties and time scales of the participating building blocks, primarily STP. The jittered-periodic inputs were used as an intermediate step to link between the purely deterministic and stochastic spike-train inputs.

We used a combination of mathematical modeling, analytical calculations and numerical simulations. As part of our study, we developed reduced models and analytical approximations of the membrane potential response of passive cells to presynaptic spike trains in the presence of STP. This allowed us to compute the peak ($V_{peak}$) and peak-to-trough amplitude ($\Gamma _V$) filters in terms of the participating time constants and other model parameters. While we could have conducted this study by using solely numerical simulations, obtaining analytical expressions provided us with a better understanding of the dependence of the PSP filters on the model parameters and time scales. This was achieved at the expense of some simplifying assumptions that transform the multiplicative synaptic input to the passive membrane equation into an sequentially-corrected additive input where the additive synaptic input was corrected at the arrival of each presynaptic spike to account for the membrane potential changes during the presynaptic interspike interval. The resulting expressions make the contribution of the time constants and other parameters to the PSP filters’s shape apparent. One could argue that the sequentially-corrected reduced model was not necessary since the passive membrane equation is linear, and the response to multiplicative inputs is analytically solvable (e.g., by using Laplace transforms). However, the complex formula describing the exact analytical solution fails to provide the level of clarity and intuition desired to understand how the model parameters shape the PSP filters. While we consider here the sequentially-corrected reduced model, Eqs. (25)-(27), as an approximation to the more detailed biophysical model, we note that it could stand as a model of their own (Levenstein et al., 2023). The approach we used in this paper can be extended to more complex scenarios such as networks where the presynaptic or postsynaptic cells are described by more complex linearized models involving additional ionic currents and weakly nonlinear models (Chialva et al., 2023).

The role of STP on information filtering and related phenomena has been investigated before by many authors (Dittman et al., 2000; Silberberg et al., 2004; Markram et al., 1998a; Fortune & Rose, 2000, 2001, 1997a, b, 2002; Thomson, 2003; Goldman et al., 2002; Mejias & Torres, 2008; Bourjaily & Miller, 2012; Klyachko & Stevens, 2006; George et al., 2011; Lewis & Maler, 2002; Kandaswamy et al., 2010; Varela et al., 1997; Chance et al., 1998; Zador & Dobrunz, 1997; Lisman, 1997; Izhikevich et al., 2003; Buonomano, 2000; Zucker & Regehr, 2002; Pouille & Scanziani, 2004; Gabernet et al., 2005; Tsodyks & Markram, 1996, 1997; Rotman et al., 2011; Tauffer & Kumar, 2021; Markram et al., 1998; Tsodyks et al., 1998). However, previous studies have not focused on the mechanisms of generation of PSP frequency-filters and how they are shaped by the participating time scales. Previous work has also ignored the mechanisms governing the variability of the response to realistic presynaptic spike-train inputs. Because the synaptic response $S$ is not directly measurable, a common simplifying assumption has been made by some authors (Markram et al., 1998, 1998a; Tsodyks & Markram, 1997; Tsodyks et al., 1998): that the voltage response of the postsynaptic cell is a scaled version of the synaptic response. While this assumption might be justified in many cases, our results indicate that it is by no mean universally expected, and there could be significant qualitative differences between the synaptic and postsynaptic frequency-filters. This was also highlighted in previous work on temporal filters in the presence of STP (Mondal et al., 2022).

We divided our study in three steps. We investigated the response profiles (frequency-filters) of (i) the synaptic update $\Delta S$ to the presynaptic spike trains (the target of the synaptic variable $S$), (ii) the synaptic variable $S$ through the synaptic update $\Delta S$, and (iii) the postsynaptic membrane potential $V$ to the synaptic variable $S$. We characterized the frequency-filters by using the (stationary) peak profiles (for $\bar{\Delta S}$, $\bar{S}$ and $\bar{V}$) and the (stationary) peak-to-trough amplitude profiles ($\Gamma _{S}$ and $\Gamma _{V}$). By design, the effects of STP are present at the $\Delta S$ level giving rise to the synaptic update sequences $\Delta S_n = X_n Z_n$, which depend on the time constants $\tau _{dep}$ and $\tau _{fac}$ and on $f_{spk}$ (the $\bar{\Delta S}$ filter is steady-state of these sequences). These are the target of the synaptic variables $S$ during the rise phase immediately after the arrival of each presynaptic spike. The $S$ frequency-filters are shaped by $\Delta S_n = X_n Z_n$ and the synaptic time constantes $\tau _{rse}$ and $\tau _{dec}$. In turn, the synaptic variable $S$ is the input to the current-balance equation describing the dynamics of the passive cell. The PSP frequency-filters result from the interaction between $S$ and the biophysical properties of the postsynaptic passive cell, particularly the time constant $\tau = C / G_L$.

Consistently with previous work, Markram et al. (1998, 1998a); Tsodyks and Markram (1997); Tsodyks et al. (1998); Izhikevich et al. (2003); Drover et al. (2007), $\bar{\Delta } S$ BPFs are generated by the interplay of low-pass (depression) and high-pass (facilitation) filters for the appropriate balances between the two processes (e.g., Fig. 2-A). They are amplified by increasing values of $\tau _{fac}$ and the $\bar{\Delta } S$ resonant frequency decreases as $\tau _{fac}$ increases. The $\bar{X}$, $\bar{Z}$ and $\bar{\Delta } S$ profiles develop in response to multiple events, each controlled by the time constants $\tau _{dep}$ and $\tau _{fac}$. We described the (global in time) filter properties in terms of the characteristic frequencies $\sigma _{dep}$ ($\bar{X}$), $\sigma _{fac}$ ($\bar{Z}$), and the characteristic frequency difference $\Delta _{kappa} = \kappa _{rse}$ and $\kappa _{dec}$ ($\bar{\Delta S}$). These depend on $\tau _{dep}$ and $\tau _{fac}$ in a nonlinear manner. Increasing values of $\tau _{fac}$ and $\tau _{dep}$ cause $\sigma _{fac}$ and $\sigma _{dep}$, respectively, to decrease (sharpen the $\bar{X}$, $\bar{Z}$ filters, respectively). Increasing values of $\tau _{fac}$ cause the $\bar{\Delta } S$ resonant frequency to decrease, the $\bar{\Delta } S$ peak to increase and the $\bar{\Delta } S$ BPF to become sharper. Increasing values of $\tau _{dep}$ cause the $\bar{\Delta } S$ resonant frequency to decrease and the $\bar{\Delta } S$ BPF to become sharper, but cause the the $\bar{\Delta } S$ peak to decrease.

For the to-$\bar{\Delta S}$ update model with instantaneous $S$ rise, the peak envelope profiles $\bar{S}$ are identical to the $\bar{\Delta S}$ profiles. The $\Gamma _S$ BPFs can be inherited from $\bar{\Delta S}$ ones or can be created by the interplay of a $\bar{\Delta S}$ HPF and $Q_A$ (a LPF). In all cases, they become sharper and less peakier as $\tau _{dec}$ increases. For the to-$\bar{\Delta S}$ update model with non instantaneous $S$ rise, the $\bar{S}$ profiles are attenuated as $\tau _{rse}$ increases and the $\Gamma _S$ profiles are also attenuated as $\tau _{rse}$ increases.

For the by-$\bar{\Delta S}$ update model with instantaneous $S$ rise, in contrast to the to-$\bar{\Delta S}$ model, the $\Gamma _S$ profiles are inherited from the $\bar{\Delta S}$ profiles. The $\bar{S}$ profiles transition from LPFs or BPFs to HPFs as $\tau _{dec}$ increases, which may be bounded or unbounded (if they are HPFs, they remain so). These models lack a biophysical mechanism that balances the summation effects and created realistically saturated profiles. For the by-$\bar{\Delta S}$ update model with non-instantaneous $S$ rise, the $\bar{S}$ profiles are attenuated as $\tau _{rse}$ increases and the $\Gamma _S$ profiles are also attenuated as $\tau _{rse}$ increases.

For linear systems in response to sinusoidal inputs, amplitude and membrane potential peak filters coincide. This is no longer true for nonlinear systems or neuronal systems receiving presynaptic inputs. Therefore, we investigated the two types of filters ($V_{peak}$ and $\Gamma _V$). Passive cells exhibit LPFs in response to sinusoidal input currents. In contrast, they exhibit $V_{peak}$ HPFs in response to periodic presynaptic inputs in the absence of STP, while still exhibiting $\Gamma _V$ LPFs. These filters are modulated by the synaptic time constant $\tau _{dec}$ and the postsynaptic time constant $\tau$. In the presence of STP, $V_{peak}$ and $\Gamma _V$ BPFs are possible under certain conditions. We found two qualitatively different mechanisms. The $V_{peak}$ and $\Gamma _V$ BPFs can be either inherited from the synaptic level, subject to the appropriate modulations, or generated across levels. Specifically, a $V_{peak}$ BPF can be either result of an $S_{peak}$ BPF generated as the result of synaptic depression and facilitation or the result of a $S_{peak}$ LPF generated by the interplay of synaptic depression and PSP summation. Similarly, a $\Gamma _V$ BPF can be the result of the interplay of synaptic facilitation and PSP summation.

These types of BPFs persist in response to jitter periodic spike trains and Poisson-distributed spike trains, exhibiting a frequency-dependent variability. Importantly, the variability properties of these BPFs are controlled by STP in a frequency-dependent manner. This highlights a role of STP as regulating the variability of PSP filters and creating variability filters. To our knowledge, this has not been described before and requires a systematic study.

The results of this study complement previous work (Mondal et al., 2022) where we thoroughly investigated the temporal (transient) responses and the associated temporal filters in the same feedforward network motif (Fig. 1). One important result of this study was the description of the link between the STP time constants governing the dynamics of the single events (in response to each presynaptic spikes; $\tau _{dep}$ and $\tau _{fac}$) and the corresponding global, emergent time scales describing the long-term dynamics of the (low-, high- and band-pass) temporal filters. A second important result was the discovery of a third global time scale for temporal band-pass filters involving a combination of both $\tau _{dep}$ and $\tau _{fac}$, highlighting the complexity of the non-trivial interaction between depression and facilitation. A third important result was the finding that the postsynaptic temporal filters are not proportional to the synaptic temporal filters as assumed in some simplified models, Markram et al. (1998), thus demonstrating that the synaptic temporal filters are not directly communicated to the postsynaptic cell level, but rather modified by the postsynaptic intrinsic properties. However, temporal filters are not necessarily predictive of frequency-filters and both types of filters are generated by different mechanisms.

The feedforward network motif we studied (Fig. 1) is arguably the basic network processing unit that can produce STP-dependent PSP BPFs. This allow us to systematically investigate the qualitatively different types of mechanisms of generation of PSP BPFs according to whether they are inherited from the synaptic level or generated across levels of organization. These mechanisms are expected to operate in larger and more complex networks of which the basic network motif we studied here becomes a building block. Testing these ideas requires extending our work to include presynaptic BPFs and presynaptic patterns generated by oscillatory input currents to the presynaptic cell. It would be natural to hypothesize the existence of inherited PSP BPFs in the absence of STP and PSP BPFs generated by cross-level mechanisms including the presynaptic level. Further research is needed to understand these scenarios and to understand more complex network motifs including additional connections (e.g., feedback connections both excitatory and inhibitory) in the presence of STP in one or all of them and combinations of the network motif studied here. Additional research should focus on the modulation of STP-dependent PSP filters by astrocytes, which are known to regulate synaptic depression and facilitation (De Pitta et al., 2012). Additional research is also needed to understand the mechanisms of regulation of PSP filters’ variability by STP. Finally, future work should focus on more complex scenarios and more complex biophysically realistic models of STP where STP is determined by the combined effect of both pre- and post-synaptic factors (Losonczy et al., 2002) (see also Markram et al., 1998; Gupta et al., 2000; Reyes et al., 1998). Complex scenarios should include the effects of background noise, neuronal noise and spiking irregularities (Brunel & Hakim, 2008; Brunel, 2000; Brunel et al., 2001; Fourcaud-Trocme et al., 2003; Fourcaud & Brunel, 2002).

In this context, our study is a first step in the systematic understanding of the mechanisms of generation of STP-mediated neuronal filters in networks and contributes to the systematic understanding of information processing via neuronal filters in neuronal systems.

## Supplementary Information

Below is the link to the electronic supplementary material.

Supplementary file 1 (pdf 4902 KB)

## Data Availability

The codes are available at https://github.com/BioDatanamics-Lab/Frequency_Filters_STP_21_06.

## A: Approximate analytical solution to the passive postsynaptic cell receiving presynaptic spike inputs

Here we compute an analytical approximate solution to the model (1)-(4), describing the dynamics of a postsynaptic passive cell receiving presynaptic spike inputs at time $t_1, t_2, \ldots$.

### A.1: Approximate solution to the synaptic variable

We first compute the approximate solution to the synaptic variable $S$ whose dynamics are described by Eq. (3) with $S(0) = 0$, under the assumption of instantaneous rise to a value $\Delta S_n$ ($n=1, 2\, \ldots$) at the arrival of each presynaptic spike. The assumption $S(0) = 0$ implies that $S(t) = 0$ for $0 \le t \le t_1$. For the duration of a presynaptic spike ($t_n< t < t_n + T_{sw}$), we approximate $S(t)$ by the synaptic update value $\Delta S_n$ (constant). For the remainder of the presynaptic interspike interval ($t_n +T_{sw} \le t < t_{n+1}$), $S(t)$ decreases according to the second term in Eq. (3). The approximate solution is given by

74 $$\begin{aligned} S_a(t) = \left\{ \begin{array}{llll} \Delta S_n & & & t_n< t< t_n + T_{sw} \\ \\ \Delta S_n\, e^{-(t-t_n-T_{sw})/\tau _{dec}} & & & t_n +T_{sw} \le t < t_{n+1} \\ \end{array} \right. \end{aligned}$$

for $$n = 1, 2, \ldots$$.

From Eq. (74) for $S_a(t)$, the larger the duration $T_{sw}$ of the presynaptic spike ($T_{sw} < \Delta _{spk}$) or the larger the synaptic decay time $\tau _{dec}$, the larger $S_a(t)$ during the presynaptic ISI. In that sense, increasing $\tau _{dec}$ or $T_{sw}$ has a similar overall effect on the PSP response as increasing $G_{syn}$. However, changes in the former two have stronger dynamic effects (e.g., cause changes in the voltage response peak) than changes in $G_{syn}$ (Fig. 8).

### A.2: Approximate solution to the postsynaptic membrane potential: Postsynaptic passive cell

We first approximate Eq. (1) as follows

75 $$\begin{aligned} \tau \, \frac{dV}{dt} = - V + \alpha _n\, S_a(t) \end{aligned}$$

for $$n = 1, \ldots , N$$ where

76 $$\begin{aligned} \tau = \frac{C}{G_L}, \hspace{.5cm} \alpha = \frac{G_{syn}\, (E_{syn}-E_L)}{G_L} \hspace{.5cm} \text{ and } \hspace{.5cm} \alpha _n = \alpha \, (1-\sigma _n). \end{aligned}$$

The variable $V$ in Eq. (75) represents $V - E_L - I_{app}/G_L$ in Eq. (1). The third term in Eq. (75) is a current input approximation to the conductance input in the synaptic current (1). To account for this, we introduce a correction factor $1 - \sigma _n$ where $\sigma _n$ is updated at the beginning of each ISI and $\sigma _1 = 0$.

Prior to the arrival of the first presynaptic spike ($t \le t_1$), $V(t) = 0$. For the duration of a presynaptic spike ($t_n< t < t_n + T_{sw}$),

77 $$\begin{aligned} \tau \, \frac{dV}{dt} = - V + \alpha _n\, \Delta S_n, \hspace{2cm} V(t_n) = V_{0,n}, \end{aligned}$$

where $V_{0,n}$ is the value of $V$ at the end of the previous cycle. The solution to Eq. (77) is given by

78 $$\begin{aligned} V_I(t) = \alpha _n\, \Delta S_n + (V_{o,n} - \alpha _n\, \Delta S_n)\, e^{-(t-t_n)/\tau }. \end{aligned}$$

We define

79 $$\begin{aligned} \beta _n = V_I(t_n+T_{sw}) = \alpha _n\, \Delta S_n + (V_{o,n} - \alpha _n\, \Delta S_n)\, e^{-T_{sw}/\tau }. \end{aligned}$$

For the remainder of the presynaptic interspike interval ($t_n + T_{sw}< t < t_{n+1}$),

80 $$\begin{aligned} \tau \, \frac{dV}{dt} = - V + \alpha _n\, \Delta S_n\, e^{-(t-t_n-T_{sw})/\tau } \hspace{1.6cm} V(t_n+T_{sw}) = \beta _n, \end{aligned}$$

For $\tau _{dec} \ne \tau$, the solution to Eq. (80) is given by

81 $$\begin{aligned} V_{II}(t)&= \frac{\alpha _n\, \tau _{dec}\, \Delta S_n}{\tau _{dec}-\tau }\, e^{T{sw}/\tau _{dec}}\, e^{-(t-t_n)/\tau _{dec}} \nonumber \\&\quad + \left[ \, \beta _n - \frac{\alpha _n\, \tau _{dec}\, \Delta S_n}{\tau _{dec}-\tau }\, \right] \, e^{T_{sw}/\tau }\, e^{-(t-t_n)/\tau }. \end{aligned}$$

We define

82 $$\begin{aligned} V_{0,n+1}&= V_{II}(t_{n+1}) = \frac{\alpha _n\, \tau _{dec}\, \Delta S_n}{\tau _{dec}-\tau }\, e^{T_{sw}/\tau _{dec}}\, e^{-(t_{n+1}-t_n)/\tau _{dec}} \nonumber \\&\quad + \left[ \, \beta _n - \frac{\alpha _n\, \tau _{dec}\, \Delta S_n}{\tau _{dec}-\tau }\, \right] \, e^{T_{sw}/\tau }\, e^{-(t_{n+1}-t_n)/\tau } = \nonumber \\&= \frac{\alpha _n\, \tau _{dec}\, \Delta S_n}{\tau _{dec}-\tau }\, e^{T_{sw}/\tau _{dec}}\, e^{-\Delta _{spk,n}/\tau _{dec}} \nonumber \\&\quad + \left[ \, \beta _n - \frac{\alpha _n\, \tau _{dec}\, \Delta S_n}{\tau _{dec}-\tau }\, \right] \, e^{T_{sw}/\tau }\, e^{-\Delta _{spk,n}/\tau }. \end{aligned}$$

For the duration of the presynaptic spike, $V(t) = V_I(t)$ is an increasing function. $V(t) = V_{II}(t)$ continues to be an increasing function after the presynaptic spike is off until the two exponential terms balance out and $V_{II}(t)$ decreases. Therefore, within some frequency range, $V(t)$ peaks after the presynaptic spike is off. The peak time for $V_{II}(t)$ in Eq. (81) is given

83 $$\begin{aligned} t_{peak,n} = t_n + \frac{\tau _{dec}\, \tau }{\tau _{dec}-\tau }\, \ln \left( -\frac{b_n\, \tau _{dec}}{a_n\, \tau } \right) \end{aligned}$$

where

84 $$\begin{array}{ccc}a_n=\frac{\alpha_n\,\tau_{dec}\,\Delta S_n}{\tau_{dec}-\tau}\,e^{T_{sw}/\tau_{dec}}{}&\mathrm{and}&b_n=\left[\,\beta_n-\frac{\alpha_n\,\tau_{dec}\,\Delta S_n}{\tau_{dec}-\tau}\,\right]\,e^{T_{sw}/\tau}.\end{array}$$

The peak value of $V(t)$ is given by

85 $$\begin{aligned} V_{peak,n} = V_{II}(t_{peak,n}). \end{aligned}$$

We define $\sigma _{n+1}$ as

86 $$\begin{aligned} \sigma _{n+1} = \frac{\eta \, V_{peak,n} + (1-\eta )\, V_{0,n+1}}{(E_{syn}-E_L)}. \end{aligned}$$

or

87 $$\begin{aligned} \sigma _{n+1} = \frac{\eta \, V_{peak,n} }{(E_{syn}-E_L)}. \end{aligned}$$

For $\tau _{dec} = \tau$, the solution to Eq. (80) is given by

88 $$\begin{aligned} V_{II}(t)&= \frac{\alpha _n\, \Delta S_n}{\tau }\, e^{T_{sw}/\tau }\, t\, e^{-(t-t_n)/\tau } \nonumber \\&\quad + \left[ \, \beta _n - \frac{\alpha _n\, \Delta S_n}{\tau }\, (t_n+T_{sw})\, \right] \, e^{T_{sw}/\tau }\, e^{-(t-t_n)/\tau }. \end{aligned}$$

The peak time for $V_{II}(t)$ in Eq. (88) is given

89 $$\begin{aligned} t_{peak,n} = t_n + \tau - \frac{b_n}{a_n} \end{aligned}$$

where

90 $$\begin{array}{ccc}a_n=\frac{\alpha_n\,\Delta S_n}\tau\,e^{T_{sw}/\tau_{dec}}{}&\mathrm{and}&b_n=\left[\,\beta_n-\frac{\alpha_n\,\Delta S_n}\tau\,T_{sw}\,\right]\,e^{T_{sw}/\tau_{dec}}.\end{array}$$

The peak values of $V(t)$ is given by Eq. (85) with $\beta _n$ given by Eq. (79) and $V_{0,n+1} = V_{II}(t_{n+1})$

## B: Tables

**Table 1:** Acronyms

STP	Short-term plasticity
STD	Short-term depression
STF	Short-term facilitation
PSP	Post-synaptic potentials
LPF	Low-pass filter
HPF	High-pass filter
BPF	Band-pass filter
ISI	Interspike interval
SUM	Summation
DA	Dayan-Abbott (model)
MT	Markram-Tsodyks (model)

**Table 2:** Metrics

Metrics	
$Z$	Subthreshold impedance amplitude
$\Phi _Z$	Subthreshold impedance phase
$\bar{X}$	Depression peak steady state (DA)
$\bar{Z}$	Facilitation peak steady state (DA)
$\bar{\Delta S} = \bar{X}\, \bar{Z}$	Synaptic update steady state (DA)
$\hat{X}$	Rescaled ^1^$\bar{X}$
$\hat{Z}$	Rescaled ^1^$\bar{Z}$
$\bar{R}$	Depression peak steady state (MT)
$\bar{u}$	Facilitation peak steady state (MT)
$\bar{S}_{peak}$	$S$ peak steady state
$\bar{S}_{trough}$	$S$ trough steady state
$\Gamma _S =\bar{S}_{peak}-\bar{S}_{trough}$	$\bar{S}$ peak-to-trough amplitude
$\bar{V}_{peak}$	PSP peak steady-state
$\bar{V}_{trough}$	PSP peak steady-state
$t_{peak,V}$	$\bar{V}_{peak}$ time
$\Gamma _V$	PSP peak-to-trough amplitude
$\Phi _V = (t_{peak,V}-t_{spk})\, \pi / \Delta _{spk}$ ^2^	PSP phase
M profile	Curve of the metrics M as a function
	of the spiking input frequency $f_{spk}$
M filter (LPF, HPF, BPF)	Represented by the M profile

DA refers to the DA model (Section 2.1.5). MT refers to the MT model (Section 2.1.7). ^1^ See Eq. (44). ^2^$t_{spk}$ is the presynaptic spike time immediately preceding the occurrence of the $V$ peak ($V_{peak}$) at $t_{peak,V}$

**Table 3:** Variables and parameters

$V$	membrane potential	$t$	time
$S$	synaptic variable	$\Delta S$	synaptic update (target value for $S$)
$x$	depression variable (DA)	$z$	facilitation variable (DA)
$R$	depression variable (MT)	$u$	facilitation variable (MT)
$\{ X_{n} \}_{n=1}^{\infty }$	depression peak sequence (DA)	$\{ Z_{n} \}_{n=1}^{\infty }$	facilitation peak sequence (DA)
$\{ \Delta S_n \}_{n=1}^{\infty }$	synaptic update sequence ($= \{X_n Z_n\}$)		
$\{ V_{peak,n} \}_{n=1}^{\infty }$	PSP peak sequence	$\{ V_{trough,n} \}_{n=1}^{\infty }$	PSP trough sequence
$\{ S_{peak,n} \}_{n=1}^{\infty }$	$\bar{S}$ peak sequence	$\{ S_{trough,n} \}_{n=1}^{\infty }$	$\bar{S}$ trough sequence
$\{ R_{n} \}_{n=1}^{\infty }$	depression peak sequence (MT)	$\{ u_{n} \}_{n=1}^{\infty }$	facilitation peak sequence (MT)
$C$	membrane capacitance	$I_{app}$	applied current
$G_L$	maximal leak conductance	$E_L$	leak reversal potential
$G_{syn}$	maximal synaptic conductance ($G_{ex}$)	$E_{syn}$	synaptic reversal potential ($E_{ex}$)
$\tau _{dep}$	synaptic depression time constant	$\tau _{fac}$	synaptic facilitation time constant
$\tau _{rse}$	synaptic rise time	$\tau _{dec}$	synaptic decay time
$\tau = C / G_L$	Membrane potential time constant	$\gamma _{syn}$	= $G_{syn} / C$
$\{t_{spk,n}\}_{n=1}^{\infty }$	spike times sequence ($\{t_n\}_{n=1}^{\infty }$),	$\Delta _{spk,n}$	presynaptic ISI sequence
$r_{spk}$	presynaptic mean rates	$T_{sw}$	presynaptic spike duration
$f_{spk}$	presynaptic spiking frequency	$\Delta _{spk}$	$= 1000 / f_{spk}$ presynaptic ISI
$a_d$	depression update (DA)	$a_f$	facilitation update (DA)
$x_{\infty }$ ($= 1$)	depression saturation (DA)	$z_{\infty }$ ($= 0$)	facilitation saturation (DA)
$\sigma _{dep}$	$\bar{X}$-filter characteristic time	$\sigma _{fac}$	$\bar{Z}$-filter characteristic time
$\Delta S_{max}$	$\bar{\Delta S}$-BPF peak	$f_{\Delta S,res}$	$\bar{\Delta S}$-BPF resonant frequency
$\kappa _{rse}$	$\bar{\Delta S}$-BPF rise frequency	$\kappa _{dec}$	$\bar{\Delta S}$-BPF decay frequency
$U$ ($=1$)	facilitation update (MT)	$\hat{U}$ ($=0$)	facilitation saturation (MT)
$\alpha$	see Eq. (26) [22]	$\alpha _n$	see Eq. (26) [22]
$\sigma _s$	see Eq. (27) [23]	$\delta _{spk,n}$	jitter perturbation sequence
$S_a$	synaptic variable in Eq. (27) [23]	$\eta _{stp}$	see Eq. (43) [38]
$f$	sinusoidal input frequency	$\omega$	$= 2 \pi f / 1000$
$A_{in}$	sinusoidal input amplitude	$\hat{\Delta }_{spk}$	see Eq. (43) [38]
$V_{eq}$	$= E_L + I_{app}/G_L$ in Eq. (26)		

DA refers to the DA model (Section 2.1.5). MT refers to the MT model (Section 2.1.7)
