# Supplementary material for: Postsynaptic frequency filters shaped by the interplay of synaptic short-term plasticity and cellular time scales
Source: J Comput Neurosci. 2025 Oct 21;53(4):551–91. doi: 10.1007/s10827-025-00908-3 (PMC12672824; doi:10.1007/s10827-025-00908-3)
Supplement: Supplementary file 1 — (pdf 4902 KB) [file 10827_2025_908_MOESM1_ESM.pdf]

Supplementary Material

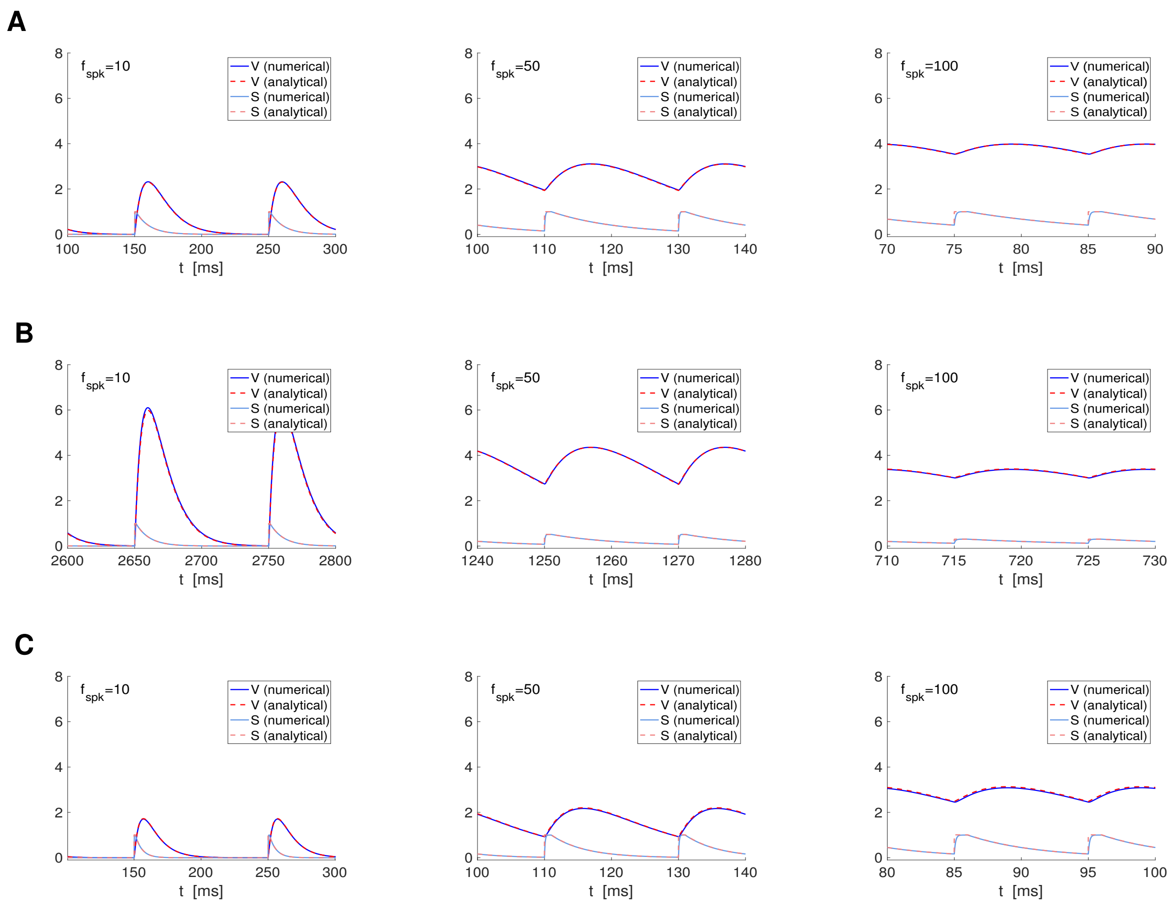

Figure S1: **Analytical approximation of the membrane potential response of passive cells to presynaptic spikes: Representative examples I.** For the numerical approximations we used the model for a passive cell receiving presynaptic spike-train input (1)-(4). For STP we use the DA model (7)-(9). For the analytical approximations we used eqs. (25)-(27) together with eqs. (78) and (81) in the Appendix A. **A.** $G_L = 0.1$ ($\tau = 10$), $\tau_{dec} = 10$, $\tau_{dep} = \tau_{fac} = 0.1$. **B.** $G_L = 0.1$ ($\tau = 10$), $\tau_{dec} = 10$, $\tau_{dep} = \tau_{fac} = 1000$. **C.** $G_L = 0.1$ ($\tau = 10$), $\tau_{dec} = 5$, $\tau_{dep} = \tau_{fac} = 0.1$. We used the following additional parameter values: $a_d = 0.1$, $a_f = 0.1$, $x_\infty = 1$, $z_\infty = 0$, $\tau_{rse} = 0.1$, $C = 1$, $E_L = -60$, $I_{app} = 0$, $G_{syn} = 0.1$, $E_{syn} = -60$.

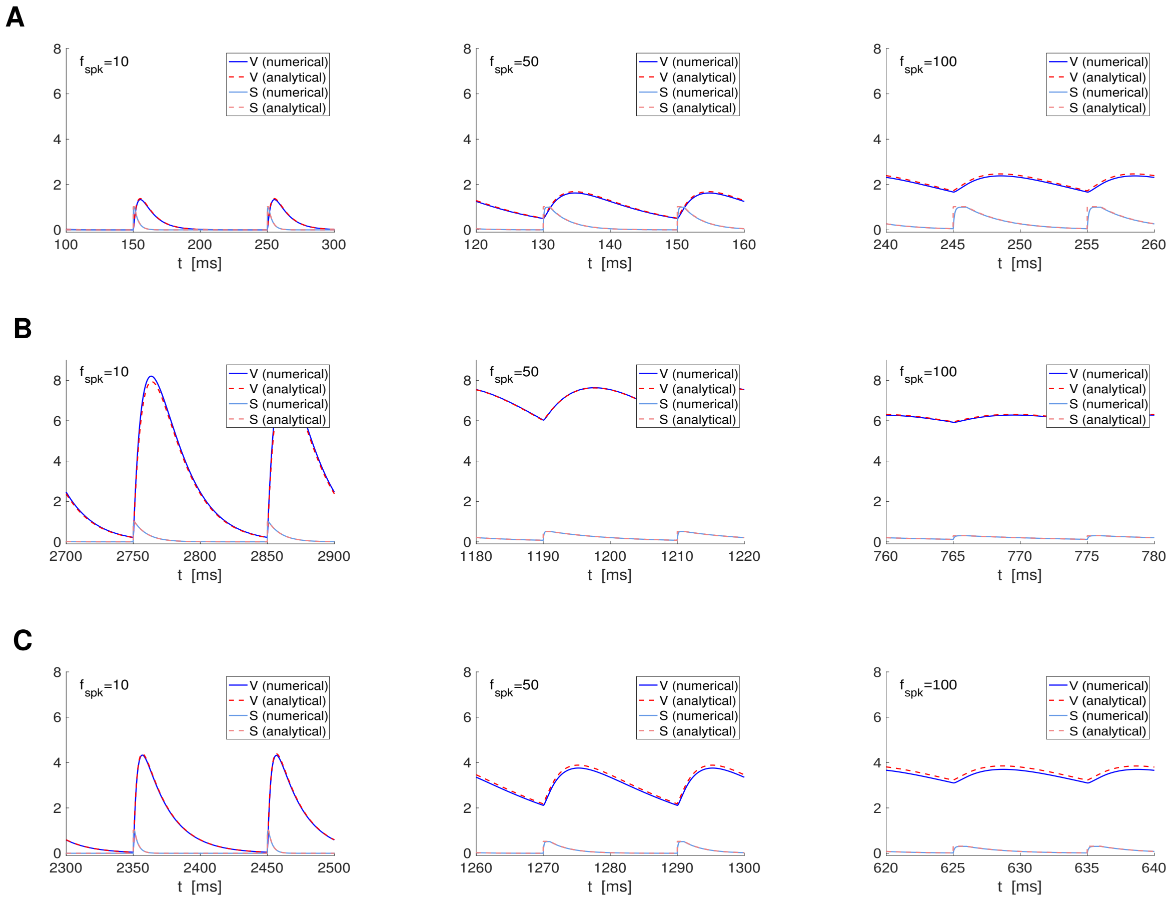

Figure S2: **Analytical approximation of the membrane potential response of passive cells to presynaptic spikes: Representative examples II.** For the numerical approximations we used the model for a passive cell receiving presynaptic spike-train input (1)-(4). For STP we used the DA model (7)-(9). For the analytical approximations we used eqs. (25)-(27) together with eqs. (78) and (81) in the Appendix A. **A.** $G_L = 0.1$ ($\tau = 10$), $\tau_{dec} = 3$, $\tau_{dep} = \tau_{fac} = 0.1$. **B.** $G_L = 0.05$ ($\tau = 20$), $\tau_{dec} = 10$, $\tau_{dep} = \tau_{fac} = 1000$. **C.** $G_L = 0.05$ ($\tau = 20$), $\tau_{dec} = 5$, $\tau_{dep} = \tau_{fac} = 1000$. We used the following additional parameter values: $a_d = 0.1$, $a_f = 0.1$, $x_\infty = 1$, $z_\infty = 0$, $\tau_{rse} = 0.1$, $C = 1$, $E_L = -60$, $I_{app} = 0$, $G_{syn} = 0.1$, $E_{syn} = -60$.

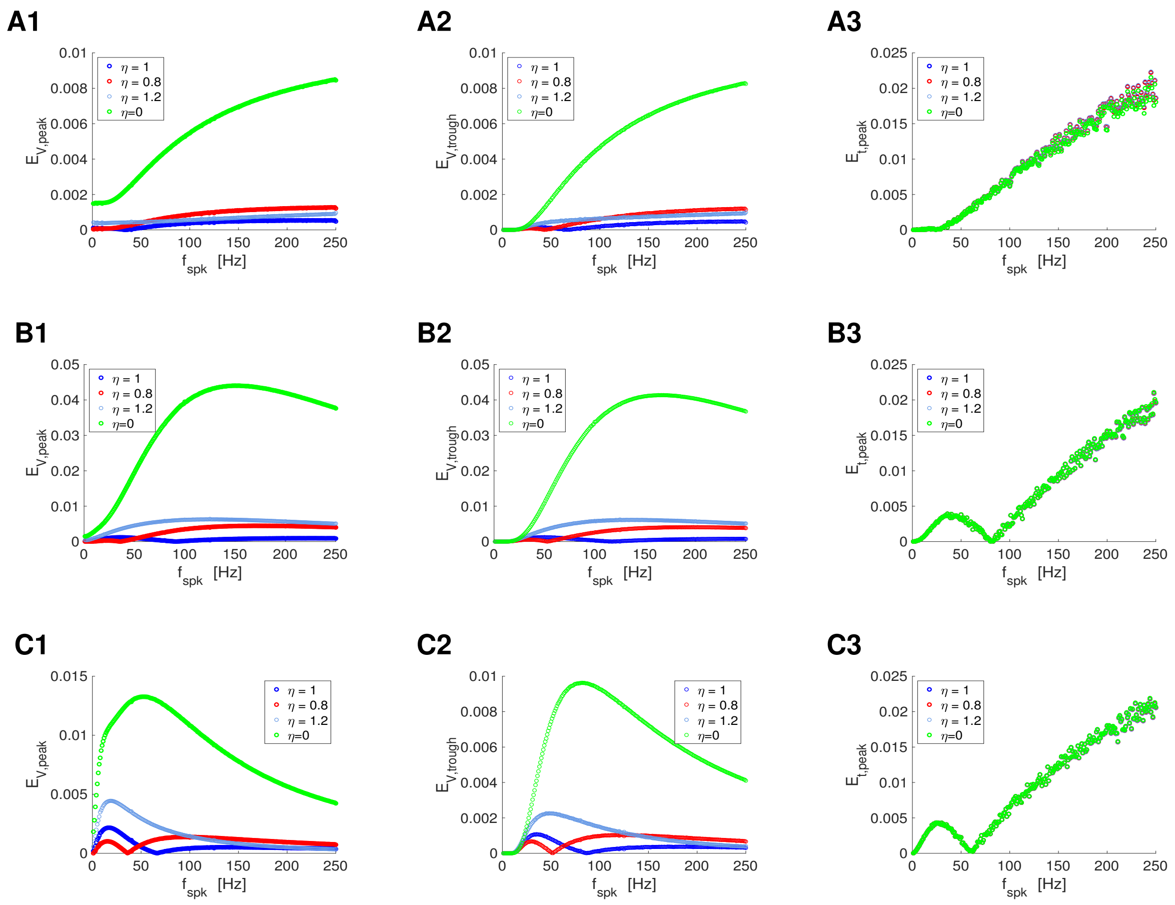

Figure S3: **Error between the numerical and analytical approximations for the stationary peaks (V_{peak}), troughs (V_{trough}), and peak times (t_{peak}) of the membrane potential responses of passive cells to presynaptic spikes: Representative examples I.** For the numerical (num) approximations we used the model for a passive cell receiving presynaptic spike-train input (1)-(4). For STP we used the DA model (7)-(9). For the analytical approximations we used eqs. (25)-(27) together with eqs. (78) and (81) in the Appendix A. For the computations of the analytical (anl) approximations to V_{peak}, V_{trough} and t_{peak} we used eqs. (28)-(35). Simulations were carried out until the difference between two consecutive numerical peaks were below a tolerance value equal to 0.001. The last values of V_{peak}, V_{trough} and t_{peak} in the resulting sequences were taken as an approximation to the corresponding stationary values. **Left column.** Relative error for V_{peak} defined as $\mid V_{peak,num} - 60 - V_{peak,anl} \mid / \mid V_{peak,num} \mid$. **Middle column.** Relative error for V_{trough} defined as $\mid V_{trough,num} - 60 - V_{trough,anl} \mid / \mid V_{trough,num} \mid$. **Right column.** Relative error for t_{peak} defined as $\mid t_{peak,num} - t_{peak,anl} \mid / \mid \Delta_{spk} \mid$. **A.** $G_L = 0.1$ ($\tau = 10$), $\tau_{dec} = 10$, $\tau_{dep} = \tau_{fac} = 0.01$. **B.** $G_L = 0.1$ ($\tau = 10$), $\tau_{dec} = 5$, $\tau_{dep} = \tau_{fac} = 100$. **C.** $G_L = 0.1$ ($\tau = 10$), $\tau_{dec} = 10$, $\tau_{dep} = \tau_{fac} = 500$. We used the following additional parameter values: $a_d = 0.1$, $a_f = 0.1$, $x_\infty = 1$, $z_\infty = 0$, $\tau_{rse} = 0.1$, $C = 1$, $E_L = -60$, $I_{app} = 0$, $G_{syn} = 0.1$, $E_{syn} = -60$, $\Delta t = 0.01$.

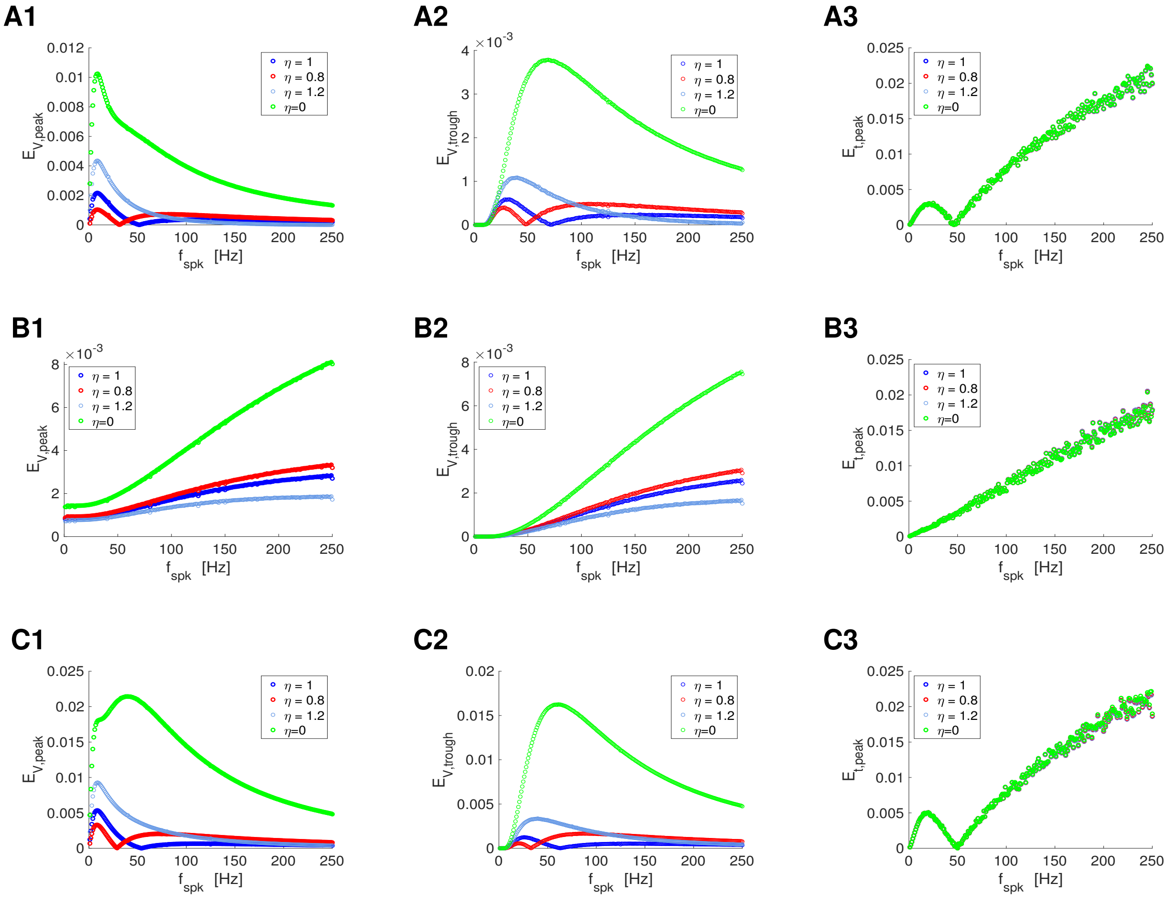

Figure S4: **Error between the numerical and analytical approximations for the stationary peaks (V_{peak}), troughs (V_{trough}), and peak times (t_{peak}) of the membrane potential responses of passive cells to presynaptic spikes: Representative examples II.** For the numerical (num) approximations we used the model for a passive cell receiving presynaptic spike-train input (1)-(4). For STP we used the DA model (7)-(9). For the analytical approximations we used eqs. (25)-(27) together with eqs. (78) and (81) in the Appendix A. For the computations of the analytical (anl) approximations to V_{peak}, V_{trough} and t_{peak} we used eqs. (28)-(35). Simulations were carried out until the difference between two consecutive numerical peaks were below a tolerance value equal to 0.001. The last values of V_{peak}, V_{trough} and t_{peak} in the resulting sequences were taken as an approximation to the corresponding stationary values. **Left column.** Relative error for V_{peak} defined as $|\, V_{peak,num} - 60 - V_{peak,anl} \,|/|\, V_{peak,num} \,|$. **Middle column.** Relative error for V_{trough} defined as $|\, V_{trough,num} - 60 - V_{trough,anl} \,|/|\, V_{trough,num} \,|$. **Right column.** Relative error for t_{peak} defined as $|\, t_{peak,num} - t_{peak,anl} \,|/|\, \Delta_{spk} \,|$. **A.** $G_L = 0.1$ ($\tau = 10$), $\tau_{dec} = 10$, $\tau_{dep} = \tau_{fac} = 1000$. **B.** $G_L = 0.1$ ($\tau = 10$), $\tau_{dec} = 3$, $\tau_{dep} = \tau_{fac} = 0.01$. **C.** $G_L = 0.05$ ($\tau = 20$), $\tau_{dec} = 10$, $\tau_{dep} = \tau_{fac} = 1000$. We used the following additional parameter values: $a_d = 0.1$, $a_f = 0.1$, $x_\infty = 1$, $z_\infty = 0$, $\tau_{rse} = 0.1$, $C = 1$, $E_L = -60$, $I_{app} = 0$, $G_{syn} = 0.1$, $E_{syn} = -60$, $\Delta t = 0.01$.

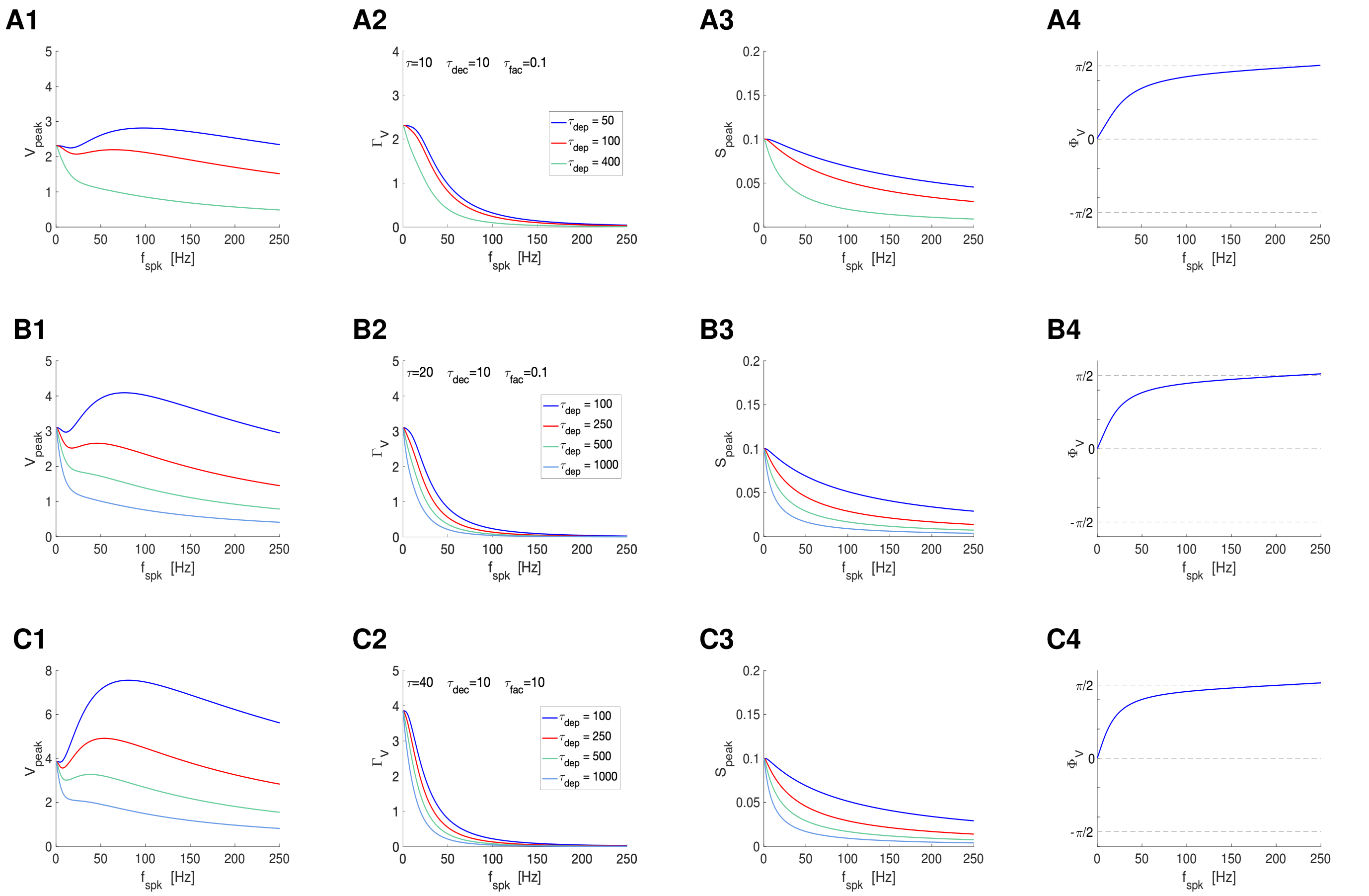

Figure S5: **Postsynaptic filters in response to periodic presynaptic spike inputs emerging from the interplay of short-term depression and postsynaptic summation.** Superimposed filters for representative values of the short-term depression time constant τ_{dep}. **A.** $\tau = 10$. **B.** $\tau = 20$. **C.** $\tau = 40$. **A, B, C.** $\tau_{dec} = 10$ and $\tau_{fac} = 0.1$. **Left column.** V peak profiles. **Middle-left column.** V peak-to-trough amplitude profiles. **Middle-right column.** S peak profiles. **Right column.** V phase profiles. We used eq. (65) for the PSP V with I_{syn} described by eqs. (2)-(4) appropriately adapted to account for the translation of V to the equilibrium point, and STP described by eqs. (12) and (13) (DA model). The impedance amplitude (Z) and phase (Φ_Z) were computed using eqs. (39) and (40). The analytical approximations for the PSP peak sequence response of passive cells to presynaptic inputs are described in Section 2.2.4 (see also Appendix A). The approximation of $V_{peak,n}$, $V_{trough,n}$ and $t_{V,peak}$ were computed as described in Section 2.2.4. The PSP amplitude Γ_V was computed by using eq. (41) and the PSP phase Φ_V was computed using eq. (42). The synaptic (S) peak (S_{peak}) and phase (Φ_S) profiles were computed similarly to these for V. We used the following additional parameter values: $C = 1$, $E_L = -60$, $I_{app} = 0$, $G_{syn} = 0.1$, $E_{syn} = 0$, $a_d = 0.1$, $a_f = 0.1$, $x_\infty = 1$, $z_\infty = 0$ and $T_{sw} = 1$.

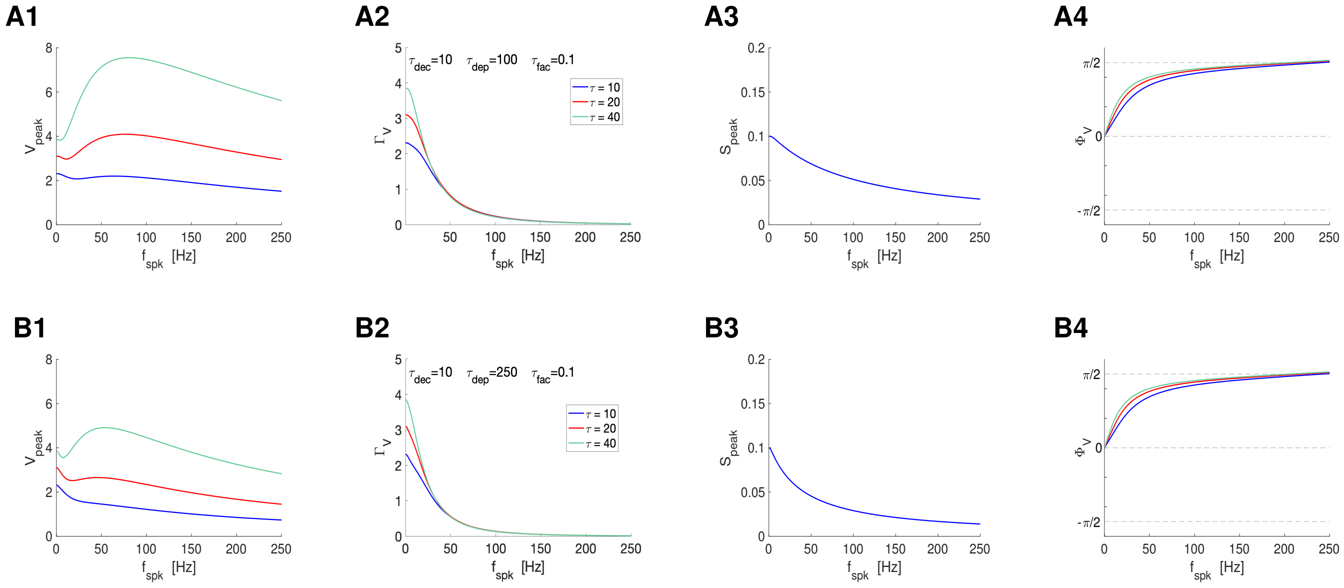

Figure S6: **Postsynaptic filters in response to periodic presynaptic spike inputs emerging from the interplay of short-term depression and postsynaptic summation.** Superimposed filters for representative values of the membrane time constant τ. **A.** $\tau_{dep} = 100$. **B.** $\tau_{dep} = 250$. **A, B** $\tau_{dec} = 10$ and $\tau_{fac} = 0.1$. **Left column.** V peak profiles. **Middle-left column.** V peak-to-trough amplitude profiles. **Middle-right column.** S peak profiles. They are independent of τ. **Right column.** V phase profiles. We used eq. (65) for the PSP V with I_{syn} described by eqs. (2)-(4) appropriately adapted to account for the translation of V to the equilibrium point, and STP described by eqs. (12) and (13) (DA model). The impedance amplitude (Z) and phase (Φ_Z) were computed using eqs. (39) and (40). The analytical approximations for the PSP peak sequence response of passive cells to presynaptic inputs are described in Section 2.2.4 (see also Appendix A). The approximation of $V_{peak,n}$, $V_{trough,n}$ and $t_{V,peak}$ were computed as described in Section 2.2.4. The PSP amplitude Γ_V was computed by using eq. (41) and the PSP phase Φ_V was computed using eq. (42). The synaptic (S) peak (S_{peak}) and phase (Φ_S) profiles were computed similarly to these for V. We used the following additional parameter values: $C = 1$, $E_L = -60$, $I_{app} = 0$, $G_{syn} = 0.1$, $E_{syn} = 0$, $a_d = 0.1$, $a_f = 0.1$, $x_\infty = 1$, $z_\infty = 0$ and $T_{sw} = 1$.

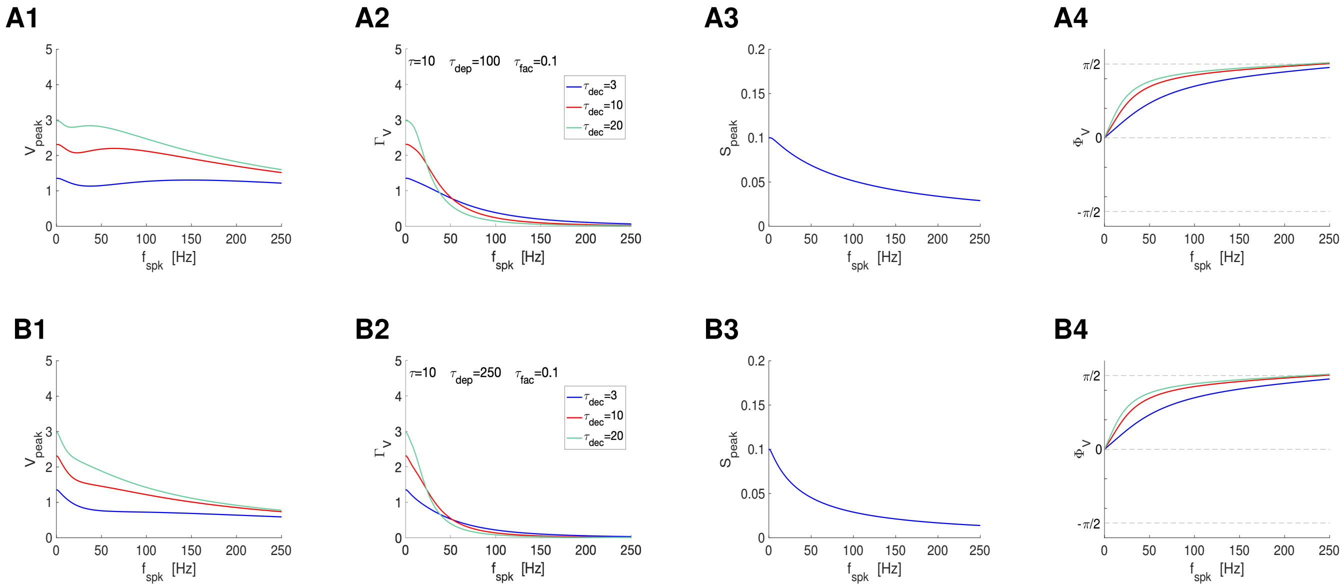

Figure S7: **Postsynaptic filters in response to periodic presynaptic spike inputs emerging from the interplay of short-term depression and postsynaptic summation.** Superimposed filters for representative values of the synaptic decay time constant τ_{dec}. **A.** $\tau_{dep} = 100$. **B.** $\tau_{dep} = 150$. **A, B.** $\tau = 10$ and $\tau_{fac} = 0.1$. **Left column.** V peak profiles. **Middle-left column.** V peak-to-trough amplitude profiles. **Middle-right column.** S peak profiles. They are independent of τ_{dec}. **Right column.** V phase profiles. We used eq. (65) for the PSP V with I_{syn} described by eqs. (2)-(4) appropriately adapted to account for the translation of V to the equilibrium point, and STP described by eqs. (12) and (13) (DA model). The impedance amplitude (Z) and phase (Φ_Z) were computed using eqs. (39) and (40). The analytical approximations for the PSP peak sequence response of passive cells to presynaptic inputs are described in Section 2.2.4 (see also Appendix A). The approximation of $V_{peak,n}$, $V_{trough,n}$ and $t_{V,peak}$ were computed as described in Section 2.2.4. The PSP amplitude Γ_V was computed by using eq. (41) and the PSP phase Φ_V was computed using eq. (42). The synaptic (S) peak (S_{peak}) and phase (Φ_S) profiles were computed similarly to these for V. We used the following additional parameter values: $C = 1$, $E_L = -60$, $I_{app} = 0$, $G_{syn} = 0.1$, $E_{syn} = 0$, $a_d = 0.1$, $a_f = 0.1$, $x_\infty = 1$, $z_\infty = 0$ and $T_{sw} = 1$.

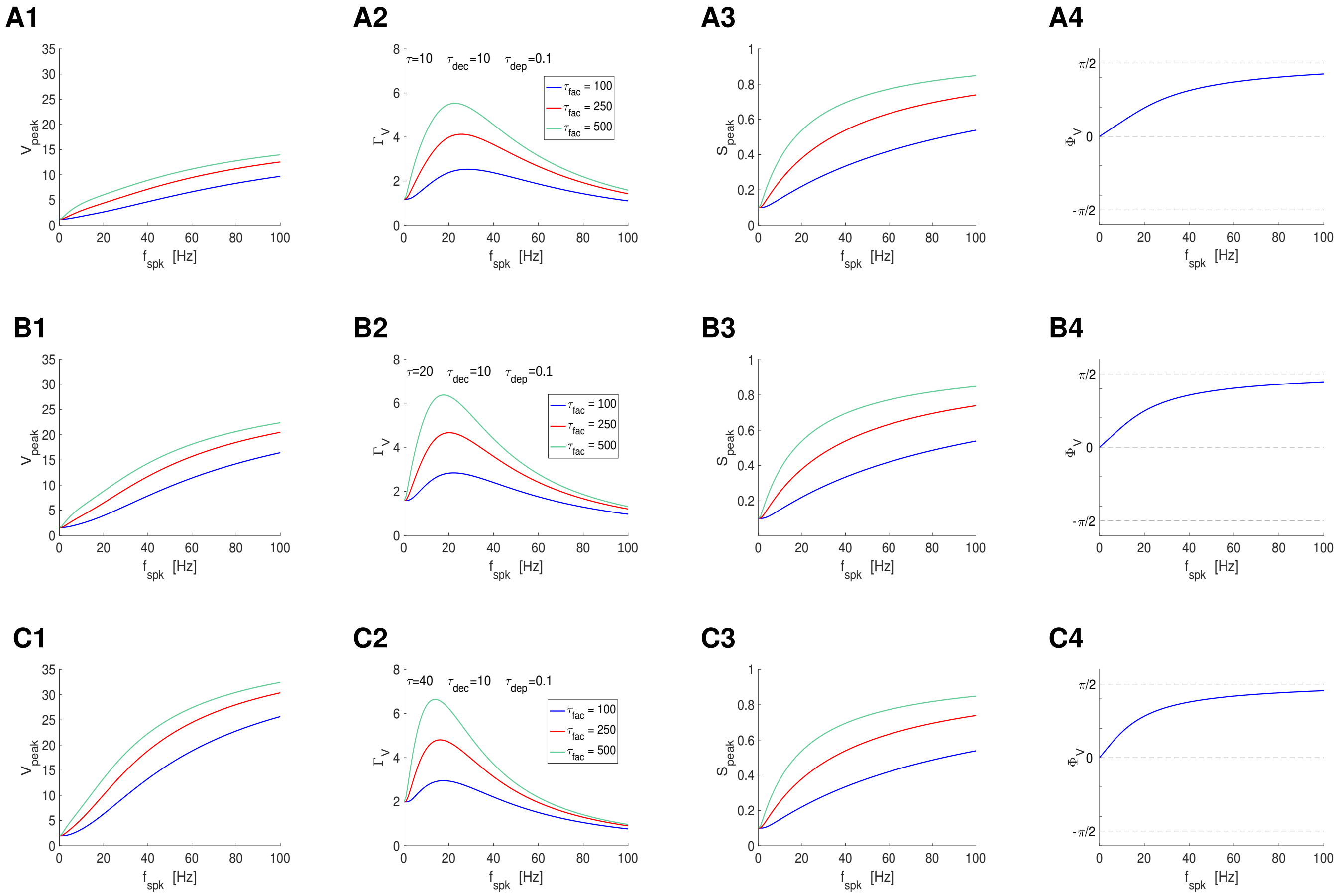

Figure S8: **Postsynaptic filters in response to periodic presynaptic spike inputs emerging from the interplay of short-term facilitation and postsynaptic summation.** Superimposed filters for representative values of the short-term facilitation time constant τ_{fac}. **A.** $\tau = 10$. **B.** $\tau = 20$. **C.** $\tau = 40$. **A, B, C.** $\tau_{dec} = 10$ and $\tau_{dep} = 0.1$. **Left column.** V peak profiles. **Middle-left column.** V peak-to-trough amplitude profiles. **Middle-right column.** S peak profiles. **Right column.** V phase profiles. We used eq. (65) for the PSP V with I_{syn} described by eqs. (2)-(4) appropriately adapted to account for the translation of V to the equilibrium point, and STP described by eqs. (12) and (13) (DA model). The impedance amplitude (Z) and phase (Φ_Z) were computed using eqs. (39) and (40). The analytical approximations for the PSP peak sequence response of passive cells to presynaptic inputs are described in Section 2.2.4 (see also Appendix A). The approximation of $V_{peak,n}$, $V_{trough,n}$ and $t_{V,peak}$ were computed as described in Section 2.2.4. The PSP amplitude Γ_V was computed by using eq. (41) and the PSP phase Φ_V was computed using eq. (42). The synaptic (S) peak (S_{peak}) and phase (Φ_S) profiles were computed similarly to these for V. We used the following additional parameter values: $C = 1$, $E_L = -60$, $I_{app} = 0$, $G_{syn} = 0.05$, $E_{syn} = 0$, $a_d = 0.1$, $a_f = 0.1$, $x_\infty = 1$, $z_\infty = 0$ and $T_{sw} = 1$.

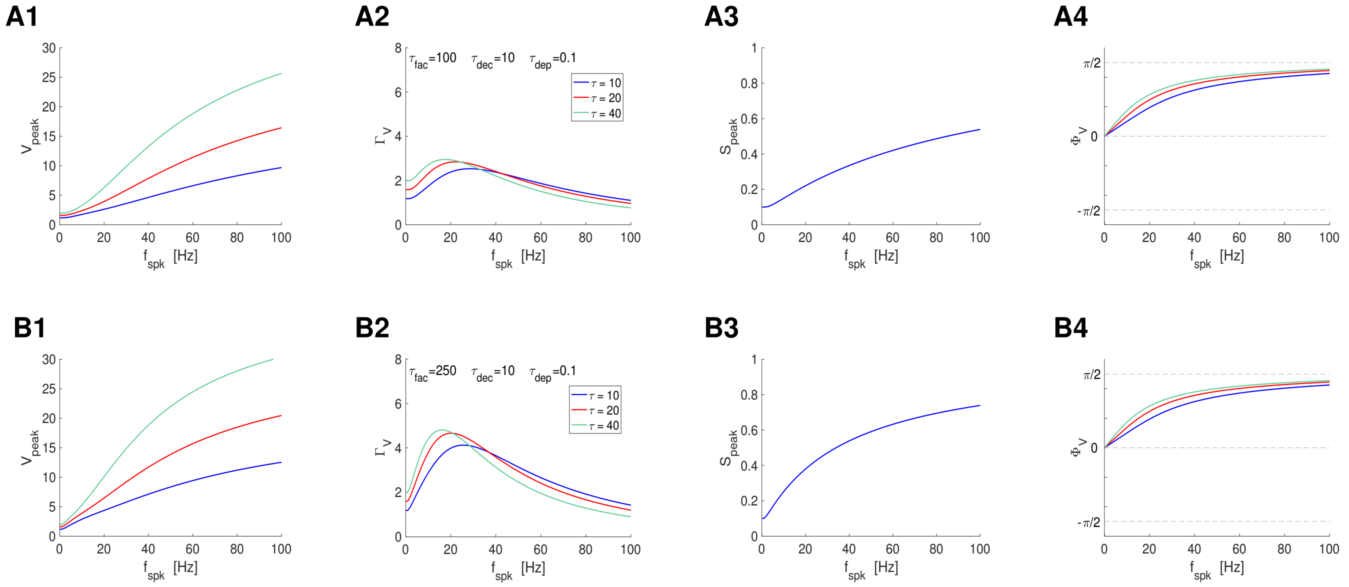

Figure S9: **Postsynaptic filters in response to periodic presynaptic spike inputs emerging from the interplay of short-term facilitation and postsynaptic summation.** Superimposed filters for various values of the membrane time constant τ. **A.** $\tau_{fac} = 100$. **B.** $\tau_{fac} = 250$. **A, B, C.** $\tau_{dec} = 10$ and $\tau_{dep} = 0.1$. **Middle-left column.** V peak-to-trough amplitude profiles. **Middle-right column.** S peak profiles. **Right column.** V phase profiles. We used eq. (65) for the PSP V with I_{syn} described by eqs. (2)-(4) appropriately adapted to account for the translation of V to the equilibrium point, and STP described by eqs. (12) and (13) (DA model). The impedance amplitude (Z) and phase (Φ_Z) were computed using eqs. (39) and (40). The analytical approximations for the PSP peak sequence response of passive cells to presynaptic inputs are described in Section 2.2.4 (see also Appendix A). The approximation of $V_{peak,n}$, $V_{trough,n}$ and $t_{V,peak}$ were computed as described in Section 2.2.4. The PSP amplitude Γ_V was computed by using eq. (41) and the PSP phase Φ_V was computed using eq. (42). The synaptic (S) peak (S_{peak}) and phase (Φ_S) profiles were computed similarly to these for V. We used the following additional parameter values: $C = 1$, $E_L = -60$, $I_{app} = 0$, $G_{syn} = 0.1$, $E_{syn} = 0$, $a_d = 0.1$, $a_f = 0.1$, $x_\infty = 1$, $z_\infty = 0$ and $T_{sw} = 1$.

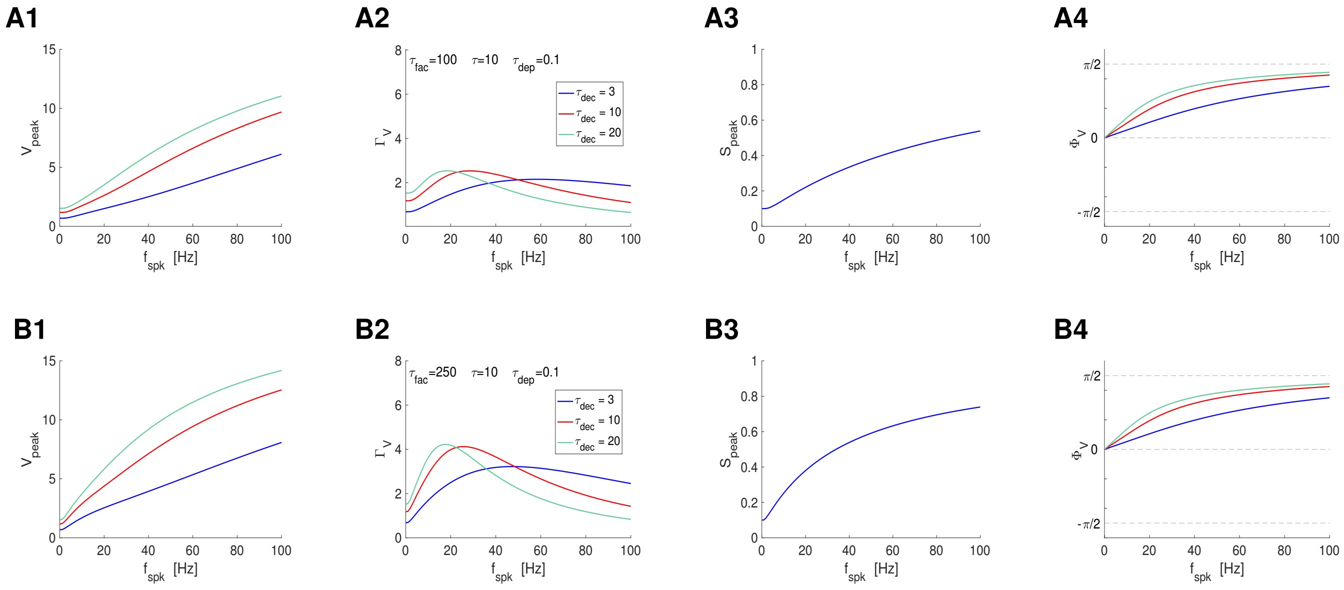

Figure S10: **Postsynaptic filters in response to periodic presynaptic spike inputs emerging from the interplay of short-term depression and postsynaptic summation.** Superimposed filters for representative values of the synaptic decay time constant τ_{dec}. **A.** $\tau_{dep} = 100$. **B.** $\tau_{dep} = 150$. **A, B.** $\tau = 10$ and $\tau_{fac} = 0.1$. **Left column.** V peak profiles. **Middle-left column.** V peak-to-trough amplitude profiles. **Middle-right column.** S peak profiles. They are independent of τ_{dec}. **Right column.** V phase profiles. We used eq. (65) for the PSP V with I_{syn} described by eqs. (2)-(4) appropriately adapted to account for the translation of V to the equilibrium point, and STP described by eqs. (12) and (13) (DA model). The impedance amplitude (Z) and phase (Φ_Z) were computed using eqs. (39) and (40). The analytical approximations for the PSP peak sequence response of passive cells to presynaptic inputs are described in Section 2.2.4 (see also Appendix A). The approximation of $V_{peak,n}$, $V_{trough,n}$ and $t_{V,peak}$ were computed as described in Section 2.2.4. The PSP amplitude Γ_V was computed by using eq. (41) and the PSP phase Φ_V was computed using eq. (42). The synaptic (S) peak (S_{peak}) and phase (Φ_S) profiles were computed similarly to these for V. We used the following additional parameter values: $C = 1$, $E_L = -60$, $I_{app} = 0$, $G_{syn} = 0.1$, $E_{syn} = 0$, $a_d = 0.1$, $a_f = 0.1$, $x_\infty = 1$, $z_\infty = 0$ and $T_{sw} = 1$.

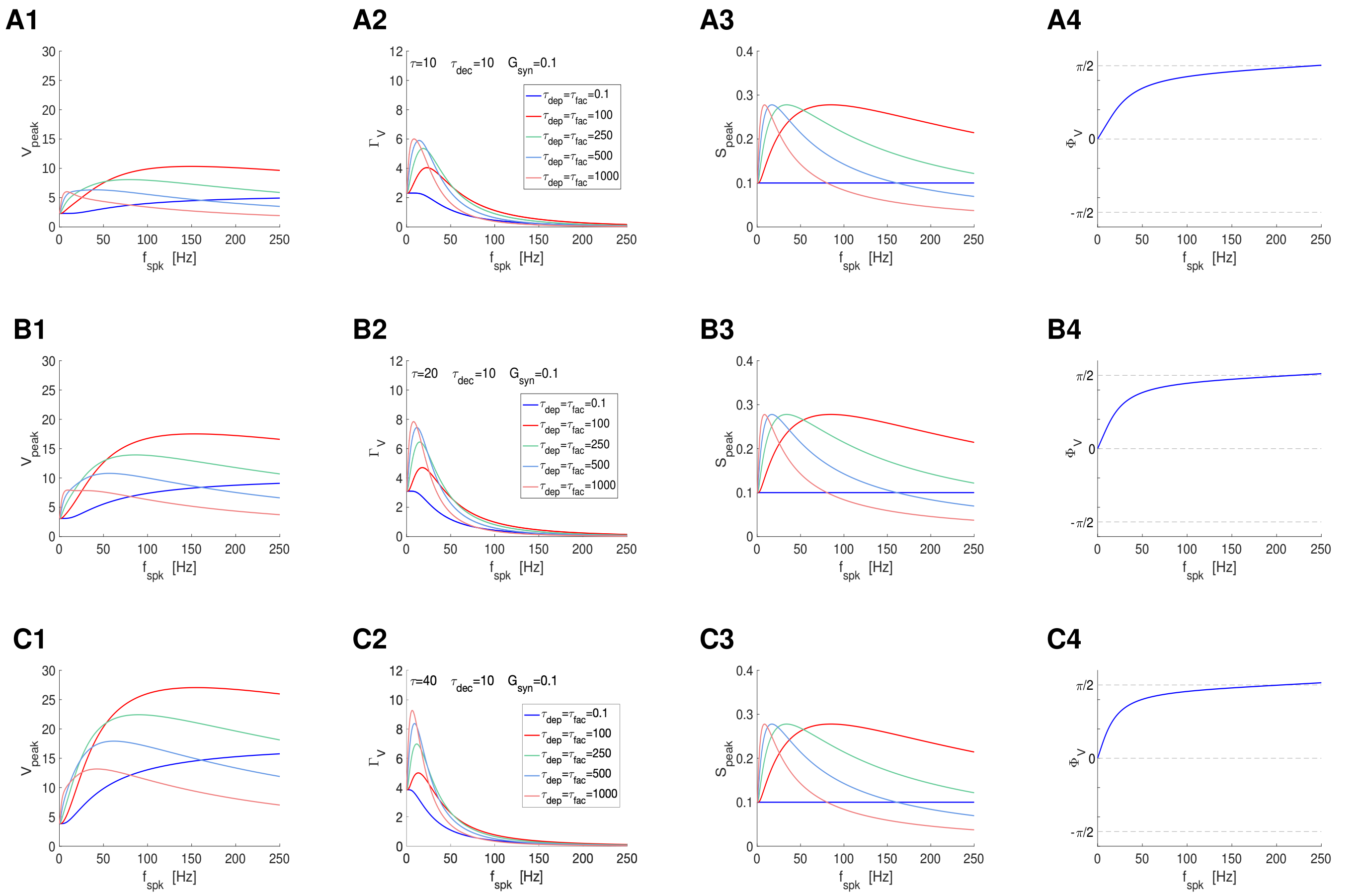

Figure S11: **Postsynaptic filters in response to periodic presynaptic spike inputs emerging from the interplay of short-term depression, facilitation and postsynaptic summation.** Superimposed filters for representative values of the depression and facilitation time constants τ_{dep} and τ_{fac}, respectively. **A.** $\tau = 10$. **B.** $\tau = 20$. **C.** $\tau = 40$. **A, B, C.** $\tau_{dec} = 10$ and $G_{syn} = 0.1$. **Left column.** V peak profiles. **Middle-left column.** V peak-to-trough amplitude profiles. **Middle-right column.** S peak profiles. **Right column.** V phase profiles. We used eq. (65) for the PSP V with I_{syn} described by eqs. (2)-(4) appropriately adapted to account for the translation of V to the equilibrium point, and STP described by eqs. (12) and (13) (DA model). The impedance amplitude (Z) and phase (Φ_Z) were computed using eqs. (39) and (40). The analytical approximations for the PSP peak sequence response of passive cells to presynaptic inputs are described in Section 2.2.4 (see also Appendix A). The approximation of $V_{peak,n}$, $V_{trough,n}$ and $t_{V,peak}$ were computed as described in Section 2.2.4. The PSP amplitude Γ_V was computed by using eq. (41) and the PSP phase Φ_V was computed using eq. (42). The synaptic (S) peak (S_{peak}) and phase (Φ_S) profiles were computed similarly to these for V. We used the following additional parameter values: $C = 1$, $E_L = -60$, $I_{app} = 0$, $G_{syn} = 0.05$, $E_{syn} = 0$, $a_d = 0.1$, $a_f = 0.1$, $x_\infty = 1$, $z_\infty = 0$ and $T_{sw} = 1$.

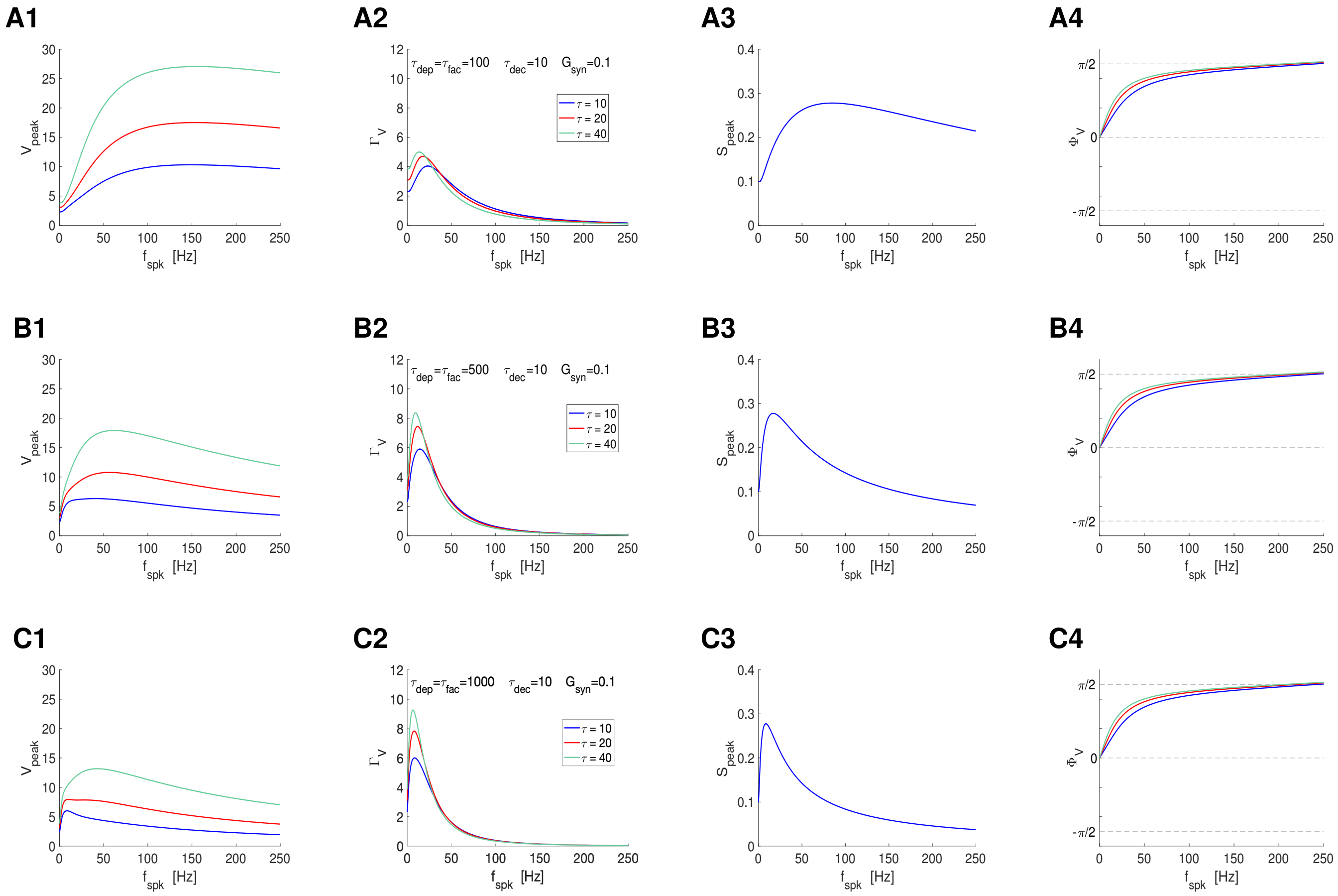

Figure S12: **Postsynaptic filters in response to periodic presynaptic spike inputs emerging from the interplay of short-term depression, facilitation and postsynaptic summation.** Superimposed filters for representative values of the membrane time constant τ. **A.** $\tau_{dep} = \tau_{fac} = 100$. **B.** $\tau_{dep} = \tau_{fac} = 500$. **C.** $\tau_{dep} = \tau_{fac} = 1000$. **A, B, C.** $\tau_{dec} = 10$ and $G_{syn} = 0.1$. **Left column.** V peak profiles. **Middle-left column.** V peak-to-trough amplitude profiles. **Middle-right column.** S peak profiles. **Right column.** V phase profiles. We used eq. (65) for the PSP V with I_{syn} described by eqs. (2)-(4) appropriately adapted to account for the translation of V to the equilibrium point, and STP described by eqs. (12) and (13) (DA model). The impedance amplitude (Z) and phase (Φ_Z) were computed using eqs. (39) and (40). The analytical approximations for the PSP peak sequence response of passive cells to presynaptic inputs are described in Section 2.2.4 (see also Appendix A). The approximation of $V_{peak,n}$, $V_{trough,n}$ and $t_{V,peak}$ were computed as described in Section 2.2.4. The PSP amplitude Γ_V was computed by using eq. (41) and the PSP phase Φ_V was computed using eq. (42). The synaptic (S) peak (S_{peak}) and phase (Φ_S) profiles were computed similarly to these for V. We used the following additional parameter values: $C = 1$, $E_L = -60$, $I_{app} = 0$, $G_{syn} = 0.05$, $E_{syn} = 0$, $a_d = 0.1$, $a_f = 0.1$, $x_\infty = 1$, $z_\infty = 0$ and $T_{sw} = 1$.

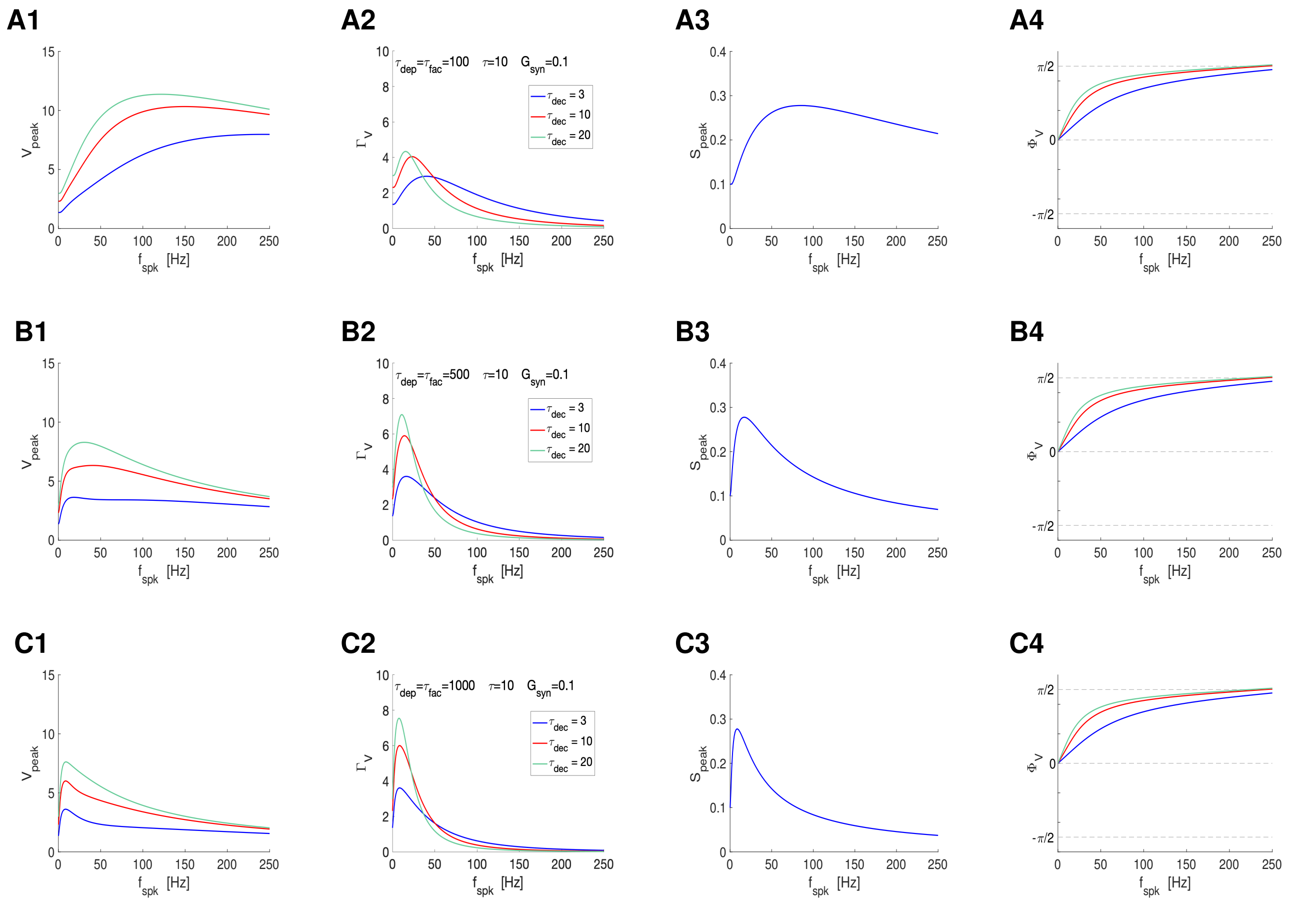

Figure S13: **Postsynaptic filters in response to periodic presynaptic spike inputs emerging from the interplay of short-term depression, facilitation and postsynaptic summation.** Superimposed filters for representative values of the depression synaptic decay time τ_{dec}. **A.** $\tau_{dep} = \tau_{fac} = 100$. **B.** $\tau_{dep} = \tau_{fac} = 500$. **C.** $\tau_{dep} = \tau_{fac} = 1000$. **A, B, C.** $\tau = 10$ and $G_{syn} = 0.1$. **Left column.** V peak profiles. **Middle-left column.** V peak-to-trough amplitude profiles. **Middle-right column.** S peak profiles. **Right column.** V phase profiles. We used eq. (65) for the PSP V with I_{syn} described by eqs. (2)-(4) appropriately adapted to account for the translation of V to the equilibrium point, and STP described by eqs. (12) and (13) (DA model). The impedance amplitude (Z) and phase (Φ_Z) were computed using eqs. (39) and (40). The analytical approximations for the PSP peak sequence response of passive cells to presynaptic inputs are described in Section 2.2.4 (see also Appendix A). The approximation of $V_{peak,n}$, $V_{trough,n}$ and $t_{V,peak}$ were computed as described in Section 2.2.4. The PSP amplitude Γ_V was computed by using eq. (41) and the PSP phase Φ_V was computed using eq. (42). The synaptic (S) peak (S_{peak}) and phase (Φ_S) profiles were computed similarly to these for V. We used the following additional parameter values: $C = 1$, $E_L = -60$, $I_{app} = 0$, $G_{syn} = 0.05$, $E_{syn} = 0$, $a_d = 0.1$, $a_f = 0.1$, $x_\infty = 1$, $z_\infty = 0$ and $T_{sw} = 1$.

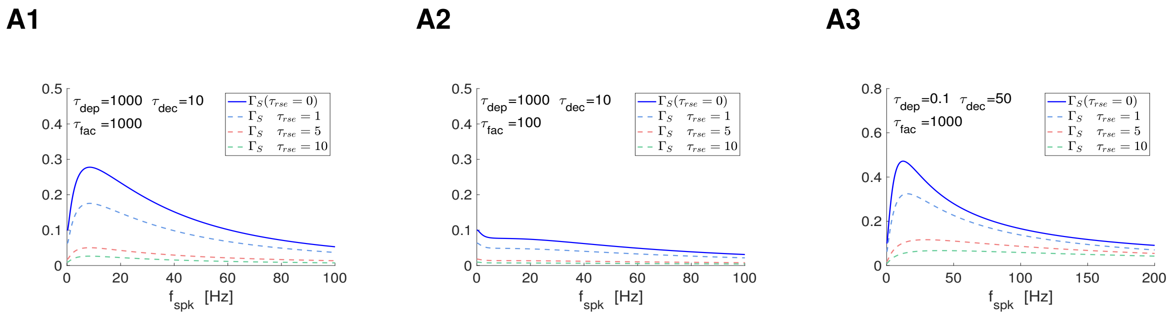

Figure S14: **Γ_S filters in response to periodic presynaptic spike inputs (frequency f_{spk}) for the to-ΔS update models with non-instantaneous update: representative examples.** We used eqs. (12) and (13) (DA model) for $\bar{\Delta S}$ **A.** To-ΔS model (synaptic update to ΔS). Effects of τ_{rse}. The S and Γ_S filters were computed using eqs. (53) and (54), respectively. **A1.** Γ_S band-pass filters attenuated by increasing values of τ_{rse}. **A2.** Γ_S low-pass filters attenuated by increasing values of τ_{rse}. **A3.** The Γ_S band-pass filter generated by the interplay of a ΔS high-pass filter and a Q_A (a low-pass filter) (Fig. 6-A6) is attenuated by increasing values of τ_{rse} and transitions to a high-pass filter. We used the following additional parameter values: $a_d = 0.1$, $a_f = 0.1$, $x_\infty = 1$, $z_\infty = 0$ and $T_{sw} = 1$.

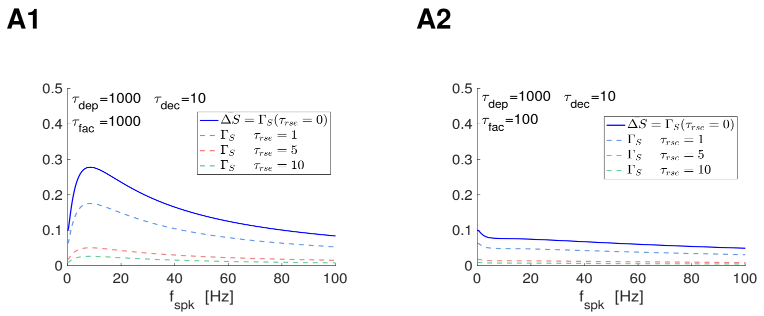

Figure S15: **Γ_S filters in response to periodic presynaptic spike inputs (frequency f_{spk}) for the by-ΔS update models with non-instantaneous update: representative examples.** We used eqs. (12) and (13) (DA model) for $\bar{\Delta S}$ **A.** By-ΔS model (synaptic update to ΔS). Effects of τ_{rse}. The S and Γ_S filters were computed using eqs. (62) and (63), respectively. **A1.** Γ_S band-pass filters attenuated by increasing values of τ_{rse}. **A2.** Γ_S low-pass filters attenuated by increasing values of τ_{rse}. We used the following additional parameter values: $a_d = 0.1$, $a_f = 0.1$, $x_\infty = 1$, $z_\infty = 0$ and $T_{sw} = 1$.

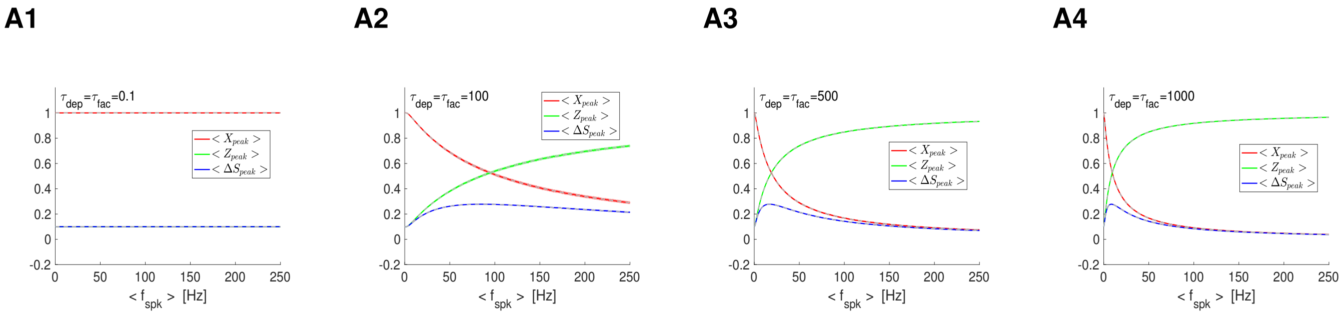

Figure S16: **X, Z and ΔS filters in response to jittered (randomly perturbed) periodic presynaptic inputs in the presence of STP: frequency- and STP-dependent variability.** For each value of the mean presynaptic input frequency $< f_{spk} >$, the ISI sequence $\{\Delta_{spk,n}\}$ ($n = 1, \dots, N_{spk}$) has the form $\Delta_{spk,n} = \Delta_{spk} + \delta_{spk,n}$ where Δ_{spk} is the ISI corresponding to f_{spk} ($f_{spk} = 1000/\Delta_{spk}$) and the sequence $\{\delta_{spk,n}\}$ are drawn from a normal distribution with zero mean and variance equal to $\delta\,\Delta_{spk}$. **A.** Superimposed X_{peak}, Z_{peak} and ΔS_{peak} profiles for representative parameter values. We used $\tau_{dec} = 10$ and $\tau = 10$ in all panels. Solid curves correspond to the mean values for each attribute (X_{peak}, Z_{peak} and ΔS_{peak}). The shadow regions correspond to one standard deviation from the mean. The dashed gray curves, almost coinciding with the solid curves, represent the corresponding deterministic profiles (response to periodic spike train inputs with frequency f_{spk}). **A1.** $\tau_{dep} = \tau_{fac} = 0.1$. **A2.** $\tau_{dep} = \tau_{fac} = 100$. **A3.** $\tau_{dep} = \tau_{fac} = 500$. **A4.** $\tau_{dep} = \tau_{fac} = 1000$. We used the following additional parameter values: $C = 1$, $E_L = -60$, $I_{app} = 0$, $E_{syn} = 0$, $a_d = 0.1$, $a_f = 0.1$, $x_\infty = 1$, $z_\infty = 0$ and $T_{sw} = 1$.

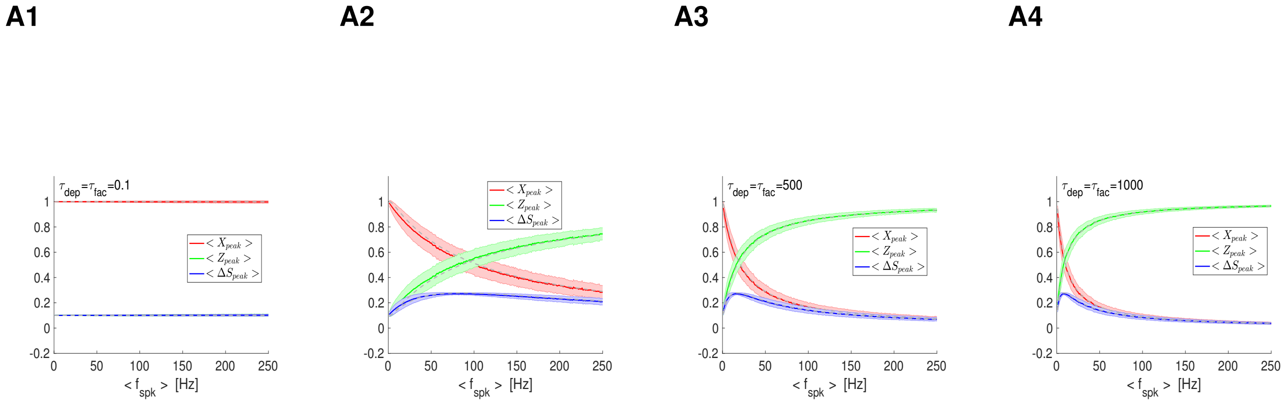

Figure S17: **X, Z and ΔS filters in response to Poisson-distributed presynaptic inputs in the presence of STP: frequency- and STP-dependent variability.** The mean rate of the Poisson distributed spike trains corresponds to $< f_{spk} >$. Superimposed X_{peak}, Z_{peak} and ΔS_{peak} profiles for representative parameter values. We used $\tau_{dec} = 10$ and $\tau = 10$ in all panels. Solid curves correspond to the mean values for each attribute (X_{peak}, Z_{peak} and ΔS_{peak}). The shadow regions correspond to one standard deviation from the mean. The dashed curves represent the corresponding deterministic profiles (response to periodic spike train inputs with frequency f_{spk}). **A1.** $\tau_{dep} = \tau_{fac} = 0.1$. **A2.** $\tau_{dep} = \tau_{fac} = 100$. **A3.** $\tau_{dep} = \tau_{fac} = 500$. **A4.** $\tau_{dep} = \tau_{fac} = 1000$. We used the following additional parameter values: $C = 1$, $E_L = -60$, $I_{app} = 0$, $E_{syn} = 0$, $a_d = 0.1$, $a_f = 0.1$, $x_\infty = 1$, $z_\infty = 0$ and $T_{sw} = 1$.
